# DIO3 coordinates photoreceptor development timing and fate stability in human retinal organoids

**DOI:** 10.1101/gad.352924.125

**Published:** 2026-01-01

**Authors:** Christina McNerney, Clayton P. Santiago, Kiara C. Eldred, Ian Glass, Tom A. Reh, Arturo Hernandez, Seth Blackshaw, Nathan D. Lord, Robert J. Johnston

**Affiliations:** 1 Department of Biology, Johns Hopkins University, Baltimore, Maryland 21218, USA;; 2 The Solomon H Snyder Department of Neuroscience, Johns Hopkins Medical Institute, Baltimore, Maryland 21218, USA;; 3 Department of Neurobiology and Biophysics, University of Washington School of Medicine, Seattle, Washington 98195, USA;; 4 Center for Molecular Medicine, MaineHealth Institute for Research, Scarborough, Maine 04074, USA;; 5 Graduate School of Biomedical Science and Engineering, University of Maine, Orono, Maine 04469, USA;; 6 Department of Medicine, Tufts University School of Medicine, Boston, Massachusetts 02111, USA;; 7 Department of Computational and Systems Biology, University of Pittsburgh, Pittsburgh, Pennsylvania 15260, USA;; 8 McGowan Institute for Regenerative Medicine, University of Pittsburgh, Pittsburgh, Pennsylvania 15260, USA

**Keywords:** retina, organoid, human, type 3 iodothyronine deiodinase, DIO3, deiodination, thyroid hormone, TH, T3, T4, photoreceptor, cone, rod, S cone, M cone, L cone, L/M cone, hourglass, hourglass hypothesis, chimeric organoid, chimera, feedback, thyroid hormone receptor β, THRB, retinal progenitor cell, RPC, S-opsin, M-opsin, L-opsin, L/M-opsin, rhodopsin, Rho, robustness, cell fate specification, multiomics, temporal, thyroid

## Abstract

The mechanisms governing the generation of neuronal subtypes at distinct times and proportions during human retinal development are poorly understood. While thyroid hormone (TH) signaling specifies cone photoreceptor subtypes, how this regulation changes over time remains unclear. To address this question, we studied the expression and function of type 3 iodothyronine deiodinase (DIO3), an enzyme that degrades TH, in human retinal organoids. We show that DIO3 is a master regulator of human photoreceptor developmental timing and cell fate stability. DIO3 is highly expressed in retinal progenitor cells (RPCs) and decreases as these cells asynchronously differentiate into neurons, progressively reducing TH degradation and increasing TH signaling. *DIO3* mutant organoids display precocious development of S cones, L/M cones, and rods; increased photoreceptor density; and subpopulations of photoreceptors that coexpress different opsin proteins. Our multiomics and chimeric organoid experiments show that cell-autonomous and non-cell-autonomous mechanisms locally coordinate and maintain DIO3 expression and TH signaling levels among cells. Computational modeling reveals a mechanism that couples TH levels and fate specification, providing robustness to photoreceptor development as compared with a probabilistic, cell-intrinsic mechanism. Based on our findings, we propose an hourglass-like mechanism in which the proportion of progenitors to neurons decreases over time to relieve TH degradation, triggering development of photoreceptor subtypes at specific times. Our study identifies how local regulation of thyroid hormone signaling influences neural cell fate specification, which may be a consideration for designing regenerative therapies.

## Abstract (precis)

In this study, McNerney et al. show that regulation of the enzyme type 3 iodothyronine deiodinase (DIO3), which degrades the thyroid hormone (TH), plays an important role in retinal photoreceptor development. They propose an “hourglass model” in which the modulation of DIO3 expression and subsequent extrinsic TH signaling between retinal progenitors and neurons influence photoreceptor developmental timing and fate specification.

The cell types of the developing retina are generated in overlapping temporal windows. Lineage tracing studies suggest that the timing of retinal cell type generation is regulated by probabilistic, cell-intrinsic mechanisms and/or extrinsic signaling (Alexiades and Cepko 1997; Belliveau and Cepko 1999; Sakagami et al. 2009; Gomes et al. 2011; He et al. 2012; Cepko 2014). Whereas cell-intrinsic mechanisms involving transcriptional regulation have been extensively studied, our understanding of how signaling controls retinal cell developmental timing is limited (Wang et al. 2005; Cepko 2014; Viets et al. 2016; Zhang et al. 2023a). Here, we investigated how signaling is regulated to control photoreceptor developmental timing in human retinal organoids.

Photoreceptors (PRs) serve essential functions in vision. During development, a subset of retinal progenitor cells (RPCs) differentiates into photoreceptor precursors that express the transcription factor CRX (Furukawa et al. 1997). These precursors then develop into one of two PR classes: rods or cones. Rods are specialized for low-light (i.e., scotopic) vision. During early development, rods express the transcription factors NRL and NR2E3 (Mears et al. 2001; Chen et al. 2005), followed by rhodopsin (Rho) as they mature. Cones provide high-acuity daytime (i.e., photopic) and trichromatic color vision in humans. Cones begin to express THRB early (Ng et al. 2001) and color-sensitive opsins as they terminally differentiate. Cones are further classified into three subtypes based on the opsin they express: S cones (short wavelength; blue), M cones (medium wavelength; green), or L cones (long wavelength; red). Cone subtypes are diversified by two fate decisions: (1) S versus L/M fates and (2) L versus M fates (Nathans et al. 1986). In the developing human retina, S-opsin is expressed before L/M-opsin and Rho (Curcio et al. 1990; Xiao and Hendrickson 2000). Here, we investigated the developmental timing and fate specification of human S cones, L/M cones, and rods.

Thyroid hormone (TH) signaling regulates cone subtype specification and development across species (Ng et al. 2001, 2009, 2010, 2011, 2017; Roberts et al. 2006; Lu et al. 2009; Ma et al. 2014; Mackin et al. 2019; McNerney and Johnston 2021; Aramaki et al. 2022). We previously showed that terminal S-cone and L/M-cone fates are regulated by TH signaling in human retinal organoids (Eldred et al. 2018). Thyroid hormone receptor B (THRB) is a nuclear hormone receptor that mediates TH signaling. *THRB*Δ mutant organoids display a loss of L/M cones, suggesting that THRB is required for L/M-cone fate. TH has two forms: the less active, circulating T4 and the highly active T3. Wild-type organoids grown in high T3 conditions have a high density of L/M cones and a low density of S cones on day 200. When wild-type organoids are exposed to high T3 conditions after the generation of S-opsin-expressing cones, these same cells begin to express L/M-opsin, suggesting cell fate plasticity (Hussey et al. 2024). While TH signaling is critical for fate specification, how the retina regulates TH signaling to control the timing of cone subtype development remains unclear.

TH regulation occurs on both organismal and local scales. At the organismal scale, the hypothalamus–anterior pituitary–thyroid (HPT) axis controls TH levels. The hypothalamus secretes thyrotropin-releasing hormone (TRH), which promotes the anterior pituitary to release thyroid-stimulating hormone (TSH). TSH acts on the thyroid gland to promote production and release of TH. Circulating TH then regulates the hypothalamus and anterior pituitary in a negative feedback loop: Low TH leads to increased TH production, while high TH suppresses TH production (Larsen 1982; Gelfand et al. 1987; Alkemade et al. 2005; Fliers et al. 2006; Dietrich et al. 2012; Fekete and Lechan 2014; Feldt-Rasmussen et al. 2021). On the local scale, additional regulation occurs in the thyroid and target tissues, such as the liver, which are capable of activating circulating T4 into T3 or degrading TH (both T3 and T4) to modulate TH signaling (Bianco et al. 2002; Gereben et al. 2008a; Müller et al. 2014; Galton 2017). These processes are mediated by deiodinase enzymes, which deiodinate THs via a selenocysteine-containing active site to either activate (DIO1 and DIO2) or degrade TH (DIO1 and DIO3) (Hernandez et al. 2002; Gereben et al. 2008a,b).

In mice, *Dio3* is expressed in the early developing retina and decreases over time (Ng et al. 2010, 2017). *Dio3*Δ mutant mice display increased cone death, suggesting that elevated TH levels are detrimental to PR and retinal development in mice (Roberts et al. 2006; Lu et al. 2009; Ng et al. 2009, 2010, 2011, 2017; Ma et al. 2014). In the developing chick retina, *DIO3* is expressed in two waves before decreasing over time (Trimarchi et al. 2008). Similarly, we previously showed that in human fetal retinas and retinal organoids, *DIO3* mRNA is highly expressed early and then gradually decreases as development progresses (Eldred et al. 2018).

The dynamic expression of DIO3, its role in TH degradation, and the influence of TH signaling on cone subtype specification suggest that DIO3 regulates the timing of human photoreceptor development. However, this function has not been explored, partly due to the challenges of obtaining fetal tissue. Stem cell-derived human retinal organoids provide a genetically and pharmacologically tractable model to interrogate mechanisms of human development. Previous studies characterized extensive similarities between the fetal retina and organoid development, establishing the utility of organoids for investigating cell fate specification and developmental timing (Eiraku et al. 2011; Nakano et al. 2012; Kaewkhaw et al. 2015; Wahlin et al. 2017; Eldred et al. 2018; Sridhar et al. 2020; Hadyniak et al. 2024; Hussey et al. 2024).

In this study, we sought to determine how regulation of TH signaling and the timing of PR development are linked during human retinal organoid development. As *DIO3* expression decreases during human retinal and organoid development (Eldred et al. 2018), we hypothesized that loss of TH degradation increases TH signaling, driving PR development over time.

To test this hypothesis, we examined how DIO3 controls the temporality of PR development. DIO3 is expressed in RPCs, and its expression and activity decrease as RPCs differentiate. *DIO3* mutant human organoids display precocious cone and rod development, increased PR density, and nonexclusive PR fates. Importantly, both cell-autonomous and non-cell-autonomous mechanisms regulate DIO3 levels to coordinate TH signaling and PR development locally in the retina. Thus, as the RPC:neuron proportion decreases over time, TH degradation is relieved, progressively increasing TH signaling to trigger PR subtype development.

## Results

### DIO3 is expressed in RPCs and decreases in neurons in fetal retinas and organoids

**Figure 1.**
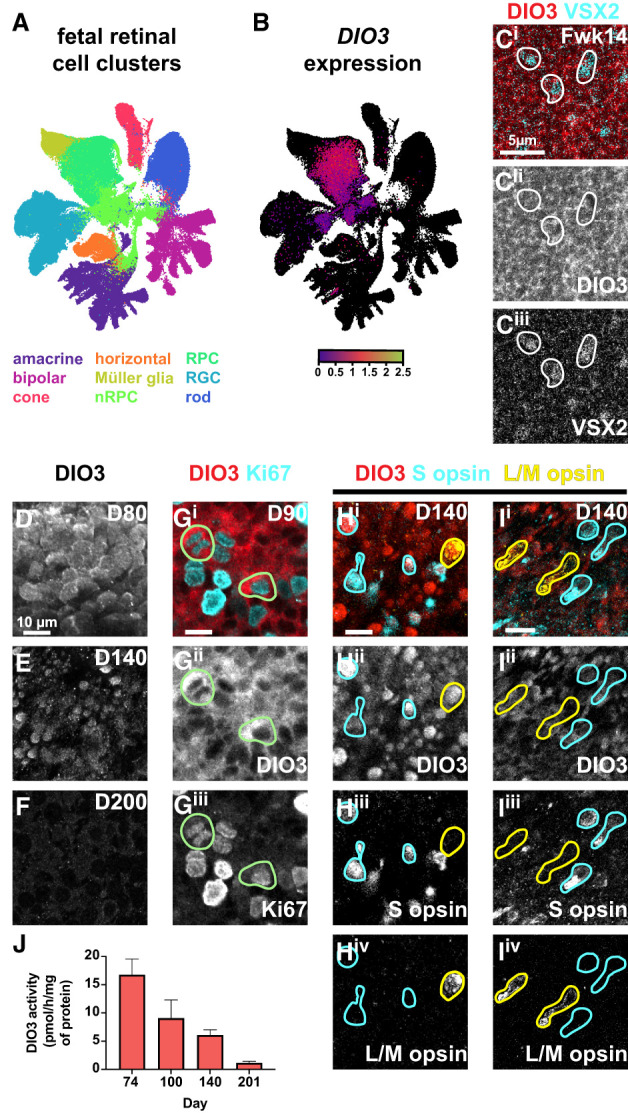
DIO3 is expressed in RPCs in the developing human retina and retinal organoids. (*A*) UMAP from CZ CellxGene Discover (Zuo et al. 2024) of human fetal retina scRNA-seq with major cell types labeled. (*B*) *DIO3* mRNA expression in fetal retina is limited to DIO3^+^ RPCs, overlaid on the unannotated UMAP. (*C*) DIO3 is expressed in VSX2^+^ cells in a 14 week old fetal retina. (*D–F*) DIO3 expression in human retinal organoids at days 80 (*D*), 140 (*E*), and 200 (*F*). (*G*) DIO3 and Ki67 expression in human retinal organoids at day 90. Green circles indicate Ki67^+^/DIO3^+^ cells. (*H*) DIO3 and cone opsin expression in human retinal organoids at day 140. Yellow outlines indicate L/M-opsin^+^ cells. Blue outlines indicate S-opsin^+^ cells. (*H*) Region of morphologically naive photoreceptors within an organoid (same organoid as in *I*). (*I*) Region of morphologically more mature photoreceptors within an organoid (same organoid as in *H*). (*J*) DIO3 activity as measured by deiodination activity of wild-type organoids over time. Error bars represent SEM.

To determine how *DIO3* expression changes during human retinal development, we examined a published single-nucleus RNA-seq (snRNA-seq) data set of the developing human fetal retina (Zuo et al. 2024) and found that *DIO3* mRNA is highly expressed in the RPC cluster (Fig. 1A,B), identified by coexpression of *VSX2* and *PAX6* (Supplemental Fig. S1A–C). We also observed DIO3 protein expression in VSX2^*+*^ RPCs in a 14 week old fetal retina (Fig. 1C). In the snRNA-seq data set, minimal *DIO3* mRNA expression was observed in mature neurons (Fig. 1A,B). These data suggest that DIO3 is expressed in RPCs and decreases as RPCs differentiate into terminal retinal cells.

As accessibility and experimental tractability of human fetal retinal tissue are limited, we studied human retinal organoids to evaluate the dynamics of DIO3 expression. We visualized DIO3 protein on days 80, 90, 140, and 200 of organoid development (Fig. 1D–I). On days 80 and 90, DIO3 was highly expressed in RPCs (Fig. 1D,G). On day 140, DIO3 was highly expressed in a subset of cells but was low in others (Fig. 1E). On day 200, DIO3 was minimal across all cells (Fig. 1F). These data suggest that the proportion of retinal cells expressing DIO3 decreases during development.

We next related cell type specificity to temporal dynamics of DIO3 expression. In organoids on day 90, DIO3 was observed in cells that express Ki67 (Fig. 1G), which marks mitotic cells. This observation suggests that DIO3 is expressed in mitotic RPCs in organoids, similar to *DIO3* mRNA in fetal retinas (Fig. 1A,B). To determine the relationship between DIO3 and cone differentiation, we examined expression of DIO3 in cells that express S-opsin or L/M-opsin. We determined the maturity of these cells by comparing morphological characteristics of each cell within the same organoid. Whereas immature cones had round cell bodies with an even distribution of opsin throughout the cell, mature cones had elongated processes and opsin localized to putative inner segments. In organoids on day 140, DIO3 was expressed at higher levels in immature S-opsin-expressing cones and L/M-opsin-expressing cones (Fig. 1H) and at lower levels in mature S-opsin-expressing cones and L/M-opsin-expressing cones (Fig. 1I). Finally, to relate DIO3 expression and function, we measured DIO3 deiodination activity in retinal organoids and found that it decreases during development (Fig. 1J), consistent with the progressive reduction in DIO3 protein expression.

These results suggest that DIO3 is expressed in RPCs and decreases as RPCs differentiate into neurons. As the proportion of DIO3-expressing cells decreases, DIO3 activity decreases.

### Generation of DIO3 mutant organoids

To investigate the functional role of DIO3 in human retinal organoids, we used CRISPR/Cas9 to generate a stem cell line with a homozygous 4 bp deletion that introduced an early stop, yielding a presumptive null mutation (*DIO3*^*0*^) (Supplemental Fig. S1D,E). We differentiated *DIO3^0^-*null mutant organoids, which did not generate retinal tissue, as indicated by the absence of optic cup formation and lamination (zero of 384 aggregates developed retinal tissue across two independent differentiations), while wild-type control organoids successfully differentiated in parallel (Supplemental Fig. S1F–H). This suggests that *DIO3* is required for retinal tissue development in organoids.

**Figure 2.**
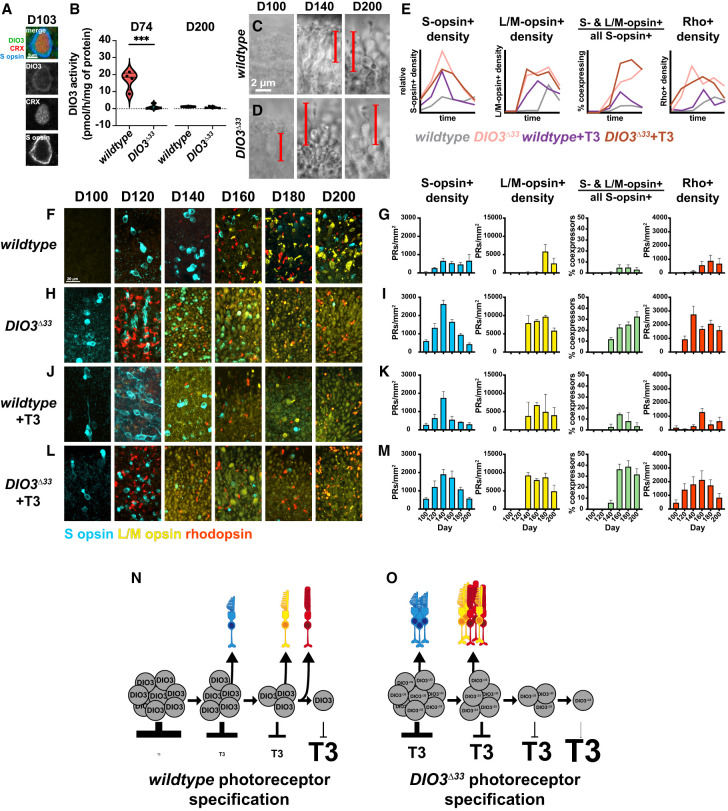
*DIO3*^Δ*33*^ mutant and +T3 conditions accelerate PR development, increase PR quantities, and yield mixed PR fates. (*A*) DIO3 expression in an S-opsin^+^, CRX^+^ day 103 *DIO3*^Δ*33*^ organoid cell. (*B*) DIO3 activity of wild type and *DIO3*^Δ*33*^ at days 74 and 201 of differentiation. At day 74, *P* = 0.0004; at day 201, *P* = 0.2225 (ns) by unpaired *t*-test. (*C*,*D*) Bright-field imaging of wild-type (*C*) and *DIO3*^Δ*33*^ (*D*) organoids at days 100 (*left*), 140 (*middle*), and 200 (*right*). Bracketed lines highlight individual PR precursors or PRs. (*E*) Graphical representation of quantifications shown in *G*, *I*, *K*, and *M*. The *Y*-axes are scaled relative to each opsin. (*F*,*H*,*J*,*L*) Timeline of opsin expression in human retinal organoids at days 100, 120, 140, 160, 180, and 200 in wild-type organoids (*F*), *DIO3*^Δ*33*^ mutant retinal organoids (*H*), wild-type +T3 organoids (*J*), and *DIO3*^Δ*33*^ +T3 organoids (*L*). (*G*,*I*,*K*,*M*) Quantification of S-opsin^+^ cell density (blue), L/M-opsin^+^ cell density (yellow), percentage of S-opsin^+^ cells expressing L/M-opsin (green), and Rho^+^ cell density (red) for wild-type organoids (*G*), *DIO3*^Δ*33*^ mutant retinal organoids (*I*), +T3-treated wild-type organoids (*K*), and *DIO3*^Δ*33*^ +T3 organoids (*M*). Error bars represent SEM. Wild type: D100 *n* = 5, D120 *N* = 8, D140 *N* = 4, D160 *N* = 4, D180 *N* = 6, D200 *N* = 4. *DIO3*^Δ*33*^: D100 *N* = 5, D120 *N* = 9, D140 *N* = 9, D160 *N* = 10, D180 *N* = 17, D200 *N* = 11. Wild type +T3: D100 *N* = 5, D120 *N* = 3, D140 *N* = 3, D160 *N* = 3, D180 *N* = 2, D200 *N* = 4. *DIO3*^Δ*33*^ +T3: D100 *N* = 4, D120 *N* = 3, D140 *N* = 4, D160 *N* = 4, D180 *N* = 3, D200 *N* = 4. Data are as in Supplemental Figure S2, which is organized by opsin expression phenotype and time point. Supplemental Figure S2 also reports our statistical analysis. (*N*) Model of wild-type photoreceptor specification, in which DIO3-expressing RPC populations decrease and T3 levels increase during development. (*O*) Model of *DIO3*^Δ*33*^ photoreceptor specification, in which the *DIO3*^Δ*33*^-expressing RPC population has impaired T3 degradation, leading to high T3 levels. L/M cones and rods are generated early and at higher densities than in wild type, with some rods coexpressing L/M-opsin.

To circumvent this requirement, we generated the *DIO3*^Δ*33*^*/+* stem cell line, which is heterozygous for a wild-type allele and a 33 bp, in-frame deletion (*DIO3*^Δ*33*^) (Supplemental Fig. S1I), causing a loss of 11 amino acids adjacent to but not including the active site (Supplemental Fig. S1J). We differentiated *DIO3*^Δ*33*^*/+* organoids, which developed normal optic cup-like morphologies and lamination (Supplemental Fig. S1K). In *DIO3*^Δ*33*^*/+* organoids, DIO3 protein remained detectable by IHC on day 100 (Fig. 2A), consistent with the generation of DIO3 protein that retained the antigenic epitope (epitope positions 250–300) (Supplemental Fig. S1J).

To assess changes in DIO3 enzymatic activity in *DIO3*^Δ*33*^*/+* organoids, we evaluated deiodination activity in wild-type and *DIO3*^Δ*33*^*/+* organoids on day 74, when *DIO3* mRNA and protein are highly expressed (Fig. 1D,G; Eldred et al. 2018). Deiodination activity in *DIO3*^Δ*33*^*/+* was reduced by ∼94% compared with wild-type organoids (wild type = 16.8 pmol/h/mg of protein, *DIO3*^Δ*33*^*/+* = 1.0 pmol/h/mg of protein, *P* < 0.001) (Fig. 2B). Because the *DIO3*^Δ*33*^*/+* organoids are heterozygous, this >50% reduction in deiodination activity suggests that the *DIO3*^Δ*33*^*/+* mutation has a dominant-negative phenotype. We observed a similar decrease in DIO3 function in 76 day old wild-type and *DIO3*^Δ*33*^*/+* organoids derived from an independent differentiation (wild type = 17.4 pmol/h/mg of protein, *DIO3*^Δ*33*^*/+* = 1.0 pmol/h/mg of protein, *P* < 0.01) (Supplemental Fig. S1L). We also measured deiodination activity in wild-type and *DIO3*^Δ*33*^*/+* organoids on day 200 and observed low activity in both (wild type = 1.2 pmol/h/mg of protein, *DIO3*^Δ*33*^*/+­* = 0.7 pmol/h/mg of protein, ns) (Fig. 2B), consistent with the reduction in DIO3 activity and expression in wild-type organoids at this late time point (Fig. 1F,I) and the decreased deiodination function in *DIO3*^Δ*33*^*/+.* Notably, the remaining, minimal DIO3 activity permits retinal tissue generation, allowing for characterization of mutant phenotypes. We refer to this stem cell line as “*DIO3*^Δ*33*^ mutant” for simplicity.

### *DIO3*^Δ*33*^ mutants display changes in PR developmental timing, quantity, and stability

To determine the roles of DIO3 and T3 in PR development, we differentiated organoids in parallel in four conditions: wild type, *DIO3*^Δ*33*^, wild-type *+*T3, and *DIO3*^Δ*33*^ *+*T3. We evaluated PR development based on the densities of S-opsin-expressing (S-opsin^+^) cells, L/M-opsin-expressing (L/M-opsin^+^) cells, and Rho-expressing (Rho^+^) cells on days 100, 120, 140, 160, 180, and 200. We also observed and quantified cells that coexpressed S-opsin and L/M-opsin (percentage of S-opsin^+^-coexpressing L/M-opsin) and Rho and L/M-opsin (percentage of Rho^+^-coexpressing L/M-opsin).

To assess how DIO3 regulates PR developmental timing, we imaged wild-type and *DIO3*^Δ*33*^ mutant organoids with bright-field microscopy (Fig. 2C,D). At day 100, wild-type organoids had no visible PRs (Fig. 2C, D100), whereas *DIO3*^Δ*33*^ organoids had immature PRs with elongated, goblet-shaped cell bodies (Fig. 2D, D100). At day 140, wild-type organoids had a low density of immature PRs (Fig. 2C, D140), while *DIO3*^Δ*33*^ organoids had mature PRs with outer segments (Fig. 2D, D140). At day 200, both wild-type (Fig. 2C, D200) and *DIO3*^Δ*33*^ (Fig. 2D, D200) organoids had dense PRs with mature morphologies. These observations suggest that PR development is accelerated in *DIO3*^Δ*33*^ mutant organoids.

To investigate PR subtype development in *DIO3*^Δ*33*^ mutants, we next compared developmental timing of S-opsin^+^, L/M-opsin^+^, and Rho^+^ cells in wild-type and *DIO3*^Δ*33*^ mutant organoids with IHC of opsins (Fig. 2E–I; statistics reported in Supplemental Fig. S2). At day 100, wild-type organoids contained very few S-opsin^+^ cells (Fig. 2E–G, D100; Supplemental Fig. S2A). In contrast, *DIO3*^Δ*33*^ organoids displayed significantly more S-opsin^+^ cells (S-opsin^+^, wild type = 39 PRs/mm^2^, *DIO3*^Δ*33*^ = 602 PRs/mm^2^, *P* < 0.001) (Fig. 2E,H,I, D100; Supplemental Fig. S2A), suggesting that development of S-opsin^+^ cells is accelerated in *DIO3*^Δ*33*^ mutants.

At day 120, wild-type organoids contained morphologically immature S-opsin^+^ cells (Fig. 2E–G, D120), whereas *DIO3*^Δ*33*^ organoids displayed an increase in S-opsin^+^ cells, which localized to the outer nuclear layer (ONL) and had inner segments, indicating maturity (S-opsin^+^, wild type = 259 PRs/mm^2^, *DIO3*^Δ*33*^ = 1325 PRs/mm^2^, *P* < 0.01) (Fig. 2E,H,I, D120; Supplemental Fig. S2A). *DIO3*^Δ*33*^ organoids also contained a small population of L/M-opsin^+^ cells (L/M-opsin^+^, wild type = 0 PRs/mm^2^, *DIO3*^Δ*33*^ = 12 PRs/mm^2^, ns) and a larger population of Rho^+^ cells (Rho^+^, wild type = 42 PRs/mm^2^, *DIO3*^Δ*33*^ = 925 PRs/mm^2^, *P* < 0.05) (Fig. 2E,H,I, D120; Supplemental Fig. S2B,D), suggesting that development of L/M-opsin^+^ and Rho^+^ cells is also accelerated in *DIO3*^Δ*33*^ mutants.

At day 140, wild-type organoids exhibited increased densities of S-opsin^+^ cells and few L/M opsin^+^ and Rho^+^ cells (Fig. 2E–G, D140; Supplemental Fig. S2A,B,D). In contrast, *DIO3*^Δ*33*^ organoids displayed dramatic increases in S-opsin^+^, L/M-opsin^+^, and Rho^+^ cells (S-opsin^+^, wild type = 653 PRs/mm^2^, *DIO3*^Δ*33*^ = 2632 PRs/mm^2^, *P* < 0.001; L/M-opsin^+^, wild type = 190 PRs/mm^2^, *DIO3*^Δ*33*^ = 7936 PRs/mm^2^, ns; Rho^+^, wild type = 99 PRs/mm^2^, *DIO3*^Δ*33*^ = 2746 PRs/mm^2^, *P* < 0.05) (Fig. 2E,H,I, D140; Supplemental Fig. S2A,B,D). A subset of L/M-opsin^+^ cells had simpler morphologies and lower L/M-opsin expression (Fig. 2H, D140). These L/M-opsin^+^ cells expressed ARR3 (cone gene) (Supplemental Fig. S3A–D), CRX (PR precursor and PR gene) (Supplemental Fig. S3A–D), and RCVRN (PR gene) (Supplemental Fig. S3E–H) but not NRL (rod gene) (Supplemental Fig. S3E–H), confirming their identity as L/M cones. These cells developed outer segments over time (Supplemental Fig. S3J). The length of the outer segments (Supplemental Fig. S3K) and the proportion of lowly expressing L/M-opsin^+^ cells with outer segments (Supplemental Fig. S3L) increased more rapidly in *DIO3*^Δ*33*^ mutants compared with wild-type organoids.

The densities of all PR types were greater in *DIO3*^Δ*33*^ mutant organoids at D140 than at any time point in wild-type organoid development, suggesting that PR generation is increased in *DIO3*^Δ*33*^ mutants (Supplemental Fig. S2A,B,D).

At day 140, *DIO3*^Δ*33*^ organoids developed a population of S-opsin^+^ cells that coexpressed L/M-opsin (S-opsin^+^ and L/M-opsin^+^, wild type = 1%, *DIO3*^Δ*33*^ = 12%, *P* < 0.01) (Fig. 2E,H,I, D140; Supplemental Fig. S3I,L). We also observed a population of Rho^+^ cells that coexpressed L/M-opsin (L/M-opsin^+^ and Rho^+^, wild type = 0%, *DIO3*^Δ*33*^ = 43%, *P* < 0.0001) (Supplemental Figs. S2E, S3M–T). These results suggest that photoreceptor fate stability is disrupted in *DIO3*^Δ*33*^ organoids.

Across days 160, 180, and 200, wild-type organoids maintained S-opsin^+^ cell density and increased L/M-opsin^+^ and Rho^+^ cell densities (Fig. 2E–G, D160, D180, and D200; Supplemental Fig. S2A,B,D). In contrast, *DIO3*^Δ*33*^ organoids displayed a dramatic decrease in the density of S-opsin^+^ cells, an increase in the populations of S-opsin^+^ or Rho^+^ cells that coexpressed L/M-opsin, and similar densities of L/M-opsin^+^ and Rho^+^ cells (Fig. 2E,H,I, D160, D180, D200; Supplemental Fig. S2). The decrease in S-opsin^+^ and Rho^+^ cells from their peaks on day 140 together with the increase in the percentage of coexpressing cells suggests that subsets of S-opsin^+^ cells and Rho^+^ cells are converted to L/M-cone fate or die. The density of L/M-opsin^+^ cells decreased on day 200 (Fig. 2E,H,I, D200; Supplemental Fig. S2), suggesting that cell fate conversion is limited and/or that a subpopulation of these cells die.

Together, *DIO3*^Δ*33*^ organoids displayed accelerated and increased generation of S-opsin^+^, L/M-opsin^+^, and Rho^+^ cells as well as nonexclusive opsin expression (Fig. 2E,N,O; Supplemental Figs. S2, S3M–T). PR density was higher in *DIO3*^Δ*33*^ organoids than in wild-type organoids at all examined time points (Supplemental Fig. S3U,V). The highest PR density in *DIO3*^Δ*33*^ organoids at day 140 exceeded that of wild-type organoids at any time point (Supplemental Fig. S3U,V). These data suggest that DIO3 functions to control the timing, quantity, and stability of PR development.

### Organoids grown in high T3 conditions display developmental changes similar to *DIO3*^Δ*33*^ mutant organoids

Because DIO3 degrades T3, *DIO3*^Δ*33*^ mutant organoids likely have elevated TH signaling. Therefore, wild-type organoids grown in high exogenous T3 conditions (wild type +T3) should show changes in PR timing, quantities, and fate stability similar to *DIO3*^Δ*33*^ mutant organoids. In wild-type organoids grown in high T3 conditions, S-opsin^+^, L/M-opsin^+^, and Rho^+^ cells were generated earlier and in higher quantities (Fig. 2E,J,K; Supplemental Fig. S2A,B,D). Over time, S-opsin^+^ cells decreased, while L/M-opsin^+^ cells and the proportion of S-opsin^+^ cells coexpressing L/M-opsin increased (Fig. 2E,J,K; Supplemental Fig. S2A–C). The proportion of Rho^+^ cells coexpressing L/M-opsin increased at D160 but decreased over the rest of development (Supplemental Figs. S2E, S3P,Q,S). Wild-type +T3 organoids displayed less severe changes compared with *DIO3*^Δ*33*^ organoids, likely due to buffering by endogenous, functional DIO3. These observations confirm that the developmental changes observed in *DIO3*^Δ*33*^ mutant organoids are driven by increased TH signaling.

We next tested how addition of T3 affected *DIO3*^Δ*33*^ mutant organoids. *DIO3*^Δ*33*^ mutant organoids grown in high T3 conditions from day 42 to 200 (*DIO3*^Δ*33*^+T3) displayed phenotypes similar to that of *DIO3*^Δ*33*^ mutant organoids alone (Fig. 2E,L,M; Supplemental Figs. S2, S3P,Q,T), consistent with both perturbations affecting the same pathway.

Together, these data suggest that the phenotypes observed in *DIO3*^Δ*33*^ mutant organoids are due to elevated T3 signaling.

### *DIO3*^Δ*33*^ mutant PRs are more mature than wild-type PRs on day 200

**Figure 3.**
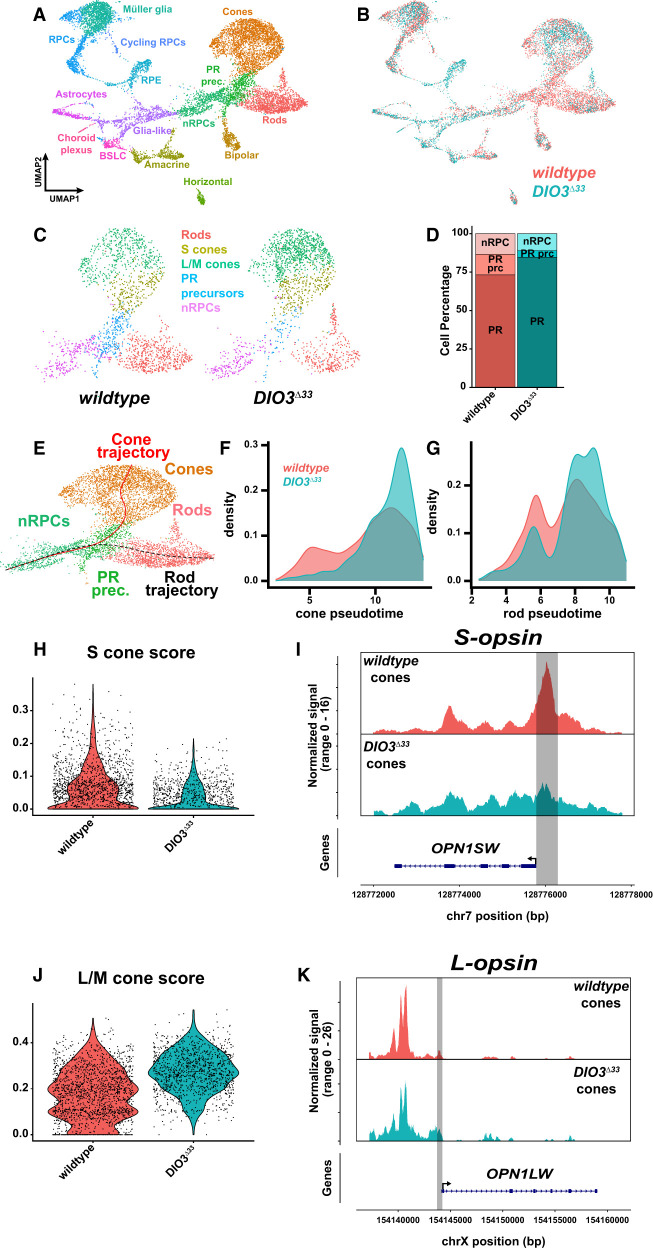
Single-nucleus multiomics analysis suggests increased PR maturity and enrichment of L/M-cone fate in *DIO3*^Δ*33*^ organoids. (*A*) Combined UMAP of D200 *DIO3*^Δ*33*^ and wild-type organoids. Clusters are annotated and color-matched with cell types. (*B*) Cells colored by tissue origin. (Coral) Wild-type controls, (teal) *DIO3*^Δ*33*^. (*C*) Zoomed-in view of photoreceptor lineages from *A* and *B*, split by genotype. Neurogenic retinal progenitor cells (nRPCs) are in purple, photoreceptor (PR) precursors are in blue, rods are in red, L/M cones are in green, and S cones are in gold. Cell populations were downsampled to visually compare proportions; all other comparisons were performed on complete, nondownsampled data. (*D*) Proportions of cell states in photoreceptor lineages. (nRPCs) Neurogenic RPCs, (PR prc) PR precursors, (PR) terminal PRs. (*E*) Predicted developmental trajectories of photoreceptors. (*F*,*G*) Pseudotime density plots of cones (*F*) and rods (*G*). The *X*-axis represents the distance along pseudotime trajectory as identified in *E*, excluding nRPCs. (*H*,*J*) S-cone (*H*) or L/M-cone (*J*) scores applied to cones in wild type (coral) and *DIO3*^Δ*33*^ (teal). (*I*,*K*) Chromatin accessibilities of *S-opsin* (*I*) and *L-opsin* (*K*) genes. (*Top*) Tracks separated by genotype. (*Bottom*) Gene annotation. Gray regions outline promoters as defined by 500 bp upstream of the start site.

Our analysis of *DIO3*^Δ*33*^ mutant organoids suggested that PR development is accelerated. To determine whether *DIO3*^Δ*33*^ mutant PRs are more mature than wild-type PRs, we conducted snRNA-seq and snATAC-seq multiomics of wild-type and *DIO3*^Δ*33*^ organoids on day 200. Clustering based on snRNA-seq alone, snATAC-seq alone, or both identified neural retinal cells including RPCs and all major classes of retinal cells (i.e., cones, rods, amacrine cells, horizontal cells, bipolar cells, and Müller glia), except for RGCs, which are lost in organoids by day 200 (Fig. 3A; Supplemental Fig. S4A–E; Cowan et al. 2020; Sridhar et al. 2020; Eldred and Reh 2021). We also identified nonneural retinal cells including RPE cells, glia-like cells, astrocytes, and choroid plexus cells, as well as nonretinal brain and spinal cord-like (BSL) cells, which we previously found in transplanted organoids using scRNA-seq analysis (Fig. 3A; Supplemental Fig. S4A–D; Liu et al. 2023).

Because clustering based on snRNA-seq, snATAC-seq, or both resulted in similar outcomes (Supplemental Fig. S4A–C), we initially focused on snRNA-seq-based clustering (Fig. 3A,B) and retinal cells (Supplemental Fig. S4E). We identified populations of neurogenic RPCs (nRPCs), postmitotic PR precursors, and terminally differentiated PRs in wild-typ*e* and *DIO3*^Δ*33*^ organoids (Fig. 3C; Supplemental Fig. S4F). While wild-type and *DIO3*^Δ*33*^ organoids had similar proportions of nRPCs (Fig. 3C,D), *DIO3*^Δ*33*^ organoids had mature PRs at the expense of PR precursors (Fig. 3C,D), reflecting accelerated PR development in *DIO3*^Δ*33*^ mutants.

Given the acceleration of PR development based on opsin expression in *DIO3*^Δ*33*^ mutants, we analyzed the developmental trajectories of PRs using the snRNA-seq data. We mapped developmental trajectories of PRs, starting from nRPCs, progressing to PR precursors, and then splitting into either rod or cone fates (Fig. 3E), consistent with previous studies (Brzezinski and Reh 2015; Lu et al. 2020; Lyu et al. 2021). To assess developmental maturity, we generated pseudotime density plots for both the cone and rod trajectories. In both cases, a larger proportion of *DIO3*^Δ*33*^ cells clustered at more advanced pseudotime states compared with wild-type cells (Fig. 3F,G), suggesting that cones and rods are more mature in *DIO3*^Δ*33*^ mutants. The increased number and advanced maturation of PRs in *DIO3*^Δ*33*^ organoids were consistent with our IHC analysis (Fig. 2E–M; Supplemental Fig. S2).

### *DIO3*^Δ*33*^ mutant PRs are more L/M-cone-like

Our analysis of *DIO3*^Δ*33*^ mutant organoids suggested that a subset of S cones adopt L/M-cone fate character. In our previous scRNA-seq analysis of human retinal organoids, S-cone, L/M-cone, and S-cone and L/M-cone subtype clusters were not directly identifiable (Hussey et al. 2024). To assess the fates of cones, we generated S-cone and L/M-cone scores by analyzing the differential expression of genes associated with these cone fates across all cones in humans (Lu et al. 2020), nonhuman primates (Peng et al. 2019), and cows (Zhang et al. 2019) and applied these scores to each genotype. When visualized on UMAP plots, the S-cone and L/M-cone scores (Supplemental Fig. S4G,I), as well as a rod score (Supplemental Fig. S4L), correlated with subtype-specific gene expression (Supplemental Fig. S4H,J,K,M), validating our annotation strategy. *DIO3*^Δ*33*^ mutant cones had a lower S-cone score and a greater L/M-cone score compared with wild-type cones (Fig. 3H,J), suggesting that the *DIO3*^Δ*33*^ mutant cones are more L/M-cone-like. In *DIO3*^Δ*33*^ mutant cones, the *S-opsin* locus is less accessible and the *L-opsin* promoter is more accessible compared with wild-type cones (Fig. 3I,K), in line with the loss of S-opsin^+^ cells and generation of L/M^+^ cells in *DIO3*^Δ*33*^ mutants on day 200 (Fig. 2E,H,I). These data are consistent with the coexpression of L/M-opsin in S-opsin^+^ cells in *DIO3*^Δ*33*^ mutants.

Our analysis of *DIO3*^Δ*33*^ mutant organoids suggested that a subset of rods adopt features of L/M-cone fate. We observed a distinct population of cells (enriched in *DIO3*^Δ*33*^ mutants) that appeared transcriptionally intermediate between the rod and cone clusters (Supplemental Fig. S4F, bracketed). To characterize these cells, we applied our S-cone score (Supplemental Fig. S4O), L/M-cone score (Supplemental Fig. S4P), and rod score (Supplemental Fig. S4Q) to wild-type S cones (Supplemental Fig. S4Q, blue), L/M cones (Supplemental Fig. S4Q, yellow), and rods (Supplemental Fig. S4Q, red), as well as the putative hybrid cells from the *DIO3*^Δ*33*^ mutant organoids (Supplemental Fig. S4Q, orange). Each wild-type PR subtype was enriched for its respective score (Supplemental Fig. S4O–Q). In contrast, the *DIO3*^Δ*33*^ hybrid cells had low S-cone scores but high L/M-cone and rod scores (Supplemental Fig. S4O–Q), suggesting that these cells exhibit features of both L/M cones and rods.

We next applied a random forest classifier (trained on control rods, S cones, and L/M cones using the top 500 enriched genes per cell type) to predict the probabilities for the putative *DIO3*^Δ*33*^ hybrid PRs to have S, L/M, or rod identity. Most hybrid cells showed the highest predicted probabilities for L/M-cone identity, intermediate probabilities for rod identity, and minimal probabilities for S-cone identity (Supplemental Fig. S4R), suggesting that these cells predominantly adopt an L/M-cone-like transcriptional identity, with contributions from rod-specific gene expression.

Despite the small sample size (*n* = 60 *DIO3*^Δ*33*^ hybrid cells), these transcriptomic data support our opsin protein expression analysis (Fig. 2E–M; Supplemental Fig. S3M–T) and provide further evidence of nonexclusive photoreceptor fates in *DIO3*^Δ*33*^ mutants.

### DIO3 expression is regulated by feedback

We next sought to determine how DIO3 and TH signaling are regulated within and between retinal cells. On the organismal scale, TH levels are regulated by a negative feedback loop in which excess circulating T4 inhibits generation and release of TH from the thyroid gland (Gelfand et al. 1987; McNerney and Johnston 2021). Considering the coordination of development across retinal tissue, we hypothesized that TH regulation might involve additional cell-intrinsic or cell-extrinsic feedback mechanisms.

**Figure 4.**
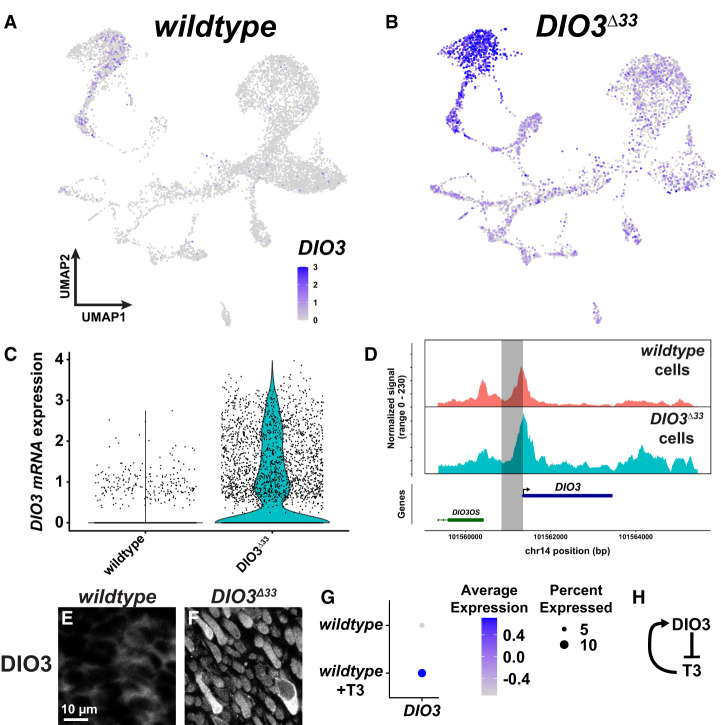
*DIO3* is controlled by feedback. (*A*,*B*) Expression of *DIO3* mRNA in wild-type (*A*) and *DIO3*^Δ*33*^ (*B*) organoids. (*C*) *DIO3* mRNA expression in retinal cells in wild-type and *DIO3*^Δ*33*^ organoids. (*D*, *top*) Accessibility of the *DIO3* locus in all retinal cells, split by genotype. (*Bottom*) Annotated gene locus. (*E*,*F*) DIO3 protein expression in wild-type (*E*) or *DIO3*^Δ*33*^ (*F*) organoids at day 180. The antigenic epitope is intact in *DIO3*^Δ*33*^ mutants, and the protein is detectable by IHC (Fig. 2A; Supplemental Fig. S1B,E). (*G*) *DIO3* mRNA expression in wild-type and wild-type +T3 conditions (Hussey et al. 2024). (*H*) Model of feedback network.

To assess feedback regulation, we analyzed our snRNA-seq data from organoids on day 200. In wild-type organoids, *DIO3* mRNA was expressed in RPCs and sparsely expressed in neurons (Fig. 4A), consistent with snRNA-seq analysis of human fetal retinal expression (Fig. 1A,B) and our assessment of *DIO3* expression in organoids (Fig. 1D–H). In *DIO3*^Δ*33*^ organoids, *DIO3* mRNA was upregulated, and the *DIO3* locus was more accessible across all cells (Fig. 4A–D) and in cones (Supplemental Fig. S5A,B) and rods (Supplemental Fig. S5C,D) specifically. Similarly, DIO3 protein was dramatically upregulated in *DIO3*^Δ*33*^ mutant organoids (Fig. 4E,F). These data suggest that *DIO3* gene expression is upregulated when DIO3 function is impaired.

Because DIO3 degrades TH and *DIO3* expression increases in *DIO3*^Δ*33*^ mutant organoids, we predicted that high T3 conditions would also lead to an increase in *DIO3* expression. To test this hypothesis, we analyzed our previously published scRNA-seq data generated from day 200 wild-type organoids and wild-type *+*T3 organoids (Hussey et al. 2024). *DIO3* expression increased in high T3 conditions (Fig. 4G), suggesting that TH signaling activates *DIO3* expression.

Together, these results suggest that DIO3 and TH interact in a feedback loop: DIO3 degrades TH, which in turn activates *DIO3* expression (Fig. 4H). This negative feedback mechanism is consistent with local homeostatic regulation of *DIO3* expression and TH signaling.

### DIO3 is regulated by non-cell-autonomous feedback

**Figure 5.**
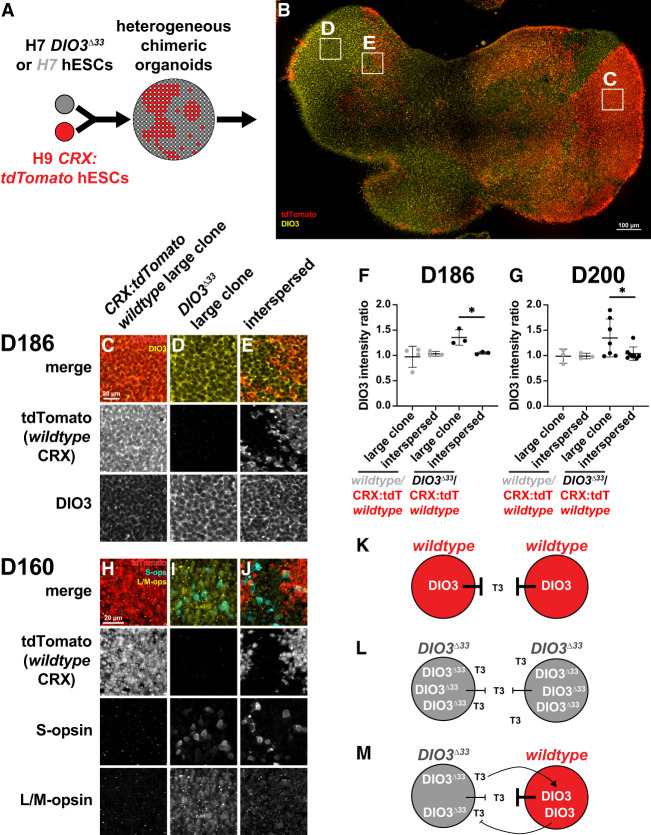
Chimeric organoids reveal non-cell-autonomous roles of DIO3. (*A*) Unlabeled H7 (control) or H7 *DIO3*^Δ*33*^ stem cells were mixed with H9 *CRX:tdTomato* reporter stem cells to generate chimeric organoids. (*B*) D186 representative chimeric retinal organoid. tdTomato^+^ regions are H9 wild-type *CRX:tdTomato* cells. tdTomato^−^ regions are H7 *DIO3*^Δ*33*^ cells. Boxed regions correspond to *C*–*E*. (*C*–*E*) Clones at D186. Large wild-type *CRX:tdTomato* clone (*C*), large *DIO3*^Δ*33*^ clone (*D*), and interspersed cells (*E*) stained for tdTomato and DIO3. (*F*) DIO3 intensity ratios for D186 clones. *N* = 3 wild-type/wild-type *CRX:tdTomato* chimeric organoids, *N* = 4 *DIO3*^Δ*33*^/wild-type *CRX:tdTomato* chimeric organoids, *N* = 4 large wild-type/wild-type *CRX:tdTomato* matched large clones, *N* = 3 wild-type/wild-type *CRX:tdTomato* matched interspersed regions, *N* = 3 *DIO3*^Δ*33*^*/*wild-type *CRX:tdTomato* matched large clones, *N* = 3 *DIO3*^Δ*33*^:wild-type matched interspersed regions. *n* = 50 cells per genotype per clone. Error bars represent SD. Statistics were determined by unpaired *t*-test. Unlabeled comparisons are not significant (*P* > 0.05). *DIO3*^Δ*33*^/wild-type *CRX:tdTomato* large clone versus interspersed ratio, *P* = 0.0267. (*G*) DIO3 intensity ratios for D200 clones. *N* = 5 wild-type/wild-type *CRX:tdTomato* chimeric organoids, *N* = 11 *DIO3*^Δ*33*^/wild-type *CRX:tdTomato* chimeric organoids, *N* = 3 large wild-type/wild-type *CRX:tdTomato* matched large clones, *N* = 3 wild-type/wild-type *CRX:tdTomato* matched interspersed regions, *N* = 7 *DIO3*^Δ*33*^/wild-type *CRX:tdTomato* matched large clones, *N* = 8 *DIO3*^Δ*33*^/wild-type *CRX:tdTomato* matched interspersed regions. *n* = 50 cells per genotype per clone. Error bars represent SD. Statistics were determined by unpaired *t*-test. Unlabeled comparisons are not significant (*P* > 0.05). *DIO3*^Δ*33*^/wild-type *CRX:tdTomato* large clone versus interspersed ratio, *P* = 0.0466. (*H*–*J*) Representative D160 chimeric organoids stained for S-opsin, L/M-opsin, and tdTomato reporter expression for *N* = 2 organoids, which contained all three clone types. (*H*) Large wild-type *CRX:tdTomato* region. (*I*) Large *DIO3*^Δ*33*^ region. (*J*) Boundary/interspersed region. (*K*–*M*) Model of non-cell-autonomous signaling in homogenous or large wild-type *CRX:tdTomato* regions (*K*), homogeneous or large *DIO3*^Δ*33*^ regions (*L*), and interspersed regions (*M*).

Because retinal development is coordinated and DIO3 regulates TH levels to control the timing of PR development, we hypothesized that DIO3 and TH signaling are regulated by intercellular feedback. To test this, we generated two sets of chimeric organoids. We used three types of stem cells: wild-type H7 stem cells, *DIO3*^Δ*33*^ mutant H7 stem cells, and *CRX:tdTomato* wild-type H9 stem cells. In the control, we mixed unmarked wild-type H7 cells with *CRX:tdTomato* wild-type H9 cells to account for potential differences in hESC line differentiation dynamics. In the experimental set, we mixed *DIO3*^Δ*33*^ H7 mutant cells with *CRX:tdTomato* wild-type H9 cells. From these mixes of stem cells, we differentiated the heterogeneous aggregates into chimeric retinal organoids (Fig. 5A,B).

In organoid tissue derived from the wild-type cells or *DIO3*^Δ*33*^ mutant cells, cells were unmarked. In organoid tissue derived from the *CRX:tdTomato* wild-type cells, wild-type PR precursors and PRs were marked by expression of the *CRX:tdTomato* reporter (Fig. 5A,B; Phillips et al. 2018). This strategy produced chimeric organoids with clones of varying size and location, enabling characterization of spatial effects based on differences in TH signaling regulation in wild-type and *DIO3*^Δ*33*^ mutant cells (Fig. 5B–E). We observed large clones of >1000 cells of a genotype (Fig. 5C,D) and interspersed cells in which each cell of a genotype was bordered by more than one cell of the other genotype (Fig. 5E).

At day 200, DIO3 expression was low in homogenous wild-type organoids and higher in homogenous *DIO3*^Δ*33*^ mutant organoids (Fig. 4E,F). Within a single organoid, large wild-type clones had low DIO3 expression levels, while large *DIO3*^Δ*33*^ clones had higher DIO3 levels (Fig. 5C,D), consistent with observations in homogenous organoids of either genotype (Fig. 4E,F) and suggesting that any non-cell-autonomous effects are distance-limited. In the interspersed cell population, both wild-type and *DIO3*^Δ*33*^ mutant cells had similar, intermediate levels of DIO3 (Fig. 5E). This result suggests that non-cell-autonomous regulation between cells coupled with feedback results in similar levels of DIO3 protein.

To quantify these differences, we measured DIO3 expression intensity in 50 cells of each genotype per clone type at days 186 and 200. To control for variability among organoids, we related expression levels to four ratios (Fig. 5F,G): (1) wild type*/CRX:tdTomato* wild type in large clones, (2) wild type*/CRX:tdTomato* wild type in interspersed clones, (3) *DIO3*^Δ*33*^*/CRX:tdTomato* wild type in large clones, and (4) *DIO3*^Δ*33*^*/CRX:tdTomato* wild type in interspersed clones. The first and second ratios were controls to address differences in wild-type DIO3 expression between stem cell lines and were expected to be ∼1. The third ratio was a positive control, and higher DIO3 expression in the *DIO3*^Δ*33*^ mutant was expected (i.e., >1). The fourth ratio, in combination with our controls, allowed for identification of non-cell-autonomous signaling effects. A ratio of ∼1 would indicate non-cell-autonomous signaling, whereas a ratio >1 would suggest cell-autonomous signaling. This approach enabled us to compare ratios of DIO3 expression intensity between organoids (Fig. 5C–G). For these experiments, we observed similar results on days 186 and 200 (Fig. 5F–G).

In control wild-type/wild-type *CRX:tdTomato* organoids, the ratios of DIO3 expression level intensity in both large and interspersed clones were ∼1 (Fig. 5F–G), suggesting that the levels of DIO3 were similar in both clone types and that the H7 and H9 hESC lines had similar DIO3 expression patterns. In contrast, large clones in the *DIO3*^Δ*33*^/wild-type *CRX:tdTomato* organoids had a greater DIO3 intensity ratio of ∼1.3 (Fig. 5F–G), consistent with high DIO3 expression in *DIO3*^Δ*33*^ mutant cells and lower DIO3 expression in wild-type *CRX:tdTomato* cells. Interspersed cells in the *DIO3*^Δ*33*^/wild-type *CRX:tdTomato* organoids had a DIO3 intensity ratio closer to ∼1 (Fig. 5F–G), consistent with non-cell-autonomous mechanisms yielding similar levels of DIO3.

We interpret these data as follows: In homogenous wild-type organoids or large wild-type clones, functional DIO3 degrades T3, and DIO3 levels are low (Fig. 5K). In homogenous *DIO3*^Δ*33*^ mutant organoids or large clones of *DIO3*^Δ*33*^, DIO3^Δ33^ protein cannot properly degrade T3, and the resulting high T3 environment induces DIO3 expression (Fig. 5L). Our observations of intermediate DIO3 levels in interspersed clones of *DIO3*^Δ*33*^ and wild-type cells suggest local intercellular feedback regulation. In interspersed *DIO3*^Δ*33*^ cells, impaired DIO3^Δ33^ protein yields high T3, which upregulates DIO3 in neighboring wild-type cells. Functional DIO3 in wild-type cells degrades T3 to lower T3 levels, leading to downregulation of DIO3 in the *DIO3*^Δ*33*^ cells (Fig. 5m). This intercellular feedback coordinates DIO3 expression across cells to yield intermediate levels.

### Cone developmental timing is controlled by non-cell-autonomous regulation

To determine whether non-cell-autonomous regulation affected PR development, we compared cone subtype development in large wild-type *CRX:tdTomato* clones, large *DIO3*^Δ*33*^ mutant clones, and interspersed cells on day 160 (Fig. 5H–J; Supplemental Fig. S6). Large wild-type clones had low photoreceptor density (Fig. 5H; Supplemental Fig. S6A, solid boxed region). Large *DIO3*^Δ*33*^ clones had greater S-opsin^+^ and L/M-opsin^+^ cell densities (Fig. 5I; Supplemental Fig. S6B, solid boxed region), consistent with accelerated cone development in *DIO3*^Δ*33*^ cells. Interspersed clones or clone boundaries had an intermediate amount of S-opsin^+^ and L/M-opsin^+^ cells (Fig. 5J; Supplemental Fig. S6C, solid boxed region). These data suggest that DIO3 expression and cone subtype developmental timing are regulated by TH levels and locally coordinated across cells.

### TH-mediated regulation of PR specification dictates developmental timing and fate

Our data suggest that the coupling of T3 degradation to cell differentiation (RPCs express DIO3, whereas differentiated neurons do not) implements a feedback loop that controls the timing of photoreceptor development. In this “signaling model,” cell ratios and intercellular signaling dynamically tune the rates of cell differentiation. This description is in contrast to the probabilistic, cell-intrinsic mechanism previously proposed to control neuronal subtype specification in the retina (intrinsic model) (Cepko 2014).

**Figure 6.**
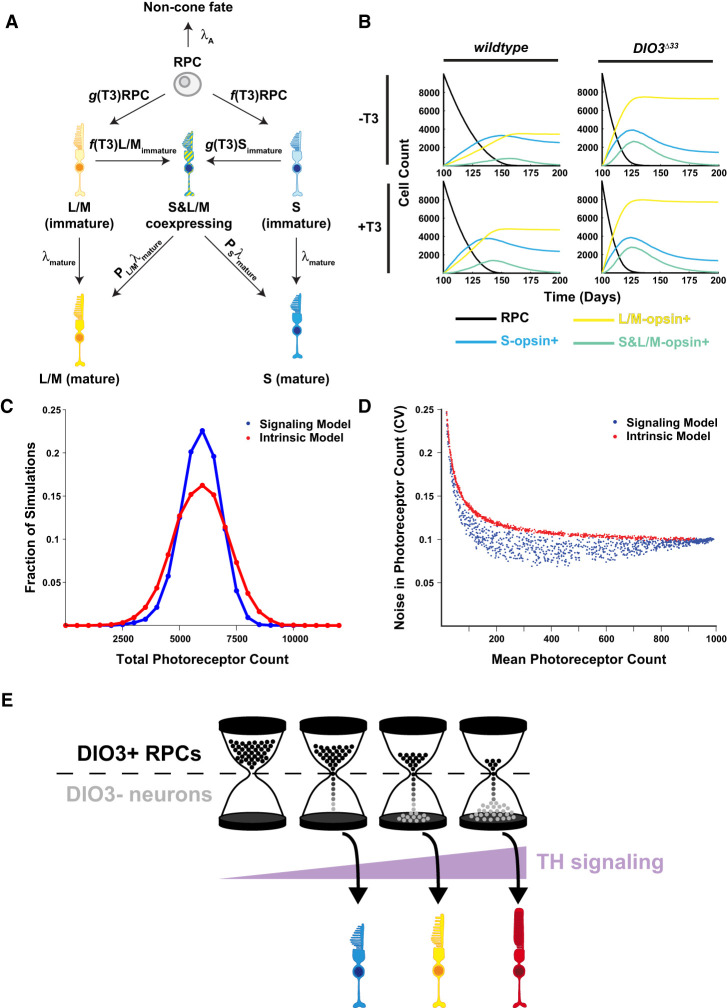
TH feedback confers robustness to photoreceptor specification. (*A*) Signaling model schematic. RPCs (gray) differentiate into immature L/M (yellow) and S (blue) cones with T3-dependent rates and into noncone fates with a constant rate. Functions *g* and *f* are selected as Hill functions with *n* = 1. Immature cones differentiate into mature cones with constant rates. Immature cones can express a second opsin gene to become S- and L/M-expressing cones (hashed yellow/blue). (*B*) Simulation of cone specification dynamics. Wild-type (*left*) and *DIO3*^Δ*33*^ (*right*) organoids in the absence (*top*) and presence (*bottom*) of exogenous T3 are shown. Curves depict counts of RPCs (black), L/M cones (yellow), S cones (blue), and S- and L/M-coexpressing cones (green). (*C*) T3 signaling filters noise in initial RPC abundance. Histograms show the total number of photoreceptors (S cones + L/M cones) specified in 5000 simulations seeded with a random number of initial RPCs in signaling (blue) and cell-intrinsic (red) models. (*D*) Exploration of model parameter space. Simulations (*n* = 1000) were performed for signaling (blue) and cell-intrinsic (red) models with randomly sampled parameter values. Each dot represents the performance of 50 replicate simulations with a given parameter set. (*E*) “Hourglass hypothesis.”

To identify potential functions of DIO3-mediated feedback, we formalized our observations into a stochastic model of photoreceptor development. In this model (Fig. 6A; Supplemental Material), RPCs differentiate into immature S cones or L/M cones with T3-dependent rates or differentiate into noncone fates with a constant rate. Immature cones mature into terminally differentiated cones with constant, T3-independent rates. Finally, S- and L/M-coexpressing cones arise from immature S or L/M cones via a “secondary firing” of the T3-dependent differentiation program before terminal maturation. T3 levels are regulated by the abundance of undifferentiated RPCs; each RPC expresses DIO3 and is assumed to degrade T3 with first-order kinetics. A detailed discussion of model rate functions, parameters, and assumptions is in the Supplemental Material.

Our model captures the key features of photoreceptor specification dynamics in wild-type organoids. Specifically, the model reproduces the ordering (S before L/M) of photoreceptor maturation, as well as the appearance of a small number of S-opsin- and L/M-opsin-coexpressing cells (Figs. 2F,G, 6B, top left). To simulate *DIO3*^Δ*33*^ organoid development, we altered the rate constant of DIO3-mediated T3 degradation to 6% of its wild-type value. This modified model correctly predicts the increase in L/M-cone:S-cone ratio and accelerated cone specification dynamics observed in *DIO3*^Δ*33*^ organoids (Figs. 2H,I, 6B, top right). Finally, the model recapitulates the observed phenotypes of wild-type +T3 (modest acceleration of differentiation) and *DIO3*^Δ*33*^ +T3 (no effect compared with *DIO3*^Δ*33*^) (Figs. 2J–M, 6B, bottom left and bottom right).

### The TH-dependent mechanism confers developmental robustness

We next used our model to explore whether T3 feedback offers advantages over a cell-intrinsic mechanism. Specifically, we tested whether this control enhances the robustness of photoreceptor specification. To this end, we performed a series of stochastic simulations in which each simulation is seeded with a random number of initial RPCs. We found that T3 signaling enables the system to “filter out” noise in RPC number (Fig. 6C); the distribution of final photoreceptors specified in the signaling model (Fig. 6C, blue) is narrower than that of a cell-intrinsic model (Fig. 6C, red) in which RPCs stochastically differentiate with constant rates.

Finally, to verify that this noise filtering is a general property of the signaling model rather than a product of specific parameter value choices, we simulated 1000 instantiations of the signaling and intrinsic models, each with randomly sampled parameter values (Fig. 6D). We found that the noise in final photoreceptor count (as measured by the coefficient of variation) is systematically lower for the signaling molecule (Fig. 6D, blue) than for the intrinsic model (Fig. 6D, red). Indeed, the cell-intrinsic model represents an upper bound for the poorest-performing versions of the signaling model. We also tested a modified version of this model in which DIO3 expression is maintained in immature cones and observed similar results (Supplemental Fig. S7A–D).

## Discussion

The mechanisms that control the timing of human retinal cell development have remained elusive. Our results suggest a link between the temporal regulation of TH signaling and the generation, maturation, and stability of PR subtypes. RPCs express DIO3, which diminishes as PRs and other retinal cells differentiate. As the RPC:neuron ratio decreases, TH degradation is relieved, leading to increased signaling and development of PR subtypes at distinct times. DIO3 and TH signaling levels are coordinated across cells by local intercellular feedback, providing robustness to cone subtype specification compared with probabilistic, intrinsic mechanisms. Here we discuss the implications of these findings for the specification of PR subtypes and more generally for the strategies that coordinate cell type generation and development in the retina and other contexts.

### DIO3 and TH signaling regulate PR number, developmental timing, and fate stability

The dynamic expression and function of DIO3 are essential for regulating PR development. DIO3 is highly expressed in RPCs and decreases as these cells differentiate into neurons and mature. During retinal development, the asynchronous differentiation of RPCs leads to a progressive reduction in overall DIO3 levels. This reduction establishes a temporal gradient of TH signaling, which provides critical timing cues for PR development.

This temporal gradient mechanism can be regarded as a twist on the “French flag” model of spatial embryonic development, first proposed by Wolpert (1969). In this model, specification is determined by position along a spatial gradient of a morphogen, later shown to be the transcription factor Bicoid in fly embryos (Driever and Nüsslein-Volhard 1988). In the retina, the gradual accumulation of T3 “patterns” PR development in time rather than in space.

DIO3 is required to limit PR generation, set PR developmental timing, and ensure PR fate stability. In *DIO3*^Δ*33*^ mutants, S-opsin, L/M-opsin, and Rho are expressed earlier and in greater numbers of cells. Later, subsets of S-opsin^+^ and Rho^+^ cells also express L/M-opsin, indicating a loss of cell fate exclusivity and a possible transformation in PR subtype identity.

In high TH conditions (i.e., *DIO3*^Δ*33*^ and *+*T3), PRs that coexpress different opsins could ultimately regress back to their initial cell fate, maintain a hybrid state, convert to a new cell fate, or die. The observed opsin coexpression is likely due to sustained expression of both opsins and/or perdurance of the opsin protein that was initially expressed. Previously, we observed evidence of cell fate instability when we analyzed S-cone and L/M-cone fates via scRNA-seq upon perturbation of TH signaling conditions (Hussey et al. 2024). In organoids exposed to a short 10 day pulse of T3 after the generation of S cones, the L/M-cone population remained stable, whereas the S-cone population decreased and the unclassified cone population increased, suggesting a destabilization of S-cone fate, likely leading to their transformation into L/M cones.

We observed similar PR number, developmental timing, and fate stability phenotypes in *DIO3*^Δ*33*^, wild-type +T3*,* and *DIO3*^Δ*33*^ *+*T3 organoids. The milder phenotype in wild-type *+*T3 organoids is consistent with the presence of wild-type DIO3, which is upregulated in high T3 to increase degradation and maintain T3 levels. The highly similar phenotypes of *DIO3*^Δ*33*^ mutant organoids in the presence or absence of additional T3 suggest that DIO3 and TH signaling act in the same pathway and that T3 levels in the media are saturating.

While a great focus has been placed on terminal cell fates, the temporality of cell fate decisions is less understood. *DIO3*^Δ*33*^ mutant and wild-type +T3 organoids showed an advancement in S-opsin, L/M-opsin, and rhodopsin expression (Fig. 2; Supplemental Fig. S2). This suggests that excess TH signaling accelerates cell fate specification. This could be due to either an advancement of cell cycle exit or a preference toward differentiating photoreceptors from the RPC pool. TH has previously been shown to influence cell cycle (Durand and Raff 2000). In oligodendrocyte precursor cells, TH signaling promotes differentiation and is hypothesized to be part of a timing mechanism in which cells count how many cell cycles occur before terminal differentiation (Durand and Raff 2000; Alisi et al. 2004; Porlan et al. 2004). If a similar mechanism is occurring in PR precursors, the excess TH in wild type +T3 and in *DIO3*^Δ*33*^ might be inducing cell cycle exit prematurely, causing the advancement of opsin^+^ cells.

In addition to cone development, DIO3 and TH signaling regulate rod development. Our findings in human organoids are consistent with studies in chickens, where intraocular injections of T3 promoted early PR formation and differentiation (Fischer et al. 2011). Rod development is also regulated by retinoic acid (RA), another nuclear hormone signaling mechanism. RA promotes rod specification in human retinal organoids, mice, and zebrafish (Kelley et al. 1999; Prabhudesai et al. 2005; Khanna et al. 2006; Oh et al. 2008). Our data suggest that TH works alongside RA to control the timing of rod development. RA receptors and TH receptors can heterodimerize (Zhang et al. 1992; Butler and Parker 1995; Koenig 1998; Roberts et al. 2006), and we hypothesize that modulation of both RA and TH signaling is important for developmental timing and subtype specification of PRs.

We previously showed that RA promotes early human cone subtype fates, specifying S-cone fate over L/M-cone fate and M-cone fate over L-cone fate (Hadyniak et al. 2024; Hussey et al. 2024). Consistent with these roles for RA signaling, aldehyde dehydrogenases (ALDHs), enzymes that generate RA, are highly expressed early in human retinal development (Hoshino et al. 2017; Hadyniak et al. 2024). Both RA-generating ALDHs and TH-degrading DIO3 are highly expressed early and decrease during development, suggesting that high RA signaling early transitions to high TH signaling late, leading to temporally inverse gradients of signaling. These temporal countergradients are conceptually similar to the spatial countergradients of Shh and BMP/Wnt that pattern the neurons of the spinal cord (Ericson et al. 1997a,b; Briscoe and Ericson 1999; Briscoe et al. 2000, 2001; Jessell 2000; Patten and Placzek 2002; Stamataki et al. 2005; Fuccillo et al. 2006; Dessaud et al. 2010; Exelby et al. 2021; Douceau et al. 2023). Because both TH and RA act through a diverse set of nuclear hormone receptors to activate and repress gene expression (Aranda and Pascual 2001; Astapova and Hollenberg 2013; Cho et al. 2023), these countergradients may work together to trigger temporal events during retinal development.

Beyond the retina, the temporal regulation of TH signaling plays important roles in the development of several other tissues. During development of the cochlea in mice, TH is limited early and increases before the onset of hearing (Campos-Barros et al. 1999; Ng et al. 2004, 2009). In the brain, a similar transition from low to high TH signaling regulates differentiation of oligodendrocytes (Barres et al. 1994; Ahlgren et al. 1997; Guadaño-Ferraz et al. 1997; Tu et al. 1997, 1999; Carre et al. 1998; Gao et al. 1998; Rodríguez-Peña 1999; Billon et al. 2001; Calza et al. 2002; Courtin et al. 2005; Lee and Petratos 2016). Our findings in the retina may be a paradigm for understanding the mechanisms controlling the temporality of TH signaling and development in other contexts.

### Comparing roles for DIO3 in human and mouse PR development

Our mechanistic studies of DIO3 reveal similarities and differences in its role in PR development in human retinal organoids and mouse retinas. In mouse retinas, human retinas, and human retinal organoids, *DIO3* is highly expressed early and gradually decreases over time (Fig. 1; Ng et al. 2010, 2017; Eldred et al. 2018). *Dio3*Δ mutant mice display cone loss (Roberts et al. 2006; Lu et al. 2009; Ng et al. 2009, 2010, 2011, 2017; Ma et al. 2014 ). Similarly, *DIO3*-null mutant organoids fail to differentiate retinal tissue (Supplemental Fig. S1E–H). In contrast, *DIO3*^Δ*33*^ mutant organoids differentiate retinal tissue with accelerated PR development (Figs. 2, 3).

These differences partly stem from the in vivo versus in vitro conditions. In mice, TH signaling is influenced by circulating TH levels and interactions with neighboring tissues. In contrast, organoids are exposed to a consistent TH supply provided by the media, meaning any changes in TH signaling must originate from retinal organoid tissue-derived mechanisms.

Additionally, the nature of the mutant alleles contributes to the phenotypic differences. Null mutants lead to loss of retinal cells or failure to differentiate retinal tissue. In contrast, *DIO3*^Δ*33*^ mutant organoids, which exhibit a significant yet incomplete loss of function, successfully differentiate retinal tissue, revealing new roles for DIO3-mediated regulation of PR development.

### Differentiation-dependent control of neuronal subtype developmental timing

Our data suggest that the ratio of progenitors and neurons controls PR developmental timing. As this ratio decreases, the proportion of cells expressing DIO3 decreases, leading to increased TH signaling. This mechanism shares some similarities with the regulation of retinal ganglion cell (RGC) generation. RGCs are the first neurons to develop in the retina. Studies in mice have shown that RCGs express Sonic Hedgehog (Shh), which signals to RPCs to inhibit RGC production (Zhang and Yang 2001; Stenkamp and Frey 2003; Wang et al. 2005). This mechanism provides a shutoff switch for RGC production. As the population of Shh-expressing RGCs increases, RPC differentiation into RGCs is stopped, allowing the differentiation of other cell types to progress.

Similar to the relationship between Shh signaling and RGC development, our study identified a signaling regulatory mechanism linked to progenitor differentiation. For RGCs, differentiation leads to increased Shh signaling, which in turn inhibits further RGC differentiation. For PRs, differentiation leads to reduced DIO3 expression and activity, increasing TH signaling to promote PR development and terminal L/M-cone fate.

Other tissues also use differentiation-regulated signaling to temporally drive fate choices. In the spinal cord, early-born medial lateral motor column (LMC) neurons express RALDH2, which produces RA, which induces late-born LMC to adopt a lateral LMC fate (Sockanathan and Jessell 1998). Thus, early-born neurons express a signal that specifies late-born neurons. In contrast, in the retina, the generation of neurons relieves degradation of a signal that drives PR development.

### Signaling and feedback promote robustness of photoreceptor developmental timing

TH signaling is regulated by negative feedback to maintain homeostatic levels on the organismal scale. The HPT axis responds to extremes in TH levels by modulating TSH and TRH release from the hypothalamus and pituitary, respectively, to control TH synthesis and release. Here, we present evidence for local intercellular feedback and coordination of TH regulation in retinal development. The decreasing ratio of progenitors and neurons controls the levels of TH signaling to regulate the temporality of PR development. Signaling is coordinated by feedback between and within retinal cells, which are sensitive to TH levels and respond by modulating expression of TH regulators.

Our experimental results and modeling suggest that coupling TH signaling to photoreceptor differentiation may confer robustness to retinal development (Fig. 6C,D). The signaling model achieves tight timing of the duration of photoreceptor specification (Fig. 6B–D)—a potentially useful property for coordinating the development of two independent eyes. As we explore in the Supplemental Material, the principles underlying this noise control are also used to achieve precise control in biofilm development (Norman et al. 2013; Lord et al. 2019) and synthetic oscillators (Potvin-Trottier et al. 2016).

Whereas our findings identify new roles for signaling in the temporal regulation of retinal PR development, these extrinsic mechanisms must work together with intrinsic mechanisms involving chromatin and transcriptional regulation to differentiate retinal cell types (Emerson et al. 2013; La Torre et al. 2013; Cepko 2014; Wang et al. 2014; Andzelm et al. 2015; Schick et al. 2019).

### The hourglass hypothesis for regulation of photoreceptor developmental timing

Our data suggest that the changing ratio of RPCs to neurons regulates DIO3 levels, which in turn control T3 levels to determine the timing of PR development. To conceptualize this mechanism, we propose an “hourglass hypothesis” (Supplemental Fig. 6E). In this analogy, the sand at the top of the hourglass represents progenitors, and the sand at the bottom represents differentiated retinal cells. Initially, the hourglass is full at the top, meaning the retina is composed primarily of progenitors. These progenitors express DIO3, maintaining low TH signaling. As development progresses, the sand in the hourglass trickles downward as the progenitors gradually differentiate. This shift reduces the progenitor to differentiated cell ratio, decreasing the proportion of cells that express DIO3 and increasing TH signaling. As TH levels reach critical thresholds, PR subtypes are generated and develop at specific times. Eventually, all the sand reaches the bottom, signifying the depletion of the progenitor pool and the complete generation of all retinal neurons and Müller glia.

Studies of the rat retina revealed a relatively consistent shift in the ratio of RPCs to postmitotic cells during development (Alexiades and Cepko 1996). To investigate whether a similar progression occurs in humans, we calculated the ratio of RPCs to postmitotic cells from a published snRNA-seq data set from developing human fetal retinas (https://explore.data.humancellatlas.org/projects/581de139-461f-4875-b408-56453a9082c7). Our analysis uncovered a similar progressive shift in the RPC to postmitotic cell ratio over time (Supplemental Fig. S7E). This finding is striking, given the genetic differences and technical variability inherent in studying human fetal tissues compared with the retinas of inbred mice raised under controlled conditions. Although some deviations from this steady progression very likely occur, the overall trajectory suggests a consistent developmental shift.

The human retina contains distinct regions of specialized PR patterning, including the foveola, fovea, macula, periphery, and rim (for review, see Hussey et al. 2022). We hypothesize that these regional patterns arise from modulation of TH and retinoic acid signaling by differences in the location and timing of expression of regulators. The coexpression of L/M-opsin in S-opsin^+^ cells in organoids grown in high TH conditions mirrors our previous observations in the developing foveola (Hussey et al. 2024), the central region of the retina responsible for high-acuity vision. The presumptive foveola contains S-opsin^+^ cells during early fetal development, gains coexpression of L/M-opsin in S-opsin^+^ cells later, and resolves to L/M cones only in adults (Xiao and Hendrickson 2000; Cornish et al. 2004a; Hussey et al. 2024). These changes in opsin expression, indicative of a cell fate progression, are associated with localized, sustained high expression of DIO2, the enzyme that generates T3 and promotes TH signaling. The transient coexpression of L/M-opsin in S-opsin^+^ cells, followed by the loss of S-opsin^+^ cells (Hussey et al. 2024), is consistent with a cell fate transformation. Although death of foveolar PRs has not been observed, it remains possible that these cells are eliminated by apoptosis. Together, the sustained expression of DIO2 and coexpression of S-opsins and L/M-opsins in the fetal foveola are consistent with the modulation of the hourglass mechanism observed under high TH conditions. We propose that the sustained expression of DIO2 in the foveola maintains elevated TH signaling, driving the high density of L/M cones in the adult foveola.

Despite these complexities, the hourglass hypothesis provides a valuable, new conceptual framework for investigating how the ratio of RPCs and neurons controls extrinsic signaling to influence cell fate specification and maturation during retinal development.

### Implications for retinal therapies

Our work has identified how cell population-based signaling coordinates developmental timing in the retina. These studies not only deepen our understanding of cell fate specification and human retinal development but also hold potential implications for translational medicine.

Globally, thyroid disorders affect hundreds of millions of people (Zhang et al. 2023b). As TH affects many aspects of development, multiple layers of regulation protect the fetus from fluctuations in maternal TH and fetal TH as the fetal HPT develops. This complexity makes it difficult to interrogate the crucial role of TH in retinal development in a complex organism. A strength of our retinal organoid experiments is the ability to isolate the developing retinal tissue from systemic TH regulation. This enabled us to examine the role of TH regulation in retinal development without complication from the HPT axis. Similarly, human retinal organoids have been used to identify mechanisms of retinal development and model degeneration (Phillips et al. 2014; Fligor et al. 2018; Kallman et al. 2020; Lu et al. 2020; O'Hara-Wright and Gonzalez-Cordero 2020; Sridhar et al. 2020; Cuevas et al. 2021; Guy et al. 2021; Kandoi et al. 2024).

Technological advances in organoids combined with deeper understanding of developmental processes can advance cell and gene therapies. TH dysregulation has been suggested to have a role in several retinal diseases including diabetic retinopathy and age-related macular degeneration (Nicolini et al. 2024), and further study may reveal new targets for therapeutics. Additionally, retinal organoids can be used as a source of transplantable, healthy retinal cells (Shirai et al. 2016; Singh et al. 2019; Zou et al. 2019; Li et al. 2021; Gasparini et al. 2022; Watari et al. 2023; Iwama et al. 2024). An understanding of mechanisms of cell fate specification can be leveraged to generate organoids with cell constituencies tailored to regions of the retina such as the fovea, which is enriched in L/M cones (Diaz-Araya and Provis 1992; Bumsted and Hendrickson 1999; Cornish et al. 2004a,b; Hussey et al. 2024).

## Materials and methods

### Cell line maintenance

H7 ESCs (WiCell WA07), H9 ESCs (WiCell WA09) modified with CRX>tdTomato (gift from David Gamm [Phillips et al. 2018]), or IMR90-GFP iPSCs (Wahlin et al. 2021) were used for differentiation. Cells were maintained in mTeSR1 (Stem Cell Technologies 85850) on 1% (v/v) Matrigel-coated (Corning 354263) or Geltrex-coated (Thermo Fisher A1413202) dishes and grown at 37°C in a HERAcell 150i or 160i hypoxic incubator (10% CO_2_, 5% O_2_). Cells were passaged approximately every 5 days as described previously by Eldred et al. (2018). Cells were passaged with Accutase (Stem Cell Technologies 07922) for 7–10 min and dissociated to single cells through additional manual trituration. Cells suspended in Accutase were added 1:2 to a solution of mTeSR1 with 5 µM blebbistatin (MilliporeSigma B0560-5MG). Cells were pelleted at 300*g* for 5 min before being resuspended in mTeSR1 with 5 µM blebbistatin. Cells were plated on a new Matrigel-GFR-coated or Geltrex-coated 6 well plate at ∼5000–40,000 cells/well. Media was replaced with mTeSR1 48 h after passaging and every day until passaged again. To minimize cell stress, no antibiotics were used.

### Cell culture media

Cell culture media used in this study were as follows: stem cell media (mTeSR1), E6 supplement (970 µg/mL insulin [Roche 11376497001], 535 µg/mL holotransferrin [Sigma T0665], 3.20 mg/mL L-ascorbic acid [Sigma A8960], 0.7 µg/mL sodium selenite [Sigma S5261]), BE6.2 media (2.5% E6 supplement, 2% 50× B27 supplement minus vitamin A [Gibco 12587010], 1% GlutaMAX [Gibco 35050061], 1% NEAA [Gibco 11140050], 1 mM sodium pyruvate [Gibco 11360070], 0.87 mg/mL NaCl in DMEM [Gibco 11885084]), long-term retina (LTR) media (25% F12 [Gibco 11765062], 2% 50× B27 supplement [Gibco 17504044], 10% heat-inactivated FBS [Gibco 16140071], 1 mM sodium pyruvate [Gibco 11360070], 1% NEAA [Gibco 11140050], 1% GlutaMAX [Gibco 35050061], 1 mM taurine [Sigma T-8691] in DMEM [Gibco 11885084]), neural induction medium (NIM; 1% N2 supplement [Thermo Fisher 17502048], 1% MEM NEAA, 2 µg/mL heparin [Sigma-Aldrich H3149] in DMEM/F12 with GlutaMAX supplement [Gibco 10565018]), 3:1 medium (24% F12 with GlutaMAX [Gibco 31765035], 2% B27 supplement minus vitamin A, 1% MEM NEAA, 1% penicillin–streptomycin in DMEM with high glucose, GlutaMAX, pyruvate [Gibco 10569010]), 3:1* medium (22% F12 with GlutaMAX, 2% B27 supplement minus vitamin A, 1% MEM NEAA, 1% penicillin–streptomycin, 10% heat-inactivated FBS, 100 µM taurine in DMEM with high glucose, GlutaMAX, pyruvate), and N2 medium (24% F12 with GlutaMAX, 1% N2 supplement, 1% MEM NEAA, 1% penicillin–streptomycin, 10% heat-inactivated FBS, 100 µM taurine in DMEM with high glucose, GlutaMAX, pyruvate). Treatments used in this study were as follows: For retinoic acid treatment, stocks of 10.4 mM retinoic acid (ATRA; Sigma R2625) were made in DMSO. Retinoic acid was added to media for final concentrations of 1.04 or 0.52 µM. For thyroid hormone treatment, 20 nM T3 (Sigma T6397) in LTR media was as used previously in mouse retinal explant culture (Roberts et al. 2006) and human retinal organoids (Eldred et al. 2018). In +T3 conditions, T3 was added from day 42 until collection for analysis.

### Organoid differentiation

Organoids were differentiated from H7 WA07 ESCs, H9 WA09 ESCs, or IMR90 Six6>GFP iPSCs (Supplemental Fig. S8). hPSC cultures were monitored for spontaneous differentiation, and only well-maintained cultures were used for differentiation. Organoids were regularly monitored for health and were culled if retinal tissue was not apparent.

### Gravity aggregation (GA)

GA differentiation proceeded as described prevoiously by Wahlin et al. (2017), Eldred et al. (2018), and Hussey et al. (2024) (Supplemental Fig. S8A). Cells were passaged in Accutase for 10–12 min at 37°C and triturated to ensure single-cell dissociation. Cells were seeded in 50 µL of mTeSR1 with 5 µM blebbistatin at 3000 cells per well in 96 well ultra-low-adhesion round-bottom lipidure-coated plates (Corning 7007). Cells were placed in hypoxia (10% CO_2_, 5% O_2_) for 24 h at 37°C, during which time cells aggregated by gravity.

After 24 h and for the remainder of the differentiation, aggregates were grown in normoxic conditions (5% CO_2_) at 37°C. On days 1–3, 50 µL of Be6.2 media with 3 µM Wnt inhibitor (EMD Millipore 681669) and 1% (v/v) Matrigel was added to each well. On days 4–9, 100 µL of media was removed from each well and replaced with 100 µL of fresh media. On days 4–5, Be6.2 media with 3 µM Wnt inhibitor and 1% (v/v) Matrigel was added, and on days 6–7, Be6.2 media with 1% (v/v) Matrigel was added. On days 8–9, media was replaced with Be6.2 with 1% Matrigel and 100 nM smoothened agonist (SAG; Sigma-Aldrich 566660-1MG).

On day 10, aggregates were rinsed two to three times in DMEM and transferred to 10 cm Petri dishes in Be6.2 in 100 nM SAG. For the remainder of the differentiation, the media was changed every other day. On days 13–16, aggregates were transferred to LTR media with 100 nM SAG.

Between days 11 and 16, aggregates were dissected with tungsten needles as needed to isolate optic vesicles. To remove dead cells, aggregates were washed two to three times in DMEM as needed before feedings between days 16 and 50.

On days 16–20, aggregates were maintained in LTR media. From day 20–130, 1 µM all-*trans* retinoic acid (ATRA) was added to LTR media. On days 28–42, aggregates were additionally supplemented with 10 µM γ secretase inhibitor (DAPT; CalBiochem 565770-10 mg). Organoids were maintained in unsupplemented LTR media from day 131 to 200.

### AMASS

AMASS differentiation was described previously by Cowan et al. (2020) (Supplemental Fig. S8B). AggreWell 800 plates (Stem Cell Technologies 34811) were prepared on the day of differentiation according to the manufacturer's directions. Wells were washed once with mTeSR1. ESCs were dissociated into single cells in Accutase with additional manual trituration. ESCs were resuspended to the desired density in 0.5 mL, and this suspension was added to 1 mL of mTeSR1 + 5 µM blebbistatin per well of the microwell plate and returned to hypoxia at 37°C. To generate chimeras, the desired percentage of each genotype was calculated, and appropriate amounts of cells were added to reach a consistent total density of cells in one 0.5 mL well.

On day 1, aggregates received a one-third media exchange with NIM. On day 2, they received a one-half media replacement with NIM. On days 3–6, they received a full media exchange with NIM. On day 7, aggregates were transferred in NIM to a Geltrex-coated 6 well plate.

On days 8–15, the adherent aggregates were fed daily with full media replacement with NIM. On days 16–27, they were fed with 3:1 media.

Plates were scraped on day 28. Aggregates were washed and transferred to 10 cm Petri dishes in 3:1 media. From day 28 until the end of the differentiation, organoids were fed three times per week in 10 cm Petri dishes.

Organoids were fed with 3:1 media from day 28 to 41, 3:1* media from day 42 to97, and N2 media from day 98 until the end of differentiation. Media was supplemented with 1 µM RA from day 70 to 97 and 0.5 µM RA from day 98 until the end of differentiation. +T3 conditions were treated with T3 starting at day 42 and persisted until collection.

### Mycoplasma monitoring

Cell lines and organoid media were tested monthly for mycoplasma using MycoAlert (Lonza LT07) and excluded if positive.

### CRISPR/Cas9 mutations

CRISPR was performed as described previously by Eldred et al. (2018) and Hussey et al. (2024). Cloning gRNA plasmids and gRNA transfection plasmids were created using pSpCas9(BB)-P2A-Puro plasmid modified from the pX459_V2.0 plasmid (Addgene 62988) by replacing T2A with P2A. gRNAs were cloned into the modified vector following the Zhang laboratory's protocol (https://pharm.ucsf.edu/sites/pharm.ucsf.edu/files/xinchen/media-browser/CRISPR%20cloning%20protocol%20Zhang%20Lab.pdf).

Primers and primer sequences used in this study were as follows: DIO3 gRNA3 forward (DIO3Δ33; CACCGGGTTCCCTTGCGCGTAGTCG), DIO3 gRNA3 reverse (*DIO3*^Δ*33*^; AAACCGACTACGCGCAAGGGAACCC), DIO3 gRNA2 forward (*DIO3*^Δ^; CACCGCTCATGCGCGCCATGAACGG), and DIO3 gRNA2 forward (*DIO3*^Δ^; AAACCCGTTCATGGCGCGCATGAGC).

For transfection, selection, and genotyping, H7 ESCs were passaged to single cells, seeded at a density of 50,000 cells/well in mTeSR1 with 5 µM blebbistatin in a prepared Geltrex- or Matrigel-coated 24 well plate, and left in hypoxia at 37°C. Twenty-four hours after seeding, media was replaced with mTeSR1. The transfection mix was prepared by combining 200 ng of either modified pX459 plasmid (containing gRNA) or pX458 plasmid (Cas9>GFP-positive control), 50 µL of Optimem (Gibco 31985070), and 2 µL of Lipofectamine stem reagent (Invitrogen STEM00003) prepared according to the manufacturer's recommendations. Each transfection well received 50 µL of transfection mix.

Twenty-four hours later, media was replaced with mTeSR1. A gradient of puromycin concentrations ranging from 0.1 to 1.5 µg was applied to the plate. After 24 h, cells were washed once with mTeSR1 and then fed with fresh mTeSR1. At this time, transfection efficiency was estimated by observing GFP^+^ colonies in the Cas9>GFP transfected control cells. After several days, the condition in which the gRNA transfected cells survived puromycin treatment but the control Cas9>GFP transfected cells did not was passaged and expanded into a new Geltrex-coated 6 well plate. Individual colonies were manually isolated and split for either genotyping or maintenance in a new Geltrex-coated 48 well plate. DNA was extracted using QE buffer (Lucigen QE0905T), and PCR was used to amplify the relevant region of the gene. Mutations were identified by Sanger sequencing.

Primers and sequences used in this study were as follows: DIO3_seq_F_03 (TCTGGCTTCTCGATTTCTTGTG), DIO3_seq_F_04 (GTGTATCCGCAAGCATTTCCTG), DIO3_seq_R_01 (CACCACGGACTCTCCCTACATC), DIO3_seq_R_02 (TTCGAGCGTCTCTATGTCATCC), and DIO3_sequencing (GCTCAACAGTGAAGGCGAGG).

### Immunohistochemistry

Retinal organoids were fixed in freshly prepared 4% formaldehyde in PBS for 50 min to 1.25 h at 4°C. Organoids were washed three times in 5% sucrose in PBS and then transitioned through a sucrose gradient of 6.25%, 12.5%, and 25% sucrose in PBS. All sucrose incubations were performed for 20 min to 1 h (with the exception of the 25% sucrose, which was incubated overnight) at 4°C.

For whole mounts, organoids were transferred from 25% sucrose to block solution (2%–5% donkey serum, 0.2%–0.3% TritonX-100 in PBS) and incubated for 2–120 h at 4°C. Organoids were incubated in primary antibodies diluted in block for 16–72 h at 4°C. Organoids were washed three times for >15 min at room temperature in PBS and then incubated in secondary antibodies diluted in block solution for 4–48 h at 4°C. Organoids were washed with three >10 min incubations in PBS at room temperature. If organoids were stained with Hoechst, they were incubated for 10 min in Hoechst 33342 (Biotium 40046 10H0212) diluted 1:2000 in PBS at room temperature, followed by three PBS washes. Organoids were mounted in SlowFade.

For cryosections, following the sucrose gradient, organoids were flash-frozen in OCT and stored long term at −80°C. Organoids were cryosectioned at 10–12 µm. Sections were left to dry for 6 h to overnight at room temperature and then stored long term at −80°C. Sections were allowed to come to room temperature and then rehydrated for 10 min in PBS. Slides were incubated for 15–30 min at 60°C before proceeding with IHC. Block, primary, and secondary incubations were performed as described for whole-mount organoids.

For fetal tissues, 14 week old fetal eyes (male, left/right eye information unknown) were enucleated within 24 h of termination, flash-frozen in liquid nitrogen, and then stored at −80°C for downstream processing. Fetal eyes were briefly thawed in room temperature 1× PBS, transferred to 10% NBF, and fixed overnight at 4°C. The retina was dissected from the whole globe and transferred to block solution. IHC was performed as described for organoid whole-mount tissue.

Antibodies used in this study were as follows: goat ARR3 (1:200; Novus Biologicals NBP1-37003), mouse CRX (1:500; Abnova UJOHO013), mouse CRX (1:200; Sigma-Aldrich WH0001406M2), rabbit DIO3 (1:200; Novus Biologicals NBP1-05767), mouse Ki67 (1:200; Santa Cruz Biotechnology sc-23900), goat NRL (1:200; Bio-Techne AF2945), rabbit OPN1LW/OPN1MW (1:200; Sigma-Aldrich AB5405), chick OPN1LW/OPN1MW (1:200; kind gift from Jeremy Nathans), goat OPN1SW (1:200; Santa Cruz Biotechnology sc-14363), chick OPN1SW (1:200; kind gift from Jeremy Nathans), rabbit RCVRN (1:200; Sigma-Aldrich AB5585), rat RFP (1:400; Proteintech 5F8-150), mouse rhodopsin (1:500; GeneTex GTX23267), mouse rhodopsin (1:500; Thermo Fisher Scientific MA5-11741), and sheep VSX2 (1:100; Exalpha Biologicals X1180P). All secondary antibodies were raised in donkey, Alexa fluor-conjugated (Invitrogen), and used at 1:400 dilution.

### Microscopy and image processing

Bright-field images were acquired with either a Zeiss LSM 980 inverted microscope with an Axiocam 506 color camera system and a C Apo 40×/1.2 W DICIII lens or an EVOS XL core cell imaging system on DIC with a 4×, 10×, or 20× air objective lens (Thermo Fisher).

Fluorescent images were acquired using either a Zeiss LSM 800 or 980 laser scanning confocal microscope or a Nikon CSU-W1 SoRa spinning disk system. Confocal images were acquired with similar settings for laser power, photomultiplier gain and offset, and pinhole diameter, and camera images were acquired at similar epifluorescence intensity and exposure. Cones were imaged in 30–100 optical sections with 1.26 µm step size for confocal microscope or 0.50 µm for camera and visualized with a maximum intensity projection or a single *Z* section. For quantitative measurements of signal intensity, tissues were imaged at the same imaging parameters in the same imaging session to limit variability.

### Measurements and quantification

Manual quantification of photoreceptors was performed with Adobe Photoshop, and density was calculated using ImageJ. Semiautomated quantification of photoreceptors was done using the Object Colocalization FL v2.1.4 module of HALO image analysis platform 3.5.3577 (Indica Labs), which was used to count the number of S-opsin-, L/M-opsin-, and rhodopsin-expressing cells. Outer segment length was quantified using FIJI in a single *Z* that captured the focal plane of the OS. DIO3 fluorescence intensity was analyzed in FIJI using a single *Z* that captured the center focal plane of the cells being analyzed. Fifty cells were selected per clone type per organoid that were in-plane and CRX^+^. An appropriate radius was selected to encircle each cell, and the integrated density and standard deviation of the population were recorded.

GraphPad Prism was used for analysis and statistics. Statistical tests and *N* are listed in the figure legends. All error bars represent SEM. Organoids with <150 cones after day 120 were removed from analysis and assumed to not have properly differentiated.

### DIO3 enzymatic activity assay

Type 3 deiodinase activity was determined as described previously (Hernandez et al. 2006). In brief, organoids were homogenized in 100 µL of 10 mM Tris/0.25 m sucrose (pH 7.4) in the presence of 2 mM dithiothreitol (DTT). Ten microliters of the homogenate was assayed for protein content. For the enzymatic assay, 5 µL of the organoid homogenate was incubated with 100 fmol of radiolabeled T3 for 0.5 h at 37.5°C. The incubation mix contained 20 mM DTT and 1 mM propylthiouracil in a total volume of 50 µL. The reaction was stopped by adding 10 µL of 5 µM T3 and 50 µL of ethanol. An aliquot of the reaction was submitted to paper chromatography as described previously (Hernandez et al. 2006), and radiolabeled T3 and T2 (3,3′-diiodothyronine) were quantified in a γ counter to calculate the proportion of T3 deiodinated and enzymatic activity.

### Nucleus lysis

Nuclei were isolated from retinal organoids using a previously described lysis protocol (Santiago et al. 2023). In brief, five retinal organoids per genotype were pooled, flash-frozen, and stored at −80°C until the nuclei were extracted using chilled lysis buffer (10 mM Tris-HCl, 10 mM NaCl, 3 mM MgCl_2_, 0.01% Tween-20, 0.01% Nonidet P-40, 0.001% digitonin, 1 mM DTT, 1% BSA). The homogenate was incubated for 10 min on ice, washed twice with a resuspension buffer (10 mM Tris-HCl, 10 mM NaCl, 3 mM MgCl_2_, 0.1% Tween-20, 1 mM DTT, 1% BSA), and filtered through a series of 70 and 40 µm Flowmi filters. The nuclei were resuspended in an appropriate volume of diluted nucleus buffer (10x Genomics) to achieve a concentration of ∼2000–5000 nuclei/µL. Nucleus concentration was determined using trypan blue and DAPI staining.

### Single-nucleus multiomics library construction and sequencing

Single-nucleus RNA sequencing and ATAC-seq were performed on dissociated retinal nuclei using the Chromium single-cell multiome ATAC and gene expression reagent kits (10x Genomics). Briefly, nuclei (∼16,000 nuclei per sample) were processed for tagmentation and loaded onto the 10x Chromium controller, and the downstream RNA or ATAC libraries were generated and indexed according to the manufacturer's instructions. Libraries were pooled and sequenced on Illumina NovaSeq 6000 targeting 50,000 reads per cell for each RNA and ATAC library.

### Single-cell mutiomics analysis

The sequencing data from the single-cell RNA and ATAC libraries were demultiplexed and processed using Cell Ranger ARC v2.0.2 (10x Genomics). Reads were mapped to the GRCh38 reference genome, and count matrices were generated. Filtered feature–barcode matrices were loaded for analysis using the Seurat and Signac packages in R (Hao et al. 2021; Stuart et al. 2021). Initial quality control was performed on the RNA assay to filter low-quality cells, and doublets were identified using the scDblFinder R package (Germain et al. 2021). Nuclei with >3% of reads mapped to mitochondrial genes, <900 unique molecular identifiers (UMIs), or >50,000 UMIs were excluded. Data sets were merged and underwent normalization, variable feature selection, and scaling using Seurat's NormalizeData, FindVariableFeatures, and ScaleData functions. Principal components were then calculated and batch-corrected using the Harmony R package (Korsunsky et al. 2019). Uniform manifold approximation and projection (UMAP) dimension reduction was performed on the corrected principal components, and clusters were computed using Seurat's FindNeighbors and FindClusters functions. Cell types were then identified using a list of known marker genes that were used previously (Liu et al. 2023). ATAC peaks were called using MACS2 to generate peak sets for each cell type and then merged to create a consensus peak set, which was quantified for each cell (Zhang et al. 2008). Low-quality peaks and regions overlapping blacklisted genomic regions were filtered out. Further filtering of the data was performed by removing nuclei with <1000 or >50,000 ATAC fragments were removed. Additionally, nuclei were retained if they had a nucleosome signal of <2 and a TSS enrichment score >1. Latent semantic indexing (LSI) was applied to reduce the dimensionality of the peak matrix, followed by batch correction using Harmony. Gene activity scores and peak-to-gene links were calculated using Signac's GeneActivity and LinkPeaks functions. Motif analysis using the JASPAR2020 core vertebrates collection was performed, and ChromVAR was used to calculate motif accessibility deviations per cell (Schep et al. 2017; Fornes et al. 2020). Cell trajectories were calculated using the Slingshot R package, and the pseudotime estimates were used to visualize the progression of cells along inferred lineages (Street et al. 2018). The UCell R package was used to calculate enrichment scores for S cones, L/M cones, and rods using a curated set of genes published previously (Andreatta and Carmona 2021; Hussey et al. 2024).

### Random forest classification of hybrid photoreceptors

A random forest classifier was trained to assess cell identity probabilities of hybrid photoreceptors using the randomForest R package (Liaw and Wiener 2002). The training set consisted of control rod, S-cone, and L/M-cone photoreceptors, and the top 500 genes enriched in each cell type were used for feature selection. The model was then applied to the *DIO3*^Δ*33*^ hybrid cells to predict class probabilities for each photoreceptor type.

### Computational modeling

We simulated our mathematical model of photoreceptor specification using a hybrid deterministic–stochastic approach implemented in MATLAB. The concentration of T3—due to large numbers of individual molecules even at picomolar concentrations—was simulated deterministically via numerical integration. All other species in the model were simulated stochastically using the Gillespie algorithm. All model equations, stochastic events, rate functions, and parameters are detailed in the Supplemental Material. All curves in Figure 6B are single representative simulations of organoids seeded with 10^4^ RPCs. Simulations were run until each cell in the system adopted a terminal fate. The histograms in Figure 6C compile the results of 5000 replicate simulations, each of which was seeded with a randomly determined number of initial RPCs. Initial RPC numbers were drawn from a normal distribution with a mean of 10^4^ and a standard deviation of 2000. To ensure that the signaling and intrinsic model implementations were comparable, we adjusted the maximal rates of *S* and *L/M* photoreceptor specification in the intrinsic model (λ_*S*_ and λ_*L*_) such that the final average number of each photoreceptor matched the signaling model. In Figure 6D, each point summarizes the output of 5000 replicate simulations, each of which was initiated with a random number of RPCs, as in Figure 6C. Each set of simulations was run with a randomly seeded parameter set. Parameters were logarithmically sampled over a 100-fold range.

### Data and code availability

All sequencing data generated in this study can be accessed at GEO under accession number GSE292057 (wild-type vs. *DIO3*^Δ*33*^ mutant organoid single-nucleus multiomics). Code used to analyze the single-cell data sets in this study are available at https://github.com/csanti88/dio3_christina. Wild-type versus wild-type +T3-treated organoid single-cell RNA sequencing data were generated by Hussey et al. (2024).

### Ethics statement

Fetal retinas with no identifiers were obtained from donors to the Birth Defects Research Laboratory at the University of Washington. All procedures involving human tissue followed the Declaration of Helsinki and were done under an approved protocol (UW5R24HD000836) from the University of Washington. Age of the tissue was estimated using ultrasounds and some physical characteristics such as fetal foot length and crown–rump length.

## Supplemental Material

Supplement 1

Supplement 2

## References

[GAD352924MCNC1] Ahlgren SC, Wallace H, Bishop J, Neophytou C, Raff MC. 1997. Effects of thyroid hormone on embryonic oligodendrocyte precursor cell development in vivo and in vitro. Mol Cell Neurosci 9: 420–432. 10.1006/mcne.1997.0631 9361279

[GAD352924MCNC2] Alexiades MR, Cepko C. 1996. Quantitative analysis of proliferation and cell cycle length during development of the rat retina. Dev Dyn 205: 293–307. 10.1002/(SICI)1097-0177(199603)205:3<293::AID-AJA9>3.0.CO;2-D 8850565

[GAD352924MCNC3] Alexiades MR, Cepko CL. 1997. Subsets of retinal progenitors display temporally regulated and distinct biases in the fates of their progeny. Development 124: 1119–1131. 10.1242/dev.124.6.1119 9102299

[GAD352924MCNC4] Alisi A, Spagnuolo S, Napoletano S, Spaziani A, Leoni S. 2004. Thyroid hormones regulate DNA-synthesis and cell-cycle proteins by activation of PKCα and p42/44 MAPK in chick embryo hepatocytes. J Cell Physiol 201: 259–265. 10.1002/jcp.20060 15334660

[GAD352924MCNC5] Alkemade A, Vuijst CL, Unmehopa UA, Bakker O, Vennström B, Wiersinga WM, Swaab DF, Fliers E. 2005. Thyroid hormone receptor expression in the human hypothalamus and anterior pituitary. J Clin Endocrinol Metab 90: 904–912. 10.1210/jc.2004-0474 15562027

[GAD352924MCNC6] Andreatta M, Carmona SJ. 2021. UCell: robust and scalable single-cell gene signature scoring. Comput Struct Biotechnol J 19: 3796–3798. 10.1016/j.csbj.2021.06.043 34285779 PMC8271111

[GAD352924MCNC7] Andzelm MM, Cherry TJ, Harmin DA, Boeke AC, Lee C, Hemberg M, Pawlyk B, Malik AN, Flavell SW, Sandberg MA, 2015. MEF2D drives photoreceptor development through a genome-wide competition for tissue-specific enhancers. Neuron 86: 247–263. 10.1016/j.neuron.2015.02.038 25801704 PMC4393375

[GAD352924MCNC8] Aramaki M, Wu X, Liu H, Liu Y, Cho YW, Song M, Fu Y, Ng L, Forrest D. 2022. Transcriptional control of cone photoreceptor diversity by a thyroid hormone receptor. Proc Natl Acad Sci 119: e2209884119. 10.1073/pnas.2209884119 36454759 PMC9894165

[GAD352924MCNC9] Aranda A, Pascual A. 2001. Nuclear hormone receptors and gene expression. Physiol Rev 81: 1269–1304. 10.1152/physrev.2001.81.3.1269 11427696

[GAD352924MCNC10] Astapova I, Hollenberg AN. 2013. The in vivo role of nuclear receptor corepressors in thyroid hormone action. Biochim Biophys Acta 1830: 3876–3881. 10.1016/j.bbagen.2012.07.001 22801336 PMC3529203

[GAD352924MCNC11] Barres BA, Lazar MA, Raff MC. 1994. A novel role for thyroid hormone, glucocorticoids and retinoic acid in timing oligodendrocyte development. Development 120: 1097–1108. 10.1242/dev.120.5.1097 8026323

[GAD352924MCNC12] Belliveau MJ, Cepko CL. 1999. Extrinsic and intrinsic factors control the genesis of amacrine and cone cells in the rat retina. Development 126: 555–566. 10.1242/dev.126.3.555 9876184

[GAD352924MCNC13] Bianco AC, Salvatore D, Gereben B, Berry MJ, Larsen PR. 2002. Biochemistry, cellular and molecular biology, and physiological roles of the iodothyronine selenodeiodinases. Endocr Rev 23: 38–89. 10.1210/edrv.23.1.0455 11844744

[GAD352924MCNC14] Billon N, Tokumoto Y, Forrest D, Raff M. 2001. Role of thyroid hormone receptors in timing oligodendrocyte differentiation. Dev Biol 235: 110–120. 10.1006/dbio.2001.0293 11412031

[GAD352924MCNC15] Briscoe J, Ericson J. 1999. The specification of neuronal identity by graded Sonic Hedgehog signalling. Semin Cell Dev Biol 10: 353–362. 10.1006/scdb.1999.0295 10441550

[GAD352924MCNC16] Briscoe J, Pierani A, Jessell TM, Ericson J. 2000. A homeodomain protein code specifies progenitor cell identity and neuronal fate in the ventral neural tube. Cell 101: 435–445. 10.1016/S0092-8674(00)80853-3 10830170

[GAD352924MCNC17] Briscoe J, Chen Y, Jessell TM, Struhl G. 2001. A hedgehog-insensitive form of patched provides evidence for direct long-range morphogen activity of sonic hedgehog in the neural tube. Mol Cell 7: 1279–1291. 10.1016/S1097-2765(01)00271-4 11430830

[GAD352924MCNC18] Brzezinski JA, Reh TA. 2015. Photoreceptor cell fate specification in vertebrates. Development 142: 3263–3273. 10.1242/dev.127043 26443631 PMC4631758

[GAD352924MCNC19] Bumsted K, Hendrickson A. 1999. Distribution and development of short-wavelength cones differ between Macaca monkey and human fovea. J Comp Neurol 403: 502–516. 10.1002/(SICI)1096-9861(19990125)403:4<502::AID-CNE6>3.0.CO;2-N 9888315

[GAD352924MCNC20] Butler AJ, Parker MG. 1995. COUP-TF II homodimers are formed in preference to heterodimers with RXRα or TRβ in intact cells. Nucleic Acids Res 23: 4143–4150. 10.1093/nar/23.20.4143 7479078 PMC307356

[GAD352924MCNC21] Calza L, Fernandez M, Giuliani A, Aloe L, Giardino L. 2002. Thyroid hormone activates oligodendrocyte precursors and increases a myelin-forming protein and NGF content in the spinal cord during experimental allergic encephalomyelitis. Proc Natl Acad Sci 99: 3258–3263. 10.1073/pnas.052704499 11867745 PMC122506

[GAD352924MCNC22] Campos-Barros AA, Lori L, Faris JS, Shallam R, Kelley MW, Forrest D. 1999. Type 2 iodothyronine deiodinase expression in the cochlea before the onset of hearing. Proc Natl Acad Sci 97: 1287–1292. 10.1073/pnas.97.3.1287 PMC15599 10655523

[GAD352924MCNC23] Carre JL, Demerens C, Rodríguez-Peña A, Floch HH, Vincendon G, Sarliève LL. 1998. Thyroid hormone receptor isoforms are sequentially expressed in oligodendrocyte lineage cells during rat cerebral development. J Neurosci Res 54: 584–594. 10.1002/(SICI)1097-4547(19981201)54:5<584::AID-JNR3>3.0.CO;2-X 9843149

[GAD352924MCNC24] Cepko C. 2014. Intrinsically different retinal progenitor cells produce specific types of progeny. Nat Rev Neurosci 15: 615–627. 10.1038/nrn3767 25096185

[GAD352924MCNC25] Chen J, Rattner A, Nathans J. 2005. The rod photoreceptor-specific nuclear receptor Nr2e3 represses transcription of multiple cone-specific genes. J Neurosci 25: 118–129. 10.1523/JNEUROSCI.3571-04.2005 15634773 PMC6725199

[GAD352924MCNC26] Cho YW, Fu Y, Huang CJ, Wu X, Ng L, Kelley KA, Vella KR, Berg AH, Hollenberg AN, Liu H, 2023. Thyroid hormone-regulated chromatin landscape and transcriptional sensitivity of the pituitary gland. Commun Biol 6: 1253. 10.1038/s42003-023-05546-y 38081939 PMC10713718

[GAD352924MCNC27] Cornish EE, Hendrickson AE, Provis JM. 2004a. Distribution of short-wavelength-sensitive cones in human fetal and postnatal retina: early development of spatial order and density profiles. Vision Res 44: 2019–2026. 10.1016/j.visres.2004.03.030 15149835

[GAD352924MCNC28] Cornish EE, Xiao M, Yang Z, Provis JM, Hendrickson AE. 2004b. The role of opsin expression and apoptosis in determination of cone types in human retina. Exp Eye Res 78: 1143–1154. 10.1016/j.exer.2004.01.004 15109921

[GAD352924MCNC29] Courtin F, Zrouri H, Lamirand A, Li WW, Mercier G, Schumacher M, Goascogne CL, Pierre M. 2005. Thyroid hormone deiodinases in the central and peripheral nervous system. Thyroid 15: 931–942. 10.1089/thy.2005.15.931 16131335

[GAD352924MCNC30] Cowan CS, Renner M, De Gennaro M, Gross-Scherf B, Goldblum D, Hou Y, Munz M, Rodrigues TM, Krol J, Szikra T, 2020. Cell types of the human retina and its organoids at single-cell resolution. Cell 182: 1623–1640.e34. 10.1016/j.cell.2020.08.013 32946783 PMC7505495

[GAD352924MCNC31] Cuevas E, Holder DL, Alshehri AH, Tréguier J, Lakowski J, Sowden JC. 2021. *NRL*^−/−^ gene edited human embryonic stem cells generate rod-deficient retinal organoids enriched in S-cone-like photoreceptors. Stem Cells 39: 414–428. 10.1002/stem.3325 33400844 PMC8438615

[GAD352924MCNC32] Curcio CA, Sloan KR, Kalina RE, Hendrickson AE. 1990. Human photoreceptor topography. J Comp Neurol 292: 497–523. 10.1002/cne.902920402 2324310

[GAD352924MCNC33] Dessaud E, Ribes V, Balaskas N, Yang LL, Pierani A, Kicheva A, Novitch BG, Briscoe J, Sasai N. 2010. Dynamic assignment and maintenance of positional identity in the ventral neural tube by the morphogen sonic hedgehog. PLoS Biol 8: e1000382. 10.1371/journal.pbio.1000382 20532235 PMC2879390

[GAD352924MCNC34] Diaz-Araya C, Provis JM. 1992. Evidence of photoreceptor migration during early foveal development: a quantitative analysis of human fetal retinae. Vis Neurosci 8: 505–514. 10.1017/S0952523800005605 1586652

[GAD352924MCNC35] Dietrich JW, Landgrafe G, Fotiadou EH. 2012. TSH and thyrotropic agonists: key actors in thyroid homeostasis. J Thyroid Res 2012: 351864. 10.1155/2012/351864 23365787 PMC3544290

[GAD352924MCNC36] Douceau S, Deutsch Guerrero T, Ferent J. 2023. Establishing Hedgehog gradients during neural development. Cells 12: 225. 10.3390/cells12020225 36672161 PMC9856818

[GAD352924MCNC37] Driever W, Nüsslein-Volhard C. 1988. The bicoid protein determines position in the *Drosophila* embryo in a concentration-dependent manner. Cell 54: 95–104. 10.1016/0092-8674(88)90183-3 3383245

[GAD352924MCNC38] Durand B, Raff M. 2000. A cell-intrinsic timer that operates during oligodendrocyte development. Bioessays 22: 64–71. 10.1002/(SICI)1521-1878(200001)22:1<64::AID-BIES11>3.0.CO;2-Q 10649292

[GAD352924MCNC39] Eiraku M, Takata N, Ishibashi H, Kawada M, Sakakura E, Okuda S, Sekiguchi K, Adachi T, Sasai Y. 2011. Self-organizing optic-cup morphogenesis in three-dimensional culture. Nature 472: 51–56. 10.1038/nature09941 21475194

[GAD352924MCNC40] Eldred KC, Reh TA. 2021. Human retinal model systems: strengths, weaknesses, and future directions. Dev Biol 480: 114–122. 10.1016/j.ydbio.2021.09.001 34529997

[GAD352924MCNC41] Eldred KC, Hadyniak SE, Hussey KA, Brenerman B, Zhang PW, Chamling X, Sluch VM, Welsbie DS, Hattar S, Taylor J, 2018. Thyroid hormone signaling specifies cone subtypes in human retinal organoids. Science 362: eaau6348. 10.1126/science.aau6348 30309916 PMC6249681

[GAD352924MCNC42] Emerson MM, Surzenko N, Goetz JJ, Trimarchi J, Cepko CL. 2013. Otx2 and Onecut1 promote the fates of cone photoreceptors and horizontal cells and repress rod photoreceptors. Dev Cell 26: 59–72. 10.1016/j.devcel.2013.06.005 23867227 PMC3819454

[GAD352924MCNC43] Ericson J, Briscoe J, Rashbass P, van Heyningen V, Jessell TM. 1997a. Graded Sonic Hedgehog signaling and the specification of cell fate in the ventral neural tube. Cold Spring Harb Symp Quant Biol 62: 451–466. 10.1101/SQB.1997.062.01.053 9598380

[GAD352924MCNC44] Ericson J, Rashbass P, Schedl A, Brenner-Morton S, Kawakami A, van Heyningen V, Jessell TM, Briscoe J. 1997b. Pax6 controls progenitor cell identity and neuronal fate in response to graded Shh signaling. Cell 90: 169–180. 10.1016/S0092-8674(00)80323-2 9230312

[GAD352924MCNC45] Exelby K, Herrera-Delgado E, Perez LG, Perez-Carrasco R, Sagner A, Metzis V, Sollich P, Briscoe J. 2021. Precision of tissue patterning is controlled by dynamical properties of gene regulatory networks. Development 148: dev197566. 10.1242/dev.197566 33547135 PMC7929933

[GAD352924MCNC46] Fekete C, Lechan RM. 2014. Central regulation of hypothalamic–pituitary–thyroid axis under physiological and pathophysiological conditions. Endocr Rev 35: 159–194. 10.1210/er.2013-1087 24423980 PMC3963261

[GAD352924MCNC47] Feldt-Rasmussen U, Effraimidis G, Klose M. 2021. The hypothalamus–pituitary–thyroid (HPT)-axis and its role in physiology and pathophysiology of other hypothalamus–pituitary functions. Mol Cell Endocrinol 525: 111173. 10.1016/j.mce.2021.111173 33549603

[GAD352924MCNC48] Fischer AJ, Bongini R, Bastaki N, Sherwood P. 2011. The maturation of photoreceptors in the avian retina is stimulated by thyroid hormone. Neuroscience 178: 250–260. 10.1016/j.neuroscience.2011.01.022 21256198 PMC3048918

[GAD352924MCNC49] Fliers E, Unmehopa UA, Alkemade A. 2006. Functional neuroanatomy of thyroid hormone feedback in the human hypothalamus and pituitary gland. Mol Cell Endocrinol 251: 1–8. 10.1016/j.mce.2006.03.042 16707210

[GAD352924MCNC50] Fligor CM, Langer KB, Sridhar A, Ren Y, Shields PK, Edler MC, Ohlemacher SK, Sluch VM, Zack DJ, Zhang C, 2018. Three-dimensional retinal organoids facilitate the investigation of retinal ganglion cell development, organization and neurite outgrowth from human pluripotent stem cells. Sci Rep 8: 14520. 10.1038/s41598-018-32871-8 30266927 PMC6162218

[GAD352924MCNC51] Fornes O, Castro-Mondragon JA, Khan A, van der Lee R, Zhang X, Richmond PA, Modi BP, Correard S, Gheorghe M, Baranasic D, 2020. JASPAR 2020: update of the open-access database of transcription factor binding profiles. Nucleic Acids Res 48: D87–D92. 10.1093/nar/gkaa516 31701148 PMC7145627

[GAD352924MCNC52] Fuccillo M, Joyner AL, Fishell G. 2006. Morphogen to mitogen: the multiple roles of hedgehog signalling in vertebrate neural development. Nat Rev Neurosci 7: 772–783. 10.1038/nrn1990 16988653

[GAD352924MCNC53] Furukawa T, Morrow EM, Cepko CL. 1997. Crx, a novel otx-like homeobox gene, shows photoreceptor-specific expression and regulates photoreceptor differentiation. Cell 91: 531–541. 10.1016/S0092-8674(00)80439-0 9390562

[GAD352924MCNC54] Galton VA. 2017. The ups and downs of the thyroxine pro-hormone hypothesis. Mol Cell Endocrinol 458: 105–111. 10.1016/j.mce.2017.01.029 28130114

[GAD352924MCNC55] Gao FB, Apperly J, Raff M. 1998. Cell-intrinsic timers and thyroid hormone regulate the probability of cell-cycle withdrawal and differentiation of oligodendrocyte precursor cells. Dev Biol 197: 54–66. 10.1006/dbio.1998.8877 9578618

[GAD352924MCNC56] Gasparini SJ, Tessmer K, Reh M, Wieneke S, Carido M, Völkner M, Borsch O, Swiersy A, Zuzic M, Goureau O, 2022. Transplanted human cones incorporate into the retina and function in a murine cone degeneration model. J Clin Invest 132: e154619. 10.1172/JCI154619 35482419 PMC9197520

[GAD352924MCNC57] Gelfand RA, Hutchinson-Williams KA, Bonde AA, Castellino P, Sherwin RS. 1987. Catabolic effects of thyroid hormone excess: the contribution of adrenergic activity to hypermetabolism and protein breakdown. Metabolism 36: 562–569. 10.1016/0026-0495(87)90168-5 2884552

[GAD352924MCNC58] Gereben B, Zavacki AM, Ribich S, Kim BW, Huang SA, Simonides WS, Zeöld A, Bianco AC. 2008a. Cellular and molecular basis of deiodinase-regulated thyroid hormone signaling. Endocr Rev 29: 898–938. 10.1210/er.2008-0019 18815314 PMC2647704

[GAD352924MCNC59] Gereben B, Zeöld A, Dentice M, Salvatore D, Bianco AC. 2008b. Activation and inactivation of thyroid hormone by deiodinases: local action with general consequences. Cell Mol Life Sci 65: 570–590. 10.1007/s00018-007-7396-0 17989921 PMC11131710

[GAD352924MCNC60] Germain PL, Lun A, Garcia Meixide C, Macnair W, Robinson MD. 2021. Doublet identification in single-cell sequencing data using scDblFinder. F1000Res 10: 979. 10.12688/f1000research.73600.1 35814628 PMC9204188

[GAD352924MCNC61] Gomes FL, Zhang G, Carbonell F, Correa JA, Harris WA, Simons BD, Cayouette M. 2011. Reconstruction of rat retinal progenitor cell lineages in vitro reveals a surprising degree of stochasticity in cell fate decisions. Development 138: 227–235. 10.1242/dev.059683 21148186 PMC3005599

[GAD352924MCNC62] Guadaño-Ferraz A, Obregón MJ, St Germain DL, Bernal J. 1997. The type 2 iodothyronine deiodinase is expressed primarily in glial cells in the neonatal rat brain. Proc Natl Acad Sci 94: 10391–10396. 10.1073/pnas.94.19.10391 9294221 PMC23373

[GAD352924MCNC63] Guy B, Zhang JS, Duncan LH, Johnston RJ Jr. 2021. Human neural organoids: models for developmental neurobiology and disease. Dev Biol 478: 102–121. 10.1016/j.ydbio.2021.06.012 34181916 PMC8364509

[GAD352924MCNC64] Hadyniak SE, Hagen JFD, Eldred KC, Brenerman B, Hussey KA, McCoy RC, Sauria MEG, Kuchenbecker JA, Reh T, Glass I, 2024. Retinoic acid signaling regulates spatiotemporal specification of human green and red cones. PLoS Biol 22: e3002464. 10.1371/journal.pbio.3002464 38206904 PMC10783767

[GAD352924MCNC65] Hao Y, Hao S, Andersen-Nissen E, Mauck WM, Zheng S, Butler A, Lee MJ, Wilk AJ, Darby C, Zager M, 2021. Integrated analysis of multimodal single-cell data. Cell 184: 3573–3587.e29. 10.1016/j.cell.2021.04.048 34062119 PMC8238499

[GAD352924MCNC66] He J, Zhang G, Almeida AD, Cayouette M, Simons BD, Harris WA. 2012. How variable clones build an invariant retina. Neuron 75: 786–798. 10.1016/j.neuron.2012.06.033 22958820 PMC3485567

[GAD352924MCNC67] Hernandez A, Fiering S, Martinez E, Galton VA, St. Germain D. 2002. The gene *locus* encoding iodothyronine deiodinase type 3 (*Dio3*) is imprinted in the fetus and expresses antisense transcripts. Endocrinology 143: 4483–4486. 10.1210/en.2002-220800 12399446

[GAD352924MCNC68] Hernandez A, Martinez ME, Fiering S, Galton VA, St. Germain D. 2006. Type 3 deiodinase is critical for the maturation and function of the thyroid axis. J Clin Invest 116: 476–484. 10.1172/JCI26240 16410833 PMC1326144

[GAD352924MCNC69] Hoshino A, Ratnapriya R, Brooks MJ, Chaitankar V, Wilken MS, Zhang C, Starostik MR, Gieser L, La Torre A, Nishio M, 2017. Molecular anatomy of the developing human retina. Dev Cell 43: 763–779.e4. 10.1016/j.devcel.2017.10.029 29233477 PMC5776731

[GAD352924MCNC70] Hussey KA, Hadyniak SE, Johnston RJ Jr. 2022. Patterning and development of photoreceptors in the human retina. Front Cell Dev Biol 10: 878350. 10.3389/fcell.2022.878350 35493094 PMC9049932

[GAD352924MCNC71] Hussey KA, Eldred K, Guy B, Santiago C, Glass I, Reh TA, Blackshaw S, Goff LA, Johnston RJ. 2024. Cell fate specification and conversion generate foveolar cone subtype patterning in human retinal organoids. bioRxiv 10.1101/2023.01.28.526051 PMC12913016 41686445

[GAD352924MCNC72] Iwama Y, Sugase-Miyamoto Y, Onoue K, Uyama H, Matsuda K, Hayashi K, Akiba R, Masuda T, Yokota S, Yonemura S, 2024. Transplantation of human pluripotent stem cell-derived retinal sheet in a primate model of macular hole. Stem Cell Reports 19: 1524–1533. 10.1016/j.stemcr.2024.09.002 39366379 PMC11589285

[GAD352924MCNC73] Jessell TM. 2000. Neuronal specification in the spinal cord: inductive signals and transcriptional codes. Nat Rev Genet 1: 20–29. 10.1038/35049541 11262869

[GAD352924MCNC74] Kaewkhaw R, Kaya KD, Brooks M, Homma K, Zou J, Chaitankar V, Rao M, Swaroop A. 2015. Transcriptome dynamics of developing photoreceptors in three-dimensional retina cultures recapitulates temporal sequence of human cone and rod differentiation revealing cell surface markers and gene networks. Stem Cells 33: 3504–3518. 10.1002/stem.2122 26235913 PMC4713319

[GAD352924MCNC75] Kallman A, Capowski EE, Wang J, Kaushik AM, Jansen AD, Edwards KL, Chen L, Berlinicke CA, Joseph Phillips M, Pierce EA, 2020. Investigating cone photoreceptor development using patient-derived NRL null retinal organoids. Commun Biol 3: 82. 10.1038/s42003-020-0808-5 32081919 PMC7035245

[GAD352924MCNC76] Kandoi S, Martinez C, Chen KX, Mehine M, Reddy LVK, Mansfield BC, Duncan JL, Lamba DA. 2024. Disease modeling and pharmacological rescue of autosomal dominant retinitis pigmentosa associated with RHO copy number variation. eLife 12: RP90575. 10.7554/eLife.90575 38661530 PMC11045220

[GAD352924MCNC77] Kelley MW, Williams RC, Turner JK, Creech-Kraft JM, Reh TA. 1999. Retinoic acid promotes rod photoreceptor differentiation in rat retina in vivo. Neuroreport 10: 2389–2394. 10.1097/00001756-199908020-00031 10439469

[GAD352924MCNC78] Khanna H, Akimoto M, Siffroi-Fernandez S, Friedman JS, Hicks D, Swaroop A. 2006. Retinoic acid regulates the expression of photoreceptor transcription factor NRL. J Biol Chem 281: 27327–27334. 10.1074/jbc.M605500200 16854989 PMC1592579

[GAD352924MCNC79] Koenig RJ. 1998. Thyroid hormone receptor coactivators and corepressors. Thyroid 8: 703–713. 10.1089/thy.1998.8.703 9737367

[GAD352924MCNC80] Korsunsky I, Millard N, Fan J, Slowikowski K, Zhang F, Wei K, Baglaenko Y, Brenner M, Loh PR, Raychaudhuri S. 2019. Fast, sensitive and accurate integration of single-cell data with Harmony. Nat Methods 16: 1289–1296. 10.1038/s41592-019-0619-0 31740819 PMC6884693

[GAD352924MCNC81] Larsen PR. 1982. Thyroid–pituitary interaction: feedback regulation of thyrotropin secretion by thyroid hormones. N Engl J Med 306: 23–32. 10.1056/NEJM198201073060107 7031472

[GAD352924MCNC82] La Torre A, Georgi S, Reh TA. 2013. Conserved microRNA pathway regulates developmental timing of retinal neurogenesis. Proc Natl Acad Sci 110: E2362–E2370. 10.1073/pnas.1301837110 23754433 PMC3696811

[GAD352924MCNC83] Lee JY, Petratos S. 2016. Thyroid hormone signaling in oligodendrocytes: from extracellular transport to intracellular signal. Mol Neurobiol 53: 6568–6583. 10.1007/s12035-016-0013-1 27427390

[GAD352924MCNC84] Li X, Zhang L, Tang F, Wei X. 2021. Retinal organoids: cultivation, differentiation, and transplantation. Front Cell Neurosci 15: 638439. 10.3389/fncel.2021.638439 34276307 PMC8282056

[GAD352924MCNC85] Liaw A, Wiener M. 2002. Classification and regression by randomForest. R News 2: 18–22.

[GAD352924MCNC86] Liu YV, Santiago CP, Sogunro A, Konar GJ, Hu MW, McNally MM, Lu YC, Flores-Bellver M, Aparicio-Domingo S, Li KV, 2023. Single-cell transcriptome analysis of xenotransplanted human retinal organoids defines two migratory cell populations of nonretinal origin. Stem Cell Reports 18: 1138–1154. 10.1016/j.stemcr.2023.04.004 37163980 PMC10202694

[GAD352924MCNC87] Lord ND, Norman TM, Yuan R, Bakshi S, Losick R, Paulsson J. 2019. Stochastic antagonism between two proteins governs a bacterial cell fate switch. Science 366: 116–120. 10.1126/science.aaw4506 31604312 PMC7526939

[GAD352924MCNC88] Lu A, Ng L, Ma M, Kefas B, Davies TF, Hernandez A, Chan CC, Forrest D. 2009. Retarded developmental expression and patterning of retinal cone opsins in hypothyroid mice. Endocrinology 150: 1536–1544. 10.1210/en.2008-1092 18974269 PMC2654753

[GAD352924MCNC89] Lu Y, Shiau F, Yi W, Lu S, Wu Q, Pearson JD, Kallman A, Zhong S, Hoang T, Zuo Z, 2020. Single-cell analysis of human retina identifies evolutionarily conserved and species-specific mechanisms controlling development. Dev Cell 53: 473–491.e9. 10.1016/j.devcel.2020.04.009 32386599 PMC8015270

[GAD352924MCNC90] Lyu P, Hoang T, Santiago CP, Thomas ED, Timms AE, Appel H, Gimmen M, Le N, Jiang L, Kim DW, 2021. Gene regulatory networks controlling temporal patterning, neurogenesis, and cell-fate specification in mammalian retina. Cell Rep 37: 109994. 10.1016/j.celrep.2021.109994 34788628 PMC8642835

[GAD352924MCNC91] Ma H, Thapa A, Morris L, Redmond TM, Baehr W, Ding XQ. 2014. Suppressing thyroid hormone signaling preserves cone photoreceptors in mouse models of retinal degeneration. Proc Natl Acad Sci 111: 3602–3607. 10.1073/pnas.1317041111 24550448 PMC3948228

[GAD352924MCNC92] Mackin RD, Frey RA, Gutierrez C, Farre AA, Kawamura S, Mitchell DM, Stenkamp DL. 2019. Endocrine regulation of multichromatic color vision. Proc Natl Acad Sci 116: 16882–16891. 10.1073/pnas.1904783116 31383755 PMC6708328

[GAD352924MCNC93] McNerney C, Johnston RJ Jr. 2021. Thyroid hormone signaling specifies cone photoreceptor subtypes during eye development: insights from model organisms and human stem cell-derived retinal organoids. Vitam Horm 116: 51–90. 10.1016/bs.vh.2021.03.001 33752828 PMC8376222

[GAD352924MCNC94] Mears AJ, Kondo M, Swain PK, Takada Y, Bush RA, Saunders TL, Sieving PA, Swaroop A. 2001. Nrl is required for rod photoreceptor development. Nat Genet 29: 447–452. 10.1038/ng774 11694879

[GAD352924MCNC95] Müller J, Mayerl S, Visser TJ, Darras VM, Boelen A, Frappart L, Mariotta L, Verrey F, Heuer H. 2014. Tissue-specific alterations in thyroid hormone homeostasis in combined Mct10 and Mct8 deficiency. Endocrinology 155: 315–325. 10.1210/en.2013-1800 24248460

[GAD352924MCNC96] Nakano T, Ando S, Takata N, Kawada M, Muguruma K, Sekiguchi K, Saito K, Yonemura S, Eiraku M, Sasai Y. 2012. Self-formation of optic cups and storable stratified neural retina from human ESCs. Cell Stem Cell 10: 771–785. 10.1016/j.stem.2012.05.009 22704518

[GAD352924MCNC97] Nathans J, Thomas D, Hogness DS. 1986. Molecular genetics of human color vision: the genes encoding blue, green, and red pigments. Science 232: 193–202. 10.1126/science.2937147 2937147

[GAD352924MCNC98] Ng L, Hurley JB, Dierks B, Srinivas M, Saltó C, Vennström B, Reh TA, Forrest D. 2001. A thyroid hormone receptor that is required for the development of green cone photoreceptors. Nat Genet 27: 94–98. 10.1038/83829 11138006

[GAD352924MCNC99] Ng L, Goodyear RJ, Woods CA, Schneider MJ, Diamond E, Richardson GP, Kelley MW, Germain DL, Galton VA, Forrest D. 2004. Hearing loss and retarded cochlear development in mice lacking type 2 iodothyronine deiodinase. Proc Natl Acad Sci 101: 3474–3479. 10.1073/pnas.0307402101 14993610 PMC373486

[GAD352924MCNC100] Ng L, Hernandez A, He W, Ren T, Srinivas M, Ma M, Galton VA, St Germain DL, Forrest D. 2009. A protective role for type 3 deiodinase, a thyroid hormone-inactivating enzyme, in cochlear development and auditory function. Endocrinology 150: 1952–1960. 10.1210/en.2008-1419 19095741 PMC2659284

[GAD352924MCNC101] Ng L, Lyubarsky A, Nikonov SS, Ma M, Srinivas M, Kefas B, St. Germain DL, Hernandez A, Pugh EN Jr., Forrest D. 2010. Type 3 deiodinase, a thyroid-hormone-inactivating enzyme, controls survival and maturation of cone photoreceptors. J Neurosci 30: 3347–3357. 10.1523/JNEUROSCI.5267-09.2010 20203194 PMC2843520

[GAD352924MCNC102] Ng L, Lu A, Swaroop A, Sharlin DS, Swaroop A, Forrest D. 2011. Two transcription factors can direct three photoreceptor outcomes from rod precursor cells in mouse retinal development. J Neurosci 31: 11118–11125. 10.1523/JNEUROSCI.1709-11.2011 21813673 PMC3158567

[GAD352924MCNC103] Ng L, Liu H, St. Germain DL, Hernandez A, Forrest D. 2017. Deletion of the thyroid hormone-activating type 2 deiodinase rescues cone photoreceptor degeneration but not deafness in mice lacking type 3 deiodinase. Endocrinology 158: 1999–2010. 10.1210/en.2017-00055 28324012 PMC5460942

[GAD352924MCNC104] Nicolini G, Casini G, Posarelli C, Amato R, Lulli M, Balzan S, Forini F. 2024. Thyroid hormone signaling in retinal development and function: implications for diabetic retinopathy and age-related macular degeneration. Int J Mol Sci 25: 7364. 10.3390/ijms25137364 39000471 PMC11242054

[GAD352924MCNC105] Norman TM, Lord ND, Paulsson J, Losick R. 2013. Memory and modularity in cell-fate decision making. Nature 503: 481–486. 10.1038/nature12804 24256735 PMC4019345

[GAD352924MCNC106] Oh EC, Cheng H, Hao H, Jia L, Khan NW, Swaroop A. 2008. Rod differentiation factor NRL activates the expression of nuclear receptor NR2E3 to suppress the development of cone photoreceptors. Brain Res 1236: 16–29. 10.1016/j.brainres.2008.01.028 18294621 PMC2660138

[GAD352924MCNC107] O'Hara-Wright M, Gonzalez-Cordero A. 2020. Retinal organoids: a window into human retinal development. Development 147: dev189746. 10.1242/dev.189746 33361444 PMC7774906

[GAD352924MCNC108] Patten I, Placzek M. 2002. Opponent activities of Shh and BMP signaling during floor plate induction in vivo. Curr Biol 12: 47–52. 10.1016/S0960-9822(01)00631-5 11790302

[GAD352924MCNC109] Peng YR, Shekhar K, Yan W, Herrmann D, Sappington A, Bryman GS, van Zyl T, Do MTH, Regev A, Sanes JR. 2019. Molecular classification and comparative taxonomics of foveal and peripheral cells in primate retina. Cell 176: 1222–1237.e22. 10.1016/j.cell.2019.01.004 30712875 PMC6424338

[GAD352924MCNC110] Phillips MJ, Perez ET, Martin JM, Reshel ST, Wallace KA, Capowski EE, Singh R, Wright LS, Clark EM, Barney PM, 2014. Modeling human retinal development with patient-specific induced pluripotent stem cells reveals multiple roles for visual system homeobox 2. Stem Cells 32: 1480–1492. 10.1002/stem.1667 24532057 PMC4037340

[GAD352924MCNC111] Phillips MJ, Jiang P, Howden S, Barney P, Min J, York NW, Chu LF, Capowski EE, Cash A, Jain S, 2018. A novel approach to single cell RNA-sequence analysis facilitates in silico gene reporting of human pluripotent stem cell-derived retinal cell types. Stem Cells 36: 313–324. 10.1002/stem.2755 29230913 PMC5823737

[GAD352924MCNC112] Porlan E, Vega S, Iglesias T, Rodríguez-Peña A. 2004. Unliganded thyroid hormone receptor β1 inhibits proliferation of murine fibroblasts by delaying the onset of the G1 cell-cycle signals. Oncogene 23: 8756–8765. 10.1038/sj.onc.1208126 15467737

[GAD352924MCNC113] Potvin-Trottier L, Lord ND, Vinnicombe G, Paulsson J. 2016. Synchronous long-term oscillations in a synthetic gene circuit. Nature 538: 514–517. 10.1038/nature19841 27732583 PMC5637407

[GAD352924MCNC114] Prabhudesai SN, Cameron DA, Stenkamp DL. 2005. Targeted effects of retinoic acid signaling upon photoreceptor development in zebrafish. Dev Biol 287: 157–167. 10.1016/j.ydbio.2005.08.045 16197938 PMC2804901

[GAD352924MCNC115] Roberts MR, Srinivas M, Forrest D, Morreale de Escobar G, Reh TA. 2006. Making the gradient: thyroid hormone regulates cone opsin expression in the developing mouse retina. Proc Natl Acad Sci 103: 6218–6223. 10.1073/pnas.0509981103 16606843 PMC1458858

[GAD352924MCNC116] Rodríguez-Peña A. 1999. Oligodendrocyte development and thyroid hormone. J Neurobiol 40: 497–512. 10.1002/(sici)1097-4695(19990915)40:4<497::AID-NEU7>3.0.CO;2-# 10453052

[GAD352924MCNC117] Sakagami K, Gan L, Yang XJ. 2009. Distinct effects of Hedgehog signaling on neuronal fate specification and cell cycle progression in the embryonic mouse retina. J Neurosci 29: 6932–6944. 10.1523/JNEUROSCI.0289-09.2009 19474320 PMC2715855

[GAD352924MCNC118] Santiago CP, Gimmen MY, Lu Y, McNally MM, Duncan LH, Creamer TJ, Orzolek LD, Blackshaw S, Singh MS. 2023. Comparative analysis of single-cell and single-nucleus RNA-sequencing in a rabbit model of retinal detachment-related proliferative vitreoretinopathy. Ophthalmol Sci 3: 100335. 10.1016/j.xops.2023.100335 37496518 PMC10365955

[GAD352924MCNC119] Schep AN, Wu B, Buenrostro JD, Greenleaf WJ. 2017. chromVAR: inferring transcription-factor-associated accessibility from single-cell epigenomic data. Nat Methods 14: 975–978. 10.1038/nmeth.4401 28825706 PMC5623146

[GAD352924MCNC120] Schick E, McCaffery SD, Keblish EE, Thakurdin C, Emerson MM. 2019. Lineage tracing analysis of cone photoreceptor associated *cis*-regulatory elements in the developing chicken retina. Sci Rep 9: 9358. 10.1038/s41598-019-45750-7 31249345 PMC6597718

[GAD352924MCNC121] Shirai H, Mandai M, Matsushita K, Kuwahara A, Yonemura S, Nakano T, Assawachananont J, Kimura T, Saito K, Terasaki H, 2016. Transplantation of human embryonic stem cell-derived retinal tissue in two primate models of retinal degeneration. Proc Natl Acad Sci 113: E81–E90. 10.1073/pnas.1512590113 26699487 PMC4711854

[GAD352924MCNC122] Singh RK, Occelli LM, Binette F, Petersen-Jones SM, Nasonkin IO. 2019. Transplantation of human embryonic stem cell-derived retinal tissue in the subretinal space of the cat eye. Stem Cells Dev 28: 1151–1166. 10.1089/scd.2019.0090 31210100 PMC6708274

[GAD352924MCNC123] Sockanathan S, Jessell TM. 1998. Motor neuron-derived retinoid signaling specifies the subtype identity of spinal motor neurons. Cell 94: 503–514. 10.1016/S0092-8674(00)81591-3 9727493

[GAD352924MCNC124] Sridhar A, Hoshino A, Finkbeiner CR, Chitsazan A, Dai L, Haugan AK, Eschenbacher KM, Jackson DL, Trapnell C, Bermingham-McDonogh O, 2020. Single-cell transcriptomic comparison of human fetal retina, hPSC-derived retinal organoids, and long-term retinal cultures. Cell Rep 30: 1644–1659.e4. 10.1016/j.celrep.2020.01.007 32023475 PMC7901645

[GAD352924MCNC125] Stamataki D, Ulloa F, Tsoni SV, Mynett A, Briscoe J. 2005. A gradient of Gli activity mediates graded Sonic Hedgehog signaling in the neural tube. Genes Dev 19: 626–641. 10.1101/gad.325905 15741323 PMC551582

[GAD352924MCNC126] Stenkamp DL, Frey RA. 2003. Extraretinal and retinal hedgehog signaling sequentially regulate retinal differentiation in zebrafish. Dev Biol 258: 349–363. 10.1016/S0012-1606(03)00121-0 12798293

[GAD352924MCNC127] Street K, Risso D, Fletcher RB, Das D, Ngai J, Yosef N, Purdom E, Dudoit S. 2018. Slingshot: cell lineage and pseudotime inference for single-cell transcriptomics. BMC Genomics 19: 477. 10.1186/s12864-018-4772-0 29914354 PMC6007078

[GAD352924MCNC128] Stuart T, Srivastava A, Madad S, Lareau CA, Satija R. 2021. Single-cell chromatin state analysis with Signac. Nat Methods 18: 1333–1341. 10.1038/s41592-021-01282-5 34725479 PMC9255697

[GAD352924MCNC129] Trimarchi JM, Harpavat S, Billings NA, Cepko CL. 2008. Thyroid hormone components are expressed in three sequential waves during development of the chick retina. BMC Dev Biol 8: 101. 10.1186/1471-213X-8-101 18854032 PMC2579430

[GAD352924MCNC130] Tu HM, Kim SW, Salvatore D, Bartha T, Legradi G, Larsen PR, Lechan RM. 1997. Regional distribution of type 2 thyroxine deiodinase messenger ribonucleic acid in rat hypothalamus and pituitary and its regulation by thyroid hormone. Endocrinology 138: 3359–3368. 10.1210/endo.138.8.5318 9231788

[GAD352924MCNC131] Tu HM, Legradi G, Bartha T, Salvatore D, Lechan RM, Larsen PR. 1999. Regional expression of the type 3 iodothyronine deiodinase messenger ribonucleic acid in the rat central nervous system and its regulation by thyroid hormone. Endocrinology 140: 784–790. 10.1210/endo.140.2.6486 9927306

[GAD352924MCNC132] Viets K, Eldred KC, Johnston RJ Jr. 2016. Mechanisms of photoreceptor patterning in vertebrates and invertebrates. Trends Genet 32: 638–659. 10.1016/j.tig.2016.07.004 27615122 PMC5035628

[GAD352924MCNC133] Wahlin KJ, Maruotti JA, Sripathi SR, Ball J, Angueyra JM, Kim C, Grebe R, Li W, Jones BW, Zack DJ. 2017. Photoreceptor outer segment-like structures in long-term 3D retinas from human pluripotent stem cells. Sci Rep 7: 766. 10.1038/s41598-017-00774-9 28396597 PMC5429674

[GAD352924MCNC134] Wahlin KJ, Cheng J, Jurlina SL, Jones MK, Dash NR, Ogata A, Kibria N, Ray S, Eldred KC, Kim C, 2021. CRISPR generated SIX6 and POU4F2 reporters allow identification of brain and optic transcriptional differences in human PSC-derived organoids. Front Cell Dev Biol 9: 764725. 10.3389/fcell.2021.764725 34869356 PMC8635054

[GAD352924MCNC135] Wang Y, Dakubo GD, Thurig S, Mazerolle CJ, Wallace VA. 2005. Retinal ganglion cell-derived sonic hedgehog locally controls proliferation and the timing of RGC development in the embryonic mouse retina. Development 132: 5103–5113. 10.1242/dev.02096 16236765

[GAD352924MCNC136] Wang S, Sengel C, Emerson MM, Cepko CL. 2014. A gene regulatory network controls the binary fate decision of rod and bipolar cells in the vertebrate retina. Dev Cell 30: 513–527. 10.1016/j.devcel.2014.07.018 25155555 PMC4304698

[GAD352924MCNC137] Watari K, Yamasaki S, Tu HY, Shikamura M, Kamei T, Adachi H, Tochitani T, Kita Y, Nakamura A, Ueyama K, 2023. Self-organization, quality control, and preclinical studies of human iPSC-derived retinal sheets for tissue-transplantation therapy. Commun Biol 6: 164. 10.1038/s42003-023-04543-5 36765170 PMC9918541

[GAD352924MCNC138] Wolpert L. 1969. Positional information and the spatial pattern of cellular differentiation. J Theor Biol 25: 1–47. 10.1016/S0022-5193(69)80016-0 4390734

[GAD352924MCNC139] Xiao M, Hendrickson A. 2000. Spatial and temporal expression of short, long/medium, or both opsins in human fetal cones. J Comp Neurol 425: 545–559. 10.1002/1096-9861(20001002)425:4<545::AID-CNE6>3.0.CO;2-3 10975879

[GAD352924MCNC140] Zhang XM, Yang XJ. 2001. Regulation of retinal ganglion cell production by Sonic hedgehog. Development 128: 943–957. 10.1242/dev.128.6.943 11222148 PMC7048390

[GAD352924MCNC141] Zhang XK, Hoffmann B, Tran PB, Graupner G, Pfahl M. 1992. Retinoid X receptor is an auxiliary protein for thyroid hormone and retinoic acid receptors. Nature 355: 441–446. 10.1038/355441a0 1310350

[GAD352924MCNC142] Zhang Y, Liu T, Meyer CA, Eeckhoute J, Johnson DS, Bernstein BE, Nusbaum C, Myers RM, Brown M, Li W, 2008. Model-based analysis of ChIP-seq (MACS). Genome Biol 9: R137. 10.1186/gb-2008-9-9-r137 18798982 PMC2592715

[GAD352924MCNC143] Zhang J, Choi EH, Tworak A, Salom D, Leinonen H, Sander CL, Hoang TV, Handa JT, Blackshaw S, Palczewska G, 2019. Photic generation of 11-*cis*-retinal in bovine retinal pigment epithelium. J Biol Chem 294: 19137–19154. 10.1074/jbc.RA119.011169 31694912 PMC6916499

[GAD352924MCNC144] Zhang X, Leavey P, Appel H, Makrides N, Blackshaw S. 2023a. Molecular mechanisms controlling vertebrate retinal patterning, neurogenesis, and cell fate specification. Trends Genet 39: 736–757. 10.1016/j.tig.2023.06.002 37423870 PMC10529299

[GAD352924MCNC145] Zhang X, Wang X, Hu H, Qu H, Xu Y, Li Q. 2023b. Prevalence and trends of thyroid disease among adults, 1999–2018. Endocr Pract 29: 875–880. 10.1016/j.eprac.2023.08.006 37619827

[GAD352924MCNC146] Zou T, Gao L, Zeng Y, Li Q, Li Y, Chen S, Hu X, Chen X, Fu C, Xu H, 2019. Organoid-derived C-Kit^+^/SSEA4^+^ human retinal progenitor cells promote a protective retinal microenvironment during transplantation in rodents. Nat Commun 10: 1205. 10.1038/s41467-019-08961-0 30872578 PMC6418223

[GAD352924MCNC147] Zuo Z, Cheng X, Ferdous S, Shao J, Li J, Bao Y, Li J, Lu J, Jacobo Lopez A, Wohlschlegel J, 2024. Single cell dual-omic atlas of the human developing retina. Nat Commun 15: 6792. 10.1038/s41467-024-50853-5 39117640 PMC11310509

