# Supplementary material for: DIO3 coordinates photoreceptor development timing and fate stability in human retinal organoids
Source: Genes Dev. 2026 Jan 1;40(1-2):70–93. doi: 10.1101/gad.352924.125 (PMC12758142; doi:10.1101/gad.352924.125)
Supplement: Supplement 1 [file Supplemental_Figs.pdf]

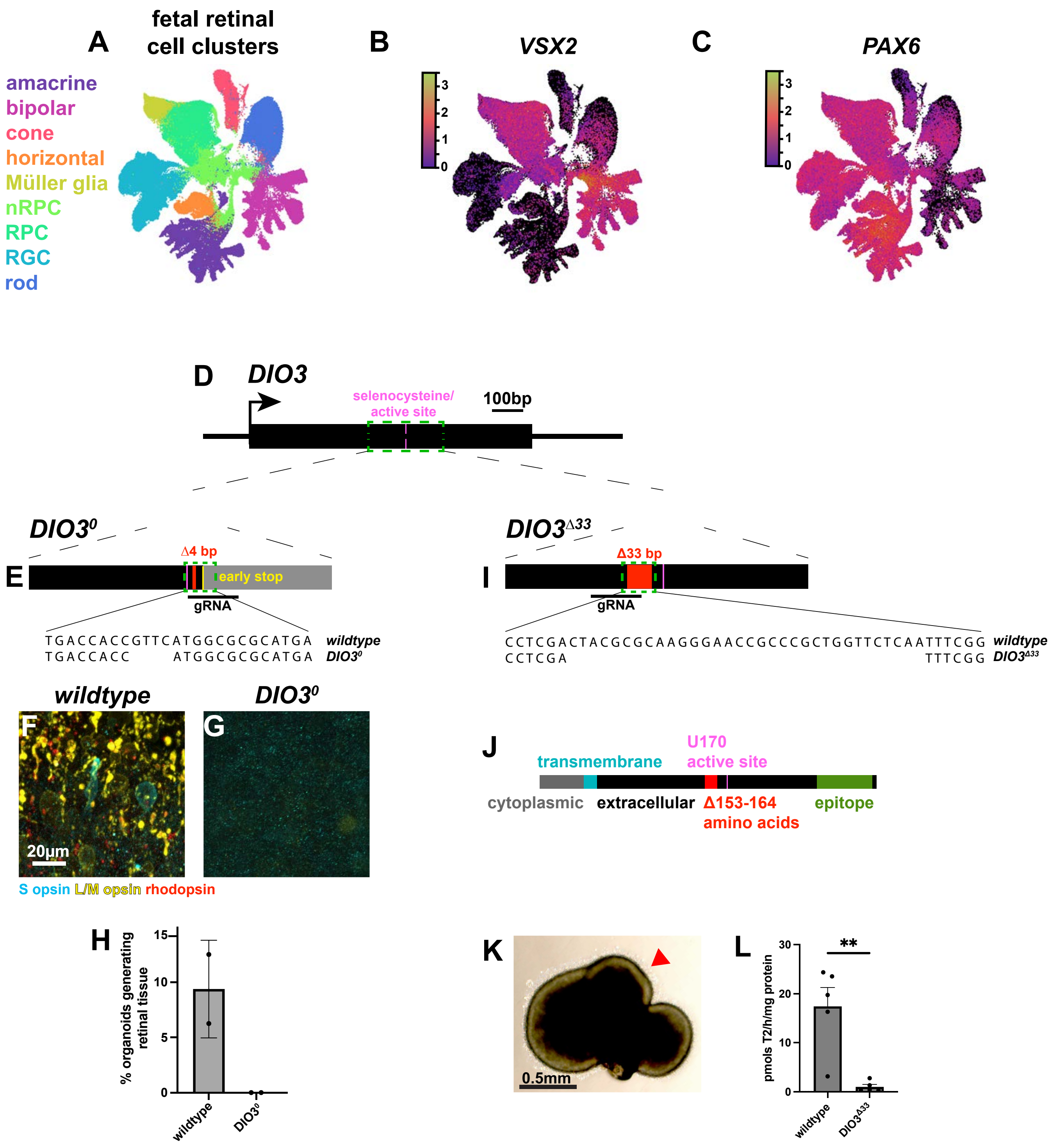

Supplemental Figure 1

Supplemental Fig. 1. Identification of fetal RPC clusters, generation and characterization of DIO3 mutants.

(A) UMAP from CZ CELLxGENE Discover (Zuo et al., 2024a) of human fetal scRNA-seq with major cell types labeled, as in Fig. 1A.

(B) *VSX2* expression in fetal human retina limited to *VSX2*+ cells, overlaid on the unannotated UMAP.

(C) *PAX6* expression in fetal human retina limited to *PAX6*+ cells, overlaid on the unannotated UMAP.

(D) *DIO3* locus. Selenocysteine active site marked in pink. Zoom in on green dashed region in **Fig. S1E** and **Fig. S1I**.

(E) *DIO3*^{0} mutation. The 4 base pair deletion (red) induced an early stop (yellow). Below, the *wildtype* and *DIO3*^{0} sequences of this region.

(F-G) Day 200 *wildtype* (F) and *DIO3*^{0} (G) stained for S-opsin, L/M-opsin, rhodopsin.

(H) Percent of *wildtype* and *DIO3*^{0} organoids generating retinal tissue. N=384 organoids, 2 independent differentiations, each with *wildtype* and *DIO3*^{0} grown in parallel.

(I) *DIO3*$^{\Delta 33}$ with a 33 base pair in-frame deletion (red). Below, the wildtype and *DIO3*$^{\Delta 33}$ allele sequences.

(J) Protein sequence of wildtype DIO3. Teal = transmembrane region. Magenta = selenocysteine active site. Grey = cytoplasmic domain. Black = extracellular domain. Red = ∆33bp/11amino acid region deleted in *DIO3*$^{\Delta 33}$ mutation.

(K) *DIO3*$^{\Delta 33}$ organoid at day 200 at 4x magnification. Red arrowhead indicates photoreceptor outer segments.

(L) Deiodination activities in *wildtype* and *DIO3*$^{\Delta 33}$ organoids at day 76. Error bars represent SEM. p value= 0.0029 by unpaired t-test.

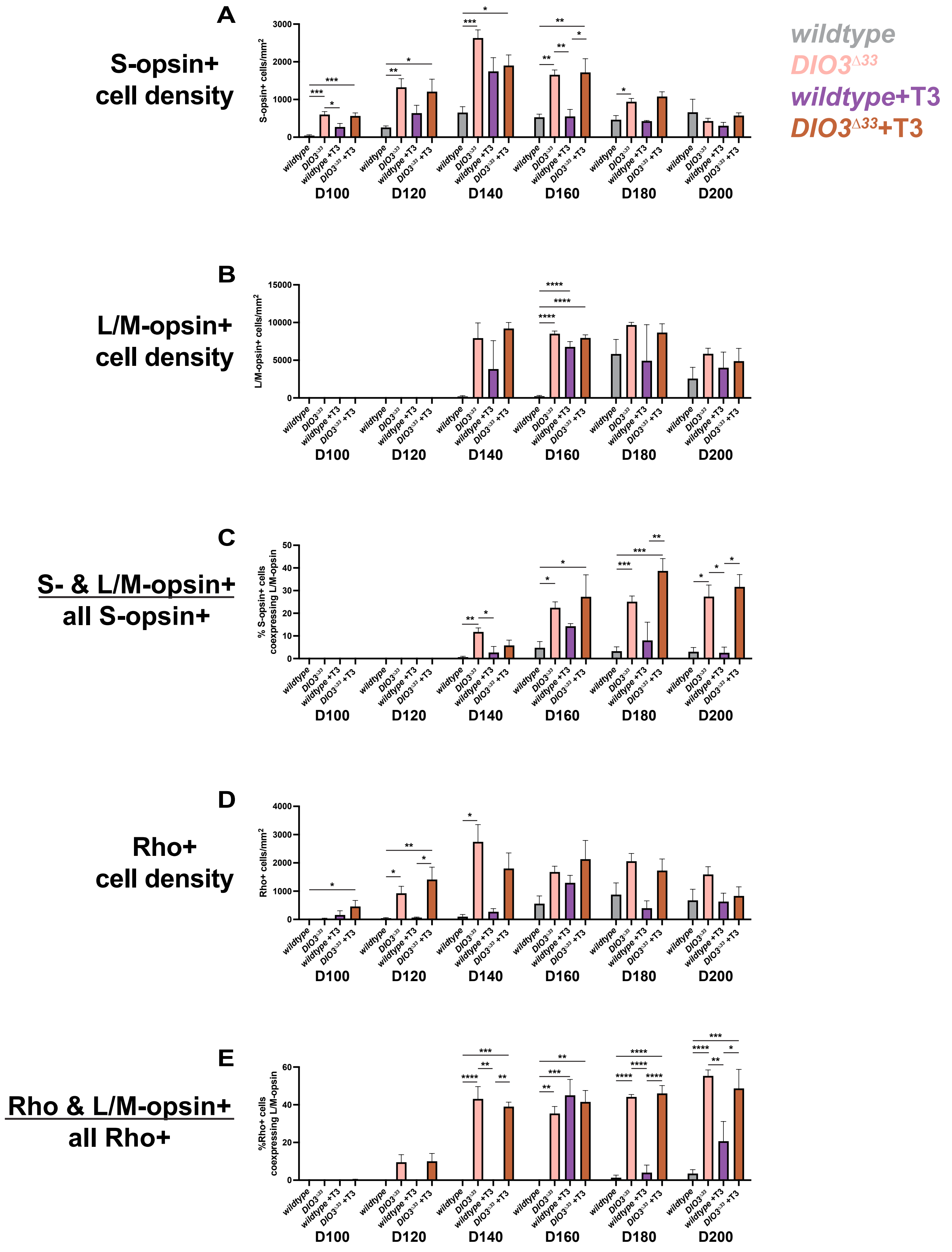

Supplemental Figure 2

Supplemental Fig. 2. Comparison of photoreceptor development over time.

Data as in **Fig. 2G, I, K, M**, **S3Q-T,** re-plotted to compare per timepoint across conditions. Graph colors consistent with **Fig. 2E** per condition. Error bars represent SEM, statistics determined via one-way ANOVA with Tukey's multiple comparisons test unless otherwise noted. Unlabeled comparisons are not significant (p>0.05).

(A) S-opsin+ cell density.

D100 ANOVA p=0.0002

wildtype vs *DIO3*$^{\Delta 33}$ p= 0.0002

wildtype vs *DIO3*$^{\Delta 33}$+T3 p=0.0009

wildtype +T3 vs *DIO3*$^{\Delta 33}$ p=0.022

D120 ANOVA p=0.002

wildtype vs *DIO3*$^{\Delta 33}$ p=0.0015

wildtype vs *DIO3*$^{\Delta 33}$ +T3 p=0.0482

D140 ANOVA p=0.0003

wildtype vs *DIO3*$^{\Delta 33}$ p=0.0002

wildtype vs *DIO3*$^{\Delta 33}$ +T3 p=0.0334

D160 ANOVA p=0.0004

wildtype vs *DIO3*$^{\Delta 33}$ p=0.0024

wildtype vs *DIO3*$^{\Delta 33}$ +T3 p=0.0077

wildtype +T3 vs *DIO3*$^{\Delta 33}$ p= 0.0072

wildtype +T3 vs *DIO3*$^{\Delta 33}$ +T3 p=0.0159

D180 ANOVA p=0.0074

wildtype vs *DIO3*$^{\Delta 33}$ p= 0.0197

D200 ANOVA p=0.4456 (ns).

(B) L/M-opsin+ cell density.

D100 ANOVA p=0.1604 (ns)

D120 ANOVA p=0.0556 (ns)

D140 ANOVA p=0.0556 (ns)

D160 ANOVA p<0.0001

wildtype vs *wildtype* +T3 p<0.0001

wildtype vs *DIO3*$^{\Delta 33}$ p<0.0001

wildtype vs *DIO3*$^{\Delta 33}$ +T3 p<0.0001

D180 ANOVA p=0.0312 (ns)

D200 ANOVA p=0.2987 (ns)

(C) % S-opsin+ cells co-expressing L/M-opsin.

D140 ANOVA $p=0.0034$

wildtype vs *DIO3*$^{\Delta 33}$ $p=0.0043$

wildtype +T3 vs *DIO3*$^{\Delta 33}$ $p=0.0415$;

D160 ANOVA $p=0.0251$

wildtype vs *DIO3*$^{\Delta 33}$ $p=0.0419$

wildtype vs *DIO3*$^{\Delta 33}$ +T3 $p=0.0313$

D180 ANOVA $p<0.0001$

wildtype vs *DIO3*$^{\Delta 33}$ $p= 0.0003$

wildtype vs *DIO3*$^{\Delta 33}$ +T3 $p=0.0001$

wildtype +T3 vs *DIO3*$^{\Delta 33}$ +T3 $p=0.0085$

D200 ANOVA $p=0.0053$

wildtype vs *DIO3*$^{\Delta 33}$ $p=0.0386$

wildtype +T3 vs *DIO3*$^{\Delta 33}$ $p=0.034$

wildtype +T3 vs *DIO3*$^{\Delta 33}$ +T3 $p=0.0469$

(D) Rho+ cell density

D100 Kruskal-Wallis with Dunn's multiple comparisons test $p=0.0197$

wildtype vs *DIO3*$^{\Delta 33}$ +T3 $p= 0.0171$

D120 ANOVA $p=0.0021$

wildtype vs *DIO3*$^{\Delta 33}$ $p=0.0159$

wildtype vs *DIO3*$^{\Delta 33}$ +T3 $p=0.007$

wildtype +T3 vs *DIO3*$^{\Delta 33}$ +T3 $p=0.0307$

D140 ANOVA $p=0.0165$

wildtype vs *DIO3*$^{\Delta 33}$ $p=0.0259$

D160 ANOVA $p=0.0573$ (ns)

D180 ANOVA $p=0.0527$ (ns)

D200 ANOVA $p=0.118$ (ns)

(E) % Rho+ cells co-expressing L/M-opsin.

D100 Kruskal-Wallis with Dunn's multiple comparisons test $p>0.9999$

D120 Kruskal-Wallis with Dunn's multiple comparisons test $p=0.0334$

D140 ANOVA $p<0.0001$

wildtype vs *DIO3*$^{\Delta 33}$ $p<0.0001$

wildtype vs *DIO3*$^{\Delta 33}$ +T3 $p=0.0009$

wildtype +T3 vs *DIO3*$^{\Delta 33}$ $p=0.0017$

wildtype +T3 vs *DIO3*$^{\Delta 33}$ +T3 p=0.0072

D160 ANOVA p=0.0007

wildtype vs *wildtype* +T3 p=0.0008

wildtype vs *DIO3*$^{\Delta 33}$ p=0.0019

wildtype vs *DIO3*$^{\Delta 33}$ +T3 p=0.0017

D180 ANOVA p<0.0001

wildtype vs *DIO3*$^{\Delta 33}$ p<0.0001

wildtype vs *DIO3*$^{\Delta 33}$+T3 p<0.0001

wildtype +T3 vs *DIO3*$^{\Delta 33}$ p<0.0001

wildtype+T3 vs *DIO3*$^{\Delta 33}$ +T3 p<0.0001

D200 ANOVA p<0.0001

wildtype vs *DIO3*$^{\Delta 33}$ p<0.0001

wildtype vs *DIO3*$^{\Delta 33}$ +T3 p=0.0007

wildtype +T3 vs *DIO3*$^{\Delta 33}$ p=0.0023

wildtype +T3 vs *DIO3*$^{\Delta 33}$ +T3 p=0.0443

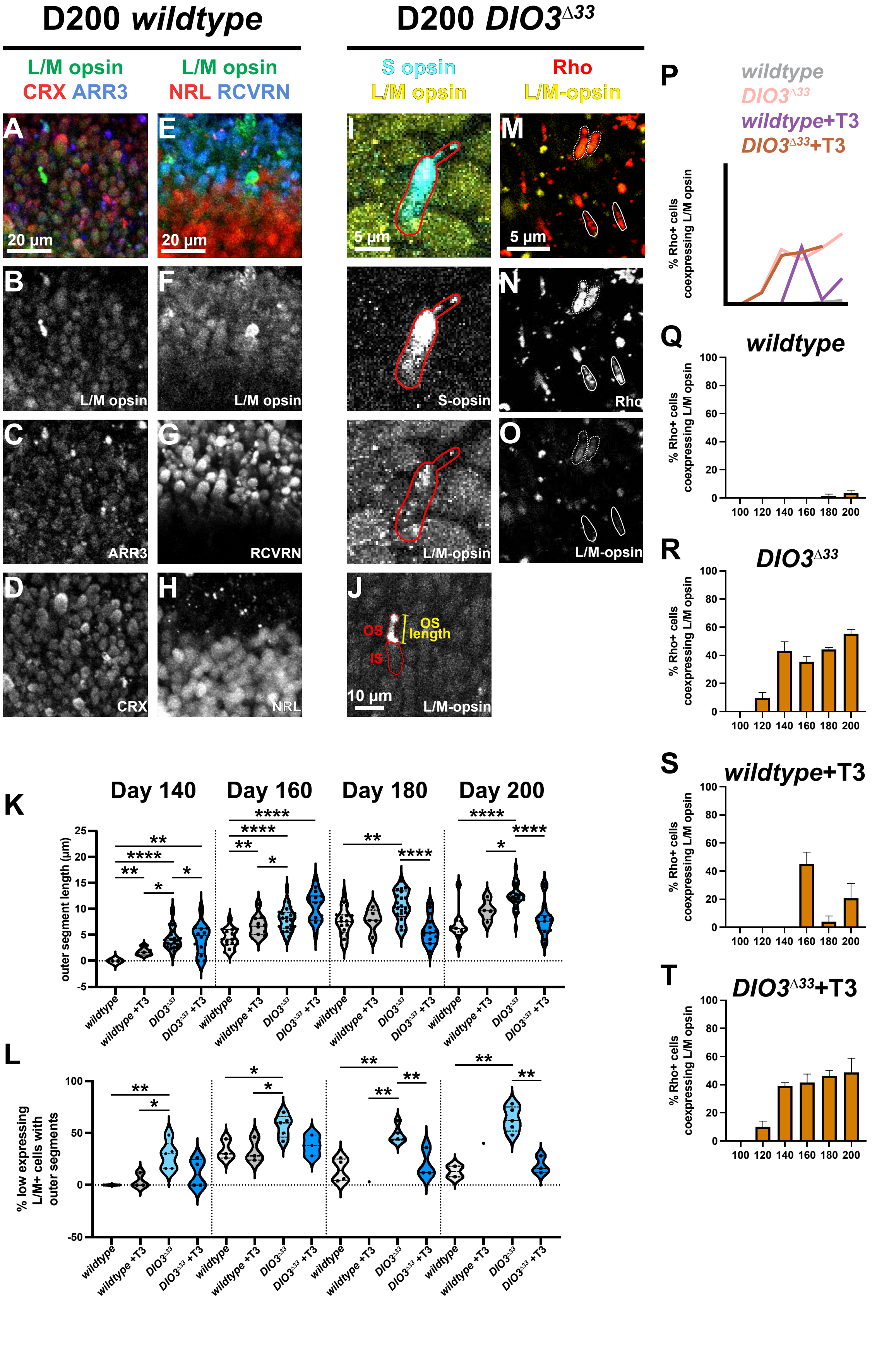

Supplemental Figure 3

Supplemental Fig. 3. Low L/M-opsin+ cells express cone genes, display cone morphologies, and can co-express other opsins.

(A-H) Expression of photoreceptor markers in *wildtype* day 200 organoids.

(A-C) Co-expression of low expressing L/M-opsin+ cells with CRX and ARR3.

(E-H) Co-expression of L/M-opsin with RCVRN but not NRL.

(I) $DIO3^{\Delta 33}$ day 200 S-opsin+ cell co-expressing L/M-opsin+ cell. Red outline delineates cell boundary.

(J) Low L/M-opsin+ expressing cell in $DIO3^{\Delta 33}$ day 200 organoid with an inner segment (IS) and outer segment (OS). OS length (yellow) measured for (K).

(K-L) Outer segment length (K) and % of low expressing L/M-opsin+ cells with OS (L) in *wildtype* (light grey), *wildtype*+T3 (dark grey), $DIO3^{\Delta 33}$ (light blue), $DIO3^{\Delta 33}$ +T3 (dark blue) organoids at day 140, day 160, day 180, and day 200. Error bars represent SEM, two-tailed t-test performed to determine statistical significance. Unlabeled comparisons are not significant ($p>0.05$). *wildtype* D140 N=4, D160 N=3, D180 N=4, D200 N=2; *wildtype* +T3 D140 N=1, D160 N=3, D180 N=1, D200 N=1. $DIO3^{\Delta 33}$ D140 N=5, D160 N=5, D180 N=5, D200 N=5. $DIO3^{\Delta 33}$ +T3 D140 N=4, D160 N=3, D180 N=3, D200 N=3.

(K) D140: *wildtype* vs *wildtype* +T3 p= 0.002, *wildtype* vs $DIO3^{\Delta 33}$ p=0.0006, *wildtype* vs $DIO3^{\Delta 33}$ +T3 p=0.0141, *wildtype* +T3 vs $DIO3^{\Delta 33}$ p=0.0187.

D160: *wildtype* vs *wildtype* +T3 p= 0.0042, *wildtype* vs $DIO3^{\Delta 33}$ p<0.0001, *wildtype* vs $DIO3^{\Delta 33}$ +T3 p<0.0001, *wildtype* +T3 vs $DIO3^{\Delta 33}$ +T3 p=0.0015, $DIO3^{\Delta 33}$ vs $DIO3^{\Delta 33}$ +T3 p=0.0035.

D180: *wildtype* vs $DIO3^{\Delta 33}$ p=0.001, $DIO3^{\Delta 33}$ vs $DIO3^{\Delta 33}$ +T3 p<0.0001. D200: *wildtype* vs $DIO3^{\Delta 33}$ p<0.0001, *wildtype* +T3 s $DIO3^{\Delta 33}$ p=0.046, $DIO3^{\Delta 33}$ vs $DIO3^{\Delta 33}$ +T3 p<0.0001. n=5 cells of this morphology analyzed per organoid per timepoint and condition.

(L) D140: *wildtype* vs $DIO3^{\Delta 33}$ p=0.004, *wildtype*+T3 vs $DIO3^{\Delta 33}$ p=0.0278. D160 *wildtype* vs $DIO3^{\Delta 33}$ p=0.0218, *wildtype* +T3 vs $DIO3^{\Delta 33}$ p=0.0255. D180: *wildtype* vs $DIO3^{\Delta 33}$ p=0.0011, *wildtype* +T3 vs $DIO3^{\Delta 33}$ p=0.0071, $DIO3^{\Delta 33}$ vs $DIO3^{\Delta 33}$ +T3 p=0.0097. D200 *wildtype* vs $DIO3^{\Delta 33}$ p=0.0031, $DIO3^{\Delta 33}$ vs $DIO3^{\Delta 33}$ +T3 p=0.0014.

(M-O) Single z image of cells that co-express Rho and L/M-opsin in a $DIO3^{\Delta 33}$ organoid at day 200. Solid outline marks Rho+ only cells, dashed outline represents Rho&L/M-opsin co-expressing cells.

(P) Graphical representation of (P-T) quantifications.

(Q-T) Quantification of %Rho+ cells expressing L/M-opsin in (Q) *wildtype*, (R) $DIO3^{\Delta 33}$, (S) *wildtype* +T3, and (T) $DIO3^{\Delta 33}$+T3. Organoids without rods were excluded from the analysis. Error bars represent SEM.

Wildtype D100 N=0, D120 N=3, D140 N=5, D160 N=3, D180 N=3, D200 N=4.
DIO3$^{\Delta 33}$ D100 N=2, D120 N=8, D140 N=6, D160 N=10, D180 N=12, D200 N=9.
wildtype +T3 D100 N=2, D120 N=2, D140 N=2, D160 N=4, D180 N=2, D200 N=3.
DIO3$^{\Delta 33}$+T3 D100 N=4, D120 N=3, D140 N=4, D160 N=4, D180 N=3, D200 N=3.
(U-V) Calculated total PR density for (U) wildtype and (V) *DIO3*$^{\Delta 33}$ organoids. Total PR density was calculated by summing the average density of cells expressing S-opsin, M/L-opsin, and Rho and subtracting the average proportion that co-expressed multiple opsins. Data for S-opsin, M/L-opsin, and Rho-expressing cells are as in **Fig. 2** and **Fig S2.**

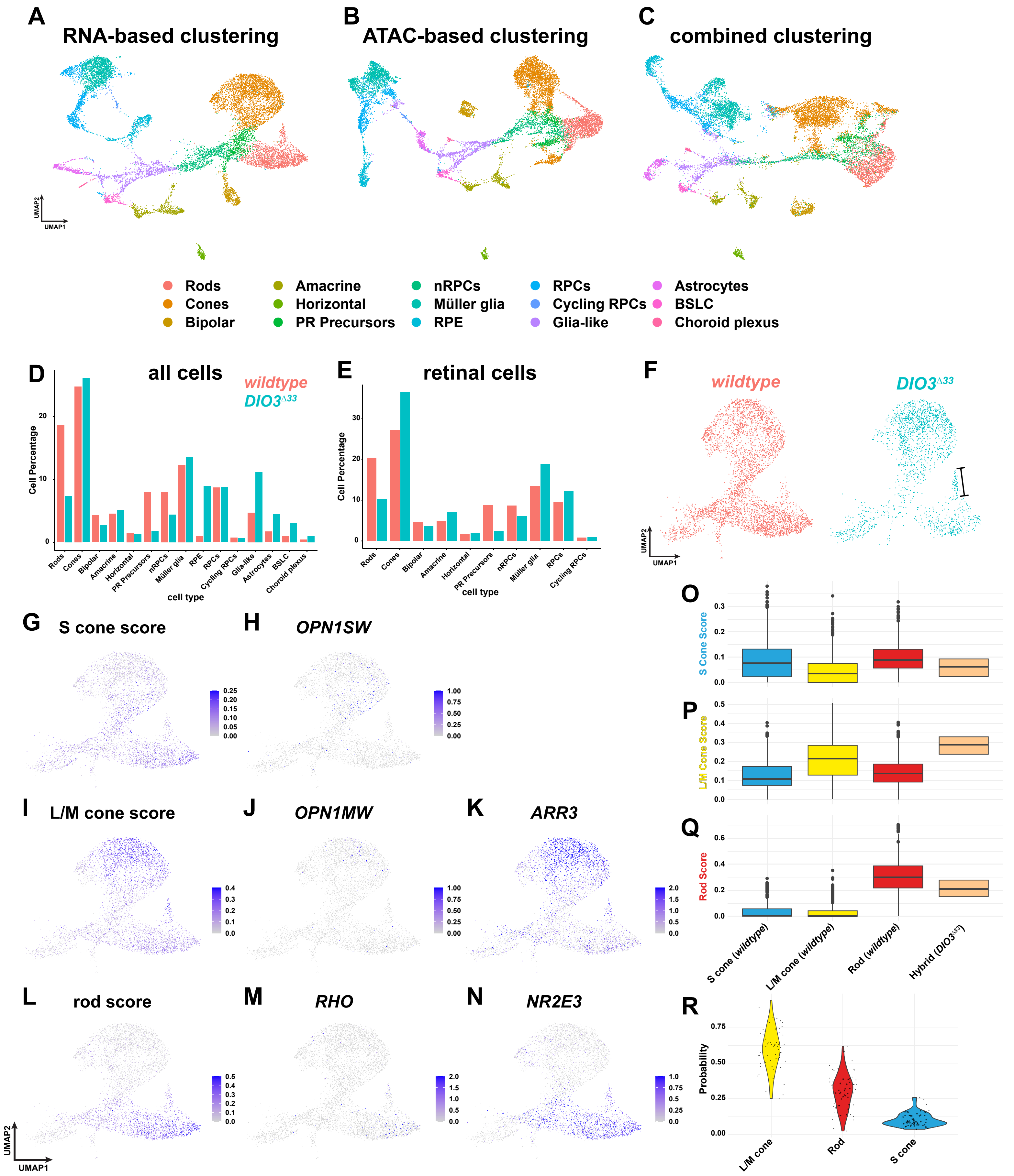

Supplemental Figure 4

Supplemental Fig. 4. **Characterization of *DIO3*$^{\Delta 33}$ mutant organoids with single nucleus multiomics.**

(A) UMAP clustering of *wildtype* and *DIO3*$^{\Delta 33}$ mutant organoids based on single nucleus RNA-sequencing.

(B) UMAP clustering of *wildtype* and *DIO3*$^{\Delta 33}$ mutant organoids based on single nucleus ATAC-sequencing.

(C) UMAP clustering of *wildtype* and *DIO3*$^{\Delta 33}$ mutant organoids with contributions from both the RNA- and ATAC-sequencing.

(D) Cell proportions for all cells in *wildtype* (coral) and *DIO3*$^{\Delta 33}$ (teal) organoids.

(E) Cell proportions for all retinal cells in *wildtype* (coral) and *DIO3*$^{\Delta 33}$ (teal) organoids.

(F) UMAP of photoreceptor lineage cells (nRPCs, PR precursors, cones, rods) in *wildtype* (coral) and *DIO3*$^{\Delta 33}$ (teal) organoids. Bracket highlights putative hybrid cells.

(G-M) Gene expression heatmaps overlaid on (F) UMAP. (G) Compiled S-cone score, (H) *OPN1SW*, (I) L/M-cone score, (J) *OPN1MW*, (K) *ARR3* (L) rod score, (M) *RHO*, and (N) *NR2E3.*

(O-Q) Box plot of (O) S cone, (P) L/M cone, and (Q) rod scores of *wildtype* S cones (blue), *wildtype* L/M cones (yellow), *wildtype* rods (red), and putative hybrid cells (orange) from *DIO3*$^{\Delta 33}$, as bracketed in (F).

(R) Violin plots of predicted probabilities for each photoreceptor class: L/M-cone (yellow), rod (red) or S-cone (blue) in putative hybrid cells from *DIO3*$^{\Delta 33}$ organoids, based on a random forest classifier.

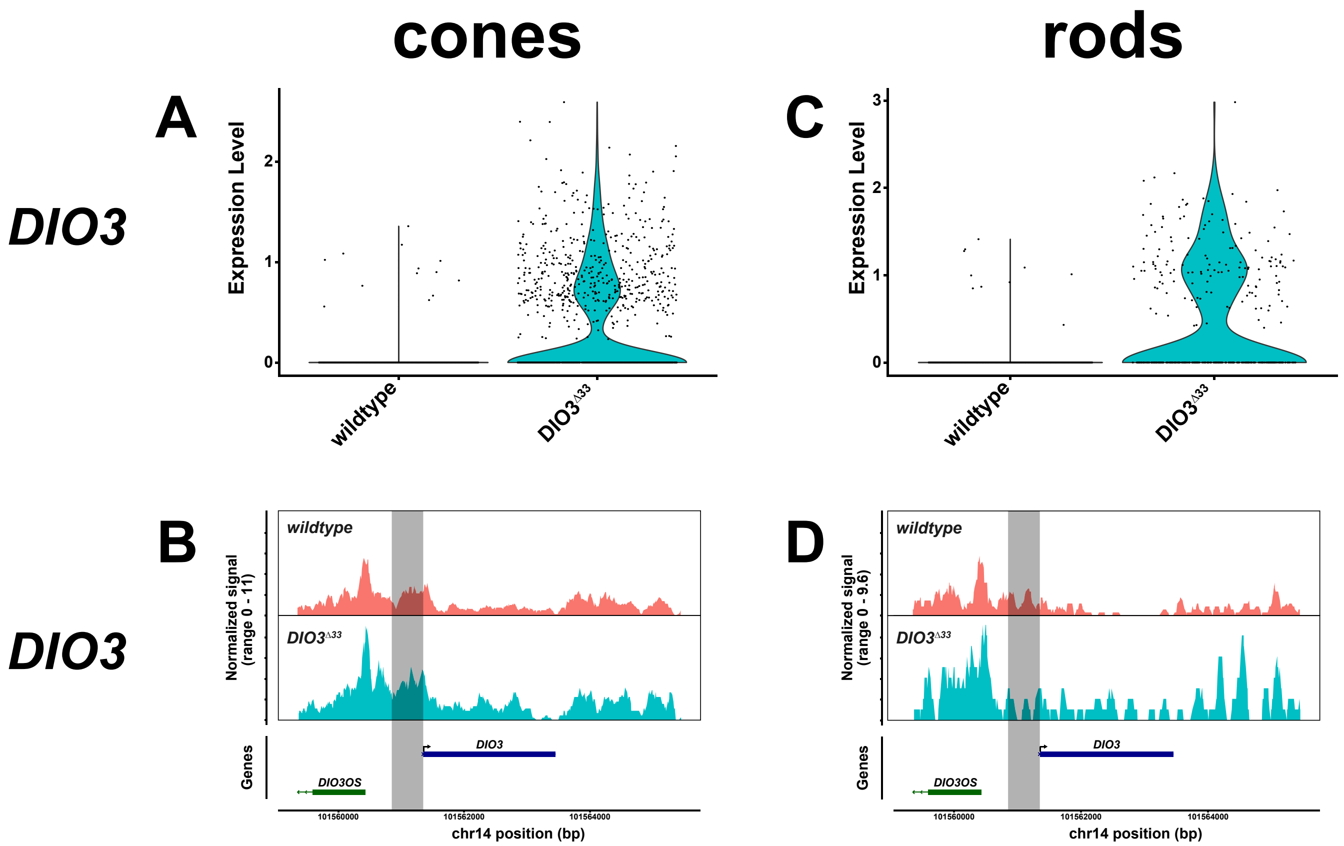

Supplemental Figure 5

Supplemental Fig. 5. Accessibility and expression of *DIO3* in photoreceptors.

(A, C) Expression of *DIO3* in cones (A) and rods (C) in *wildtype* and *DIO3*$^{\Delta 33}$ organoids.

(B, D) Accessibility of *DIO3* in cones (B) and rods (D) in *wildtype* and *DIO3*$^{\Delta 33}$ organoids.

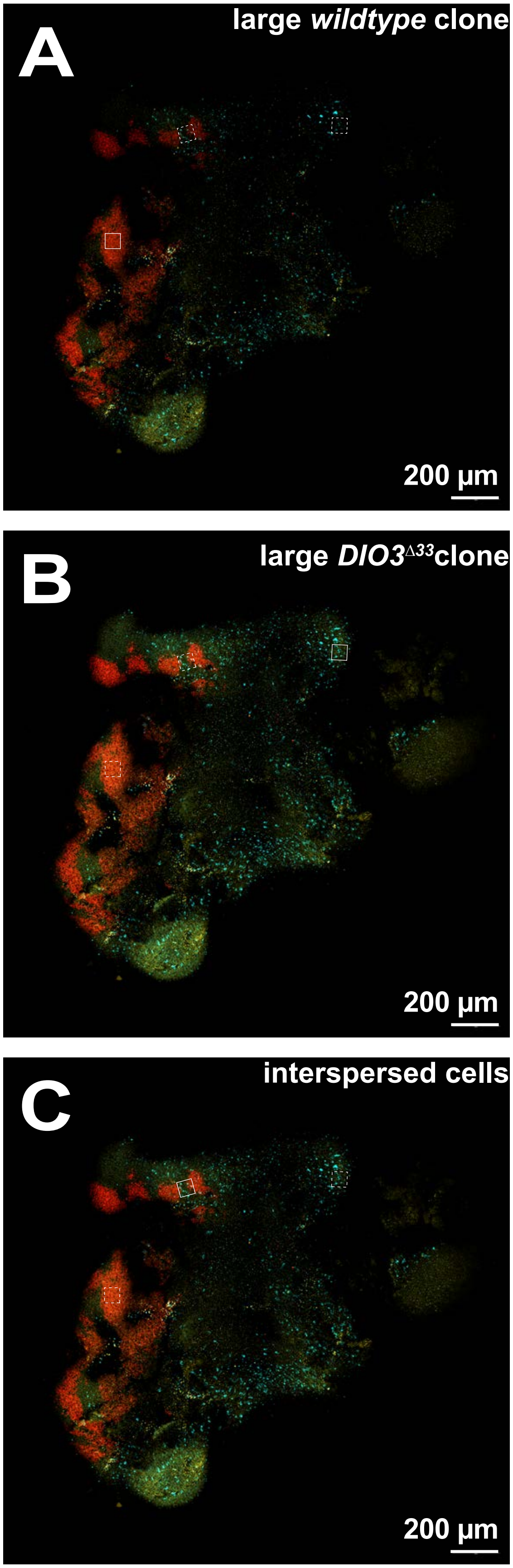

Supplemental Figure 6

Supplemental Fig. 6. Representative chimeric retinal organoid

(A-C) D160 chimeric retinal organoid as in Fig 5 H-J. tdTomato+ regions are H9 *wildtype CRX:tdTomato* cells. tdTomato+ regions are H7 *DIO3*$^{\Delta 33}$ cells. Due to the 3D shape of the organoid, images were taken from three different focal planes. Solid box indicates region of interest in that focal plane. Dotted box indicates region of interest from a different focal plane. Solid boxed region in A corresponds with **Fig 5H**. Boxed region in B corresponds with **Fig 5I**. Boxed region in C corresponds with **Fig 5J**.

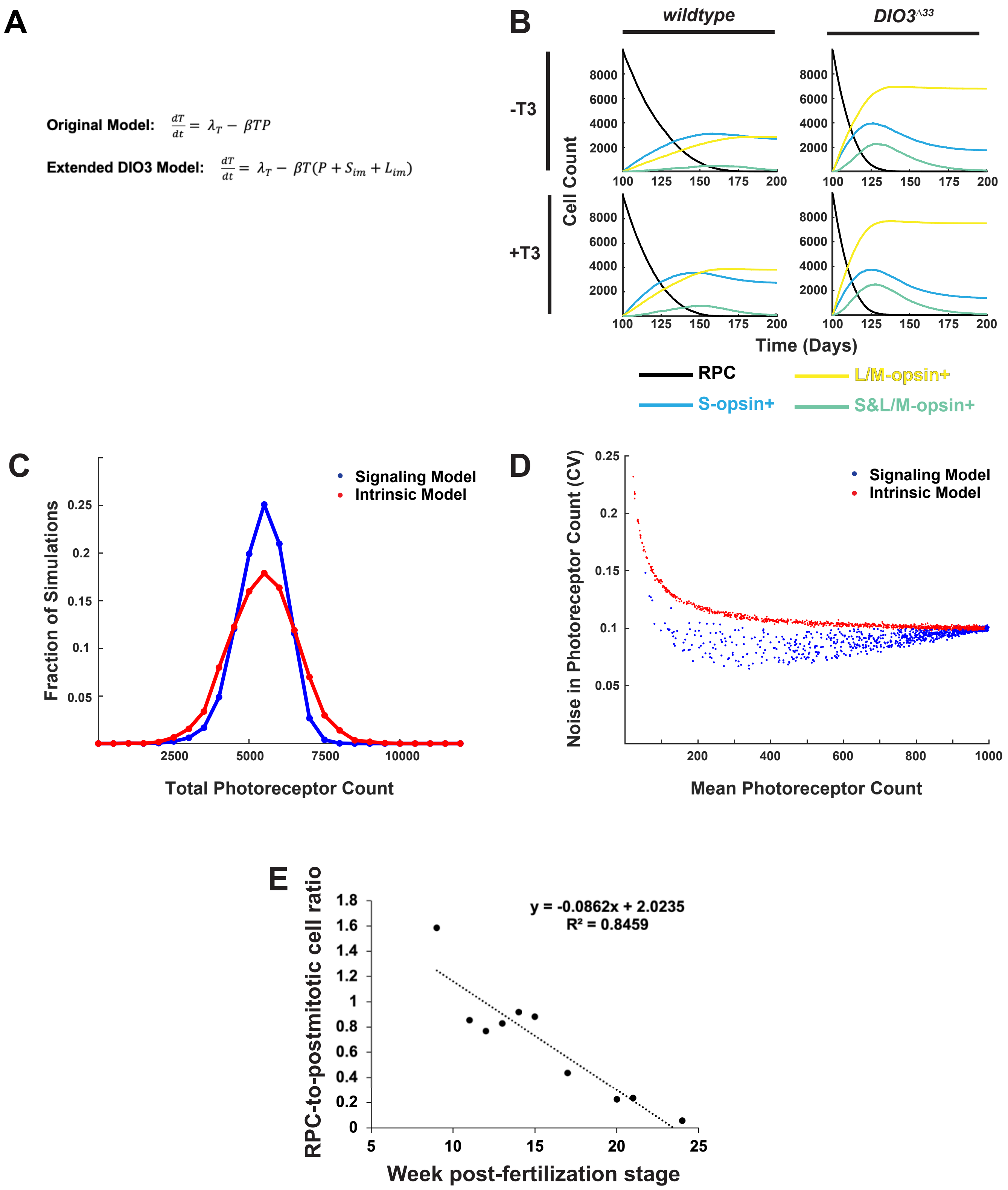

Supplemental Figure 7

Supplemental Fig. 7. Additional modeling and data analysis

(A) Updated model assumptions. In the version of the model presented in the main text, only progenitor cells (*P*) express DIO3. The degradation rate of T3 (*T*) is therefore proportional only to *P* ('Original Model'). In the modified model presented here, *P*, *Sim* and L_{im} also contribute to T3 degradation ('Extended DIO3 Model'). All other model assumptions and parameters are identical to those of the main text model.

(B) Simulation of cone specification dynamics in Extended DIO3 model. Wild-type and $DIO3^{D33}$ organoids (left and right columns, respectively) in the absence and presence (top and bottom rows, respectively) of exogenous T3 are shown. Curves depict counts of RPC (black), L/M cones (yellow), S cones (blue) and S&L/M double-expressing cones (green).

(C) T3 signaling filters noise in initial RPC abundance in Extended DIO3 model. Histograms show the total number of photoreceptors (S + L/M cones) specified in 5000 simulations seeded with random number of initial RPCs in signaling (blue) and cell intrinsic (red) models.

(D) Exploration of model parameter space in Extended DIO3 model. Simulations (n = 1000) were performed for signaling (blue) and cell-intrinsic (red) models with randomly-sampled parameter values. Each dot represents the performance of 50 replicate simulations with a given parameter set.

(E) Progressive decrease in RPC-to-postmitotic cell ratio during human fetal retinal development, based on snRNA-seq data from (Zuo et al., 2024b).

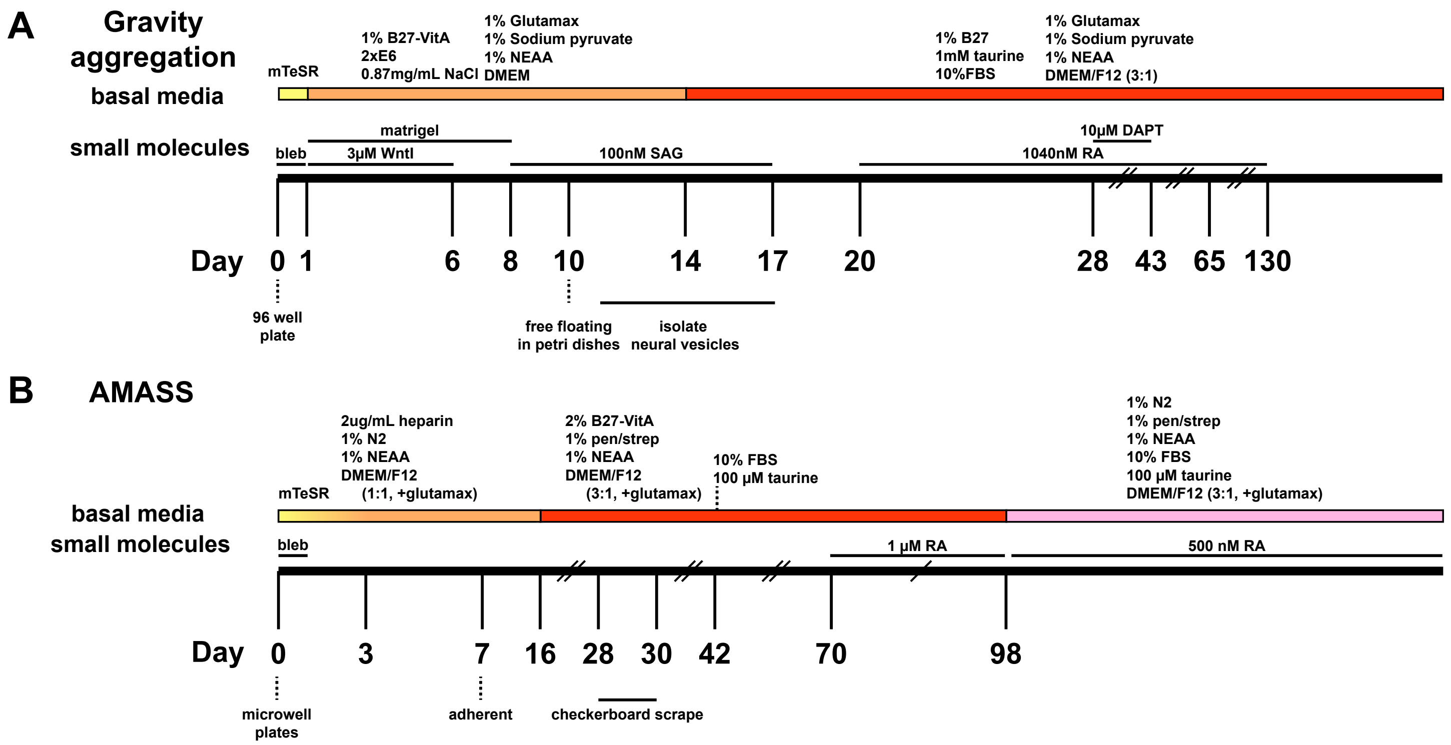

Supplemental Figure 8

Supplemental Fig. 8. Methods of organoid differentiation.

(A) Gravity aggregation adapted from (Eldred et al., 2018; Hussey et al., 2024) , used to generate organoids in Fig. 1D-G, Fig. 4E-F, and Supp. Fig. 1F-G.

(B) AMASS differentiation protocol adapted from (Cowan et al., 2020) , used to generate all other organoids for this study. We benchmarked photoreceptor development between gravity aggregation and AMASS methods and note no differences in developmental timing or photoreceptor constituency. All experimental manipulations were performed with internal controls to minimize variability between differentiations.
