# Supplementary material for: DIO3 coordinates photoreceptor development timing and fate stability in human retinal organoids
Source: Genes Dev. 2026 Jan 1;40(1-2):70–93. doi: 10.1101/gad.352924.125 (PMC12758142; doi:10.1101/gad.352924.125)
Supplement: Supplement 2 [file Supplemental_Model.docx]

**A stochastic model of T3-regulated photoreceptor specification in retinal organoids**

Here we detail the computational model used to simulate thyroid hormone-mediated control of retinal organoid development presented in **Fig. 6** of the main text. This document will walk through model construction (Section 1: ‘*Model Construction’*), build intuition for the dynamics of cell fate specification in wild-type and *DIO3* mutant organoids (Section 2: ‘*Simulation of Cell Fate Specification Dynamics’*), and present a speculative discussion of how thyroid hormone regulation could be used to achieve robust organoid development (Section 3: ‘*Robustness in Retinal Organoid Development*).

1. **Model Construction**
   1. Structure and key assumptions

In our model, feedback control over photoreceptor specification is exerted through the regulation of thyroid hormone abundance. Specifically, retinal progenitor cells (*P*) express DIO3, an enzyme that degrades active thyroid hormone (*T*). As organoid development proceeds, DIO3-expressing progenitors are depleted, resulting in an increasing concentration of *T* and acceleration of photoreceptor specification. As we will demonstrate, these features are sufficient to reproduce the observed photoreceptor specification dynamics of wild-type and *DIO3* mutant organoids and suggest that thyroid hormone-mediated feedback could be used to suppress noise in photoreceptor specification.

The model makes the following mechanistic assumptions:

1. Thyroid hormone (*T*) induces the differentiation of L/M-opsin-expressing (*L*) and S-opsin-expressing (*S*) photoreceptor cells from retinal progenitor cells (*P*).
2. *P* cells can also differentiate into non-cone fates (collectively referred to as *A* cells) in a thyroid-independent manner.
3. Terminal commitment to *S* and *L* fates is not instantaneous. Immature *S* and *L* cells (*S_im_* and *L_im_* cells, respectively) are generated from *P* cells, and irreversibly commit to mature fates with a constant rate ($\lambda_{m}$).
4. Immature photoreceptor cells can enter into a ‘double expressing’ state (*SL*) in which both *L/M* and *S* opsins are expressed. These cells probabilistically mature into terminal *S* or *L* cells with constant, *T*-independent per-cell rates.
5. *T* is imported into the organoid at a constant rate ($\lambda_{T}$) and is degraded by the action of DIO3. Our measurements of intra-organoid *T* concentration ( < 60 pM) are two orders of magnitude below the empirical K_m_ of DIO3 (Kuiper et al., 2003). We therefore assume that the rate of DIO3-mediated T3 degradation is linear with respect to T3.
6. DIO3 is expressed in *P* cells, but not in differentiated photoreceptor cells.
7. Rates of *S_im_* and *L_im_* differentiation are saturable in *T*. For simplicity, we relate *T* to the rate of differentiation with a Hill function (Hill constant = 1).

The flow of cells through available states and their accompanying rates of each event can be graphically summarized as follows:


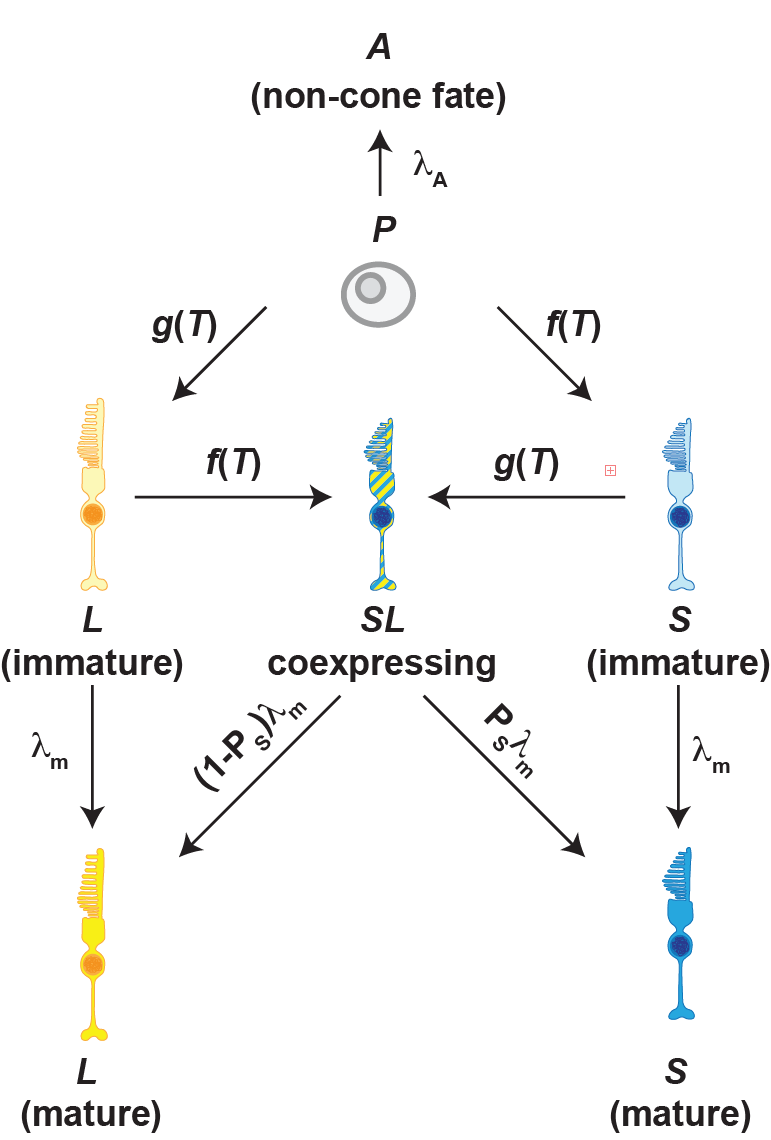


**Figure 1.1**. **Model schematic.** In each simulation, all cells begin as undifferentiated *P* cells and can proceed along differentiation trajectories as summarized by the arrows. The expression above each arrow denotes the form of the per-cell rate of transition along that pathway. Simulations conclude when all cells have adopted terminal fates (i.e. *L, S,* or *A*).

We simulate cell differentiation dynamics as a stochastic birth-death process. All simulations presented in the main text were carried out using a modified Gillespie algorithm in which *T* dynamics were simulated deterministically via numerical integration and cell fate decisions occur stochastically. Below, we formalize each permissible event, its state-dependent rate and the resulting step sizes for each system component.

Deterministic components:

1. **T3 Dynamics**: $\frac{dT}{dt}= \lambda_{T}-$ $\beta TP$

Stochastic components:

1. **Immature *S* Birth**: $\left\{ P,S_{im},L_{im,}L,S,SL,T,A \right\}\underset{\to}{\frac{\lambda_{S}T}{{kSat}_{S}+T}P}\left\{ P-1,S_{im}+1,L_{im,}L,S,SL,T,A \right\}$
2. **Immature *L* Birth***:* $\left\{ P,S_{im},L_{im,}L,S,SL,T,A \right\}\underset{\to}{\frac{\lambda_{L}T}{{kSat}_{L}+T}P}\left\{ P-1,S_{im},L_{im}+1,L,S,SL,T,A \right\}$
3. ***SL* Birth (from *S_im_*)**: $\left\{ P,S_{im},L_{im,}L,S,SL,T,A \right\}\underset{\to}{\frac{\lambda_{L}T}{{kSat}_{L}+T}S_{im}}\left\{ P,S_{im}-1,L_{im,}L,S,SL+1,T,A \right\}$
4. ***SL* Birth (from *L_im_*)**: $\left\{ P,S_{im},L_{im,}L,S,SL,T,A \right\}\underset{\to}{\frac{\lambda_{S}T}{{kSat}_{S}+T}L_{im}}\left\{ P,S_{im},L_{im,}-1,L,S,SL+1,T,A \right\}$
5. ***S* maturation**: $\left\{ P,S_{im},L_{im,}L,S,SL,T,A \right\}\underset{\to}{\lambda_{m}S_{im}}\left\{ P,S_{im}-1,L_{im},L,S+1,SL,T,A \right\}$
6. ***L* maturation**: $\left\{ P,S_{im},L_{im,}L,S,SL,T,A \right\}\underset{\to}{\lambda_{m}L_{im}}\left\{ P,S_{im},L_{im}-1,L+1,S,SL,T,A \right\}$
7. ***SL* matures to *S***: $\left\{ P,S_{im},L_{im,}L,S,SL,T,A \right\}\underset{\to}{{P_{S}\lambda}_{m}SL}\left\{ P,S_{im},L_{im},L,S+1,SL-1,T,A \right\}$
8. ***SL* matures to *L***:$\left\{ P,S_{im},L_{im,}L,S,SL,T,A \right\}\underset{\to}{{(1-P_{S})\lambda}_{m}SL}\left\{ P,S_{im},L_{im},L+1,S,SL-1,T,A \right\}$
9. ***A* birth:** $\left\{ P,S_{im},L_{im,}L,S,SL,T,A \right\}\underset{\to}{\lambda_{A}P}\left\{ P-1,S_{im},L_{im},L,S,SL,T,A+1 \right\}$
   1. Event Rates

In the table below, we summarize the rationale behind the form of each event rate.

| **Process** | **Form** | **Intuition** |
| --- | --- | --- |
| *T* import | $\lambda_{T}$ | We assume that *T* is transported into the organoid at a constant rate. Note that *T* import is simulated deterministically. |
| *T* degradation | $\beta TP$ | *T* is degraded with first-order kinetics by DIO3-expressing *P* cells. The effective rate constant of degradation is the per-cell rate of *T* degradation ($\beta)$ multiplied by the number of degrading cells (*P*). Note that *T* degradation is simulated deterministically. |
| *S_im_* Birth | $\frac{\lambda_{S}T}{{kSat}_{S}+T}P$ | The per-cell rate of $P\underset{\to}{}S_{im}$ differentiation ($f\left( T \right)$in diagram above) is a saturating function of *T* for which the half-maximal value ($\lambda_{S}/2)$occurs at *T =* ${kSat}_{S}$*.* |
| *L_im_* Birth | $\frac{\lambda_{L}T}{{kSat}_{L}+T}P$ | The per-cell rate of $P\underset{\to}{}L_{im}$ differentiation ($g\left( T \right)$ in diagram above) is a saturating function of *T* for which the half-maximal value ($\lambda_{L}/2)$occurs at *T =* ${kSat}_{L}$*.* |
| *SL* Birth (from *S_im_*) | $\frac{\lambda_{L}T}{{kSat}_{L}+T}S_{im}$ | The per-cell rate of $S_{im}\underset{\to}{}{SL}$ differentiation ($g\left( T \right)$in diagram above) is a saturating function of *T* for which the half-maximal value ($\lambda_{L}/2)$occurs at *T =* ${kSat}_{L}$*.*  We note that this is identical to the rate of $P\underset{\to}{}L_{im}$ differentiation. Intuitively, this reflects an assumption that expression of *L-*related genes is independent of adoption of the $S_{im}$ fate. |
| *SL* Birth (from L*_im_*) | $\frac{\lambda_{B}T}{{kSat}_{B}+T}L_{im}$ | The per-cell rate of $L_{im}\underset{\to}{}{SL}$ differentiation ($f\left( T \right)$in diagram above) is a saturating function of *T* for which the half-maximal value ($\lambda_{L}/2)$occurs at *T =* ${kSat}_{L}$*.*  We note that this is identical to the rate of $P\underset{\to}{}S_{im}$ differentiation. Intuitively, this reflects an assumption that expression of *S-*related genes is independent of adoption of the $L_{im}$ fate. |
| *S_im_* maturation | $\lambda_{m}S_{im}$ | Maturation occurs with a constant rate ($\lambda_{m}$) per cell. |
| *L_im_* maturation | $\lambda_{m}L_{im}$ | Maturation occurs with a constant rate ($\lambda_{m}$) per cell. |
| *SL* matures to *S* | ${P_{S}\lambda}_{m}SL$ | *SL* Maturation occurs with the same constant rate ($\lambda_{m}$) per cell as with *S_im_* and L*_im_*, but probabilistically resolves into a mature *S* cell with probability $P_{S}$. |
| *SL* matures to *L* | ${{(1-P}_{S})\lambda}_{m}SL$ | *SL* Maturation occurs with the same constant rate ($\lambda_{m}$) per cell as with *S_im_* and L*_im_*, but probabilistically resolves into a mature *L* cell with probability $1-P_{S}$. |
| *A* birth | $\lambda_{A}P$ | Progenitor cells adopt a non-cone alternative fate with a constant, *T*-independent rate per cell ($\lambda_{A}$). |

- 1. Model Parameter Values

| **Parameter** | **Description** | **Value** | **Intuition for value** |
| --- | --- | --- | --- |
| $\lambda_{T}$ | Rate of T3 import | 24 pM/day | With $\beta$, sets initial pseudo-steady state *T* concentration at 1 pM |
| $\beta$ | Rate constant for T3 degradation | 2.4 x 10^-3^ day^-1^ | With $\lambda_{T}$, sets initial pseudo-steady state *T* concentration at 1 pM and initial *T* average lifetime at 1 hour. |
| $\lambda_{S}$ | Maximal rate of *S_im_* differentiation from *P* | 7.2 x 10^-2^  day^-1^ | Sets average $P\underset{\to}{}S_{im}$waiting time at 14 days at saturating *T* concentrations. |
| ${kSat}_{S}$ | T3 concentration at which rate of *S_im_* differentiation is half-maximal | 5 pM | Rate of $P\underset{\to}{}S_{im}$differentiation is half maximal when *P* decreases to 2000 cells. |
| $\lambda_{L}$ | Maximal rate of L*_im_* differentiation from *P* | 2.5 x 10^-1^ day^-1^ | Sets average $P\underset{\to}{}L_{im}$waiting time at 4 days at saturating *T* concentrations. |
| ${kSat}_{L}$ | T3 concentration at which rate of L*_im_* differentiation is half-maximal | 40 pM | Rate of $P\underset{\to}{}L_{im}$differentiation is half maximal when *P* decreases to 250 cells. |
| $\lambda_{A}$ | Rate of *A* differentiation from *P* | 2.5 x 10^-2^ day^-1^ | Sets average $P\underset{\to}{}A$waiting time at 40 days. |
| $\lambda_{mat}$ | Maturation rate for L*_im_*, S*_im_* and *SL* cells. | 6.1 x 10^-2^ day^-1^ | Average residence time in the immature state is 16 days. |
| $P_{S}$ | Probability of a *SL* co-expressing cell resolving into a mature *S* cell. | 0.1 | 90% of *SL* cells resolve into *L* mature state. |

1. **Simulation of Cell Fate Specification Dynamics**

The model aims to capture several observations presented in the main text. Specifically, it was designed to accommodate the following:

1. *S* and *L/M* cone specification is induced by thyroid hormone signaling.
2. In *wildtype* organoids, *S* cone specification precedes *L/M* cone specification, but the *L/M* population ‘overtakes’ the *S* population late in development.
3. *DIO3* mutant organoids exhibit accelerated photoreceptor specification, increased proportion of *L/M* cones, and transient appearance of *SL* co-expressing cells.
4. Addition of exogenous *T* moderately accelerates specification dynamics in *wildtype* organoids, but leaves *DIO3^∆33^* mutant dynamics unchanged.

Below, we reproduce the simulations from **Fig. 6** of the main text to discuss model performance with respect to these benchmarks.

2.1. *wildtype* dynamics


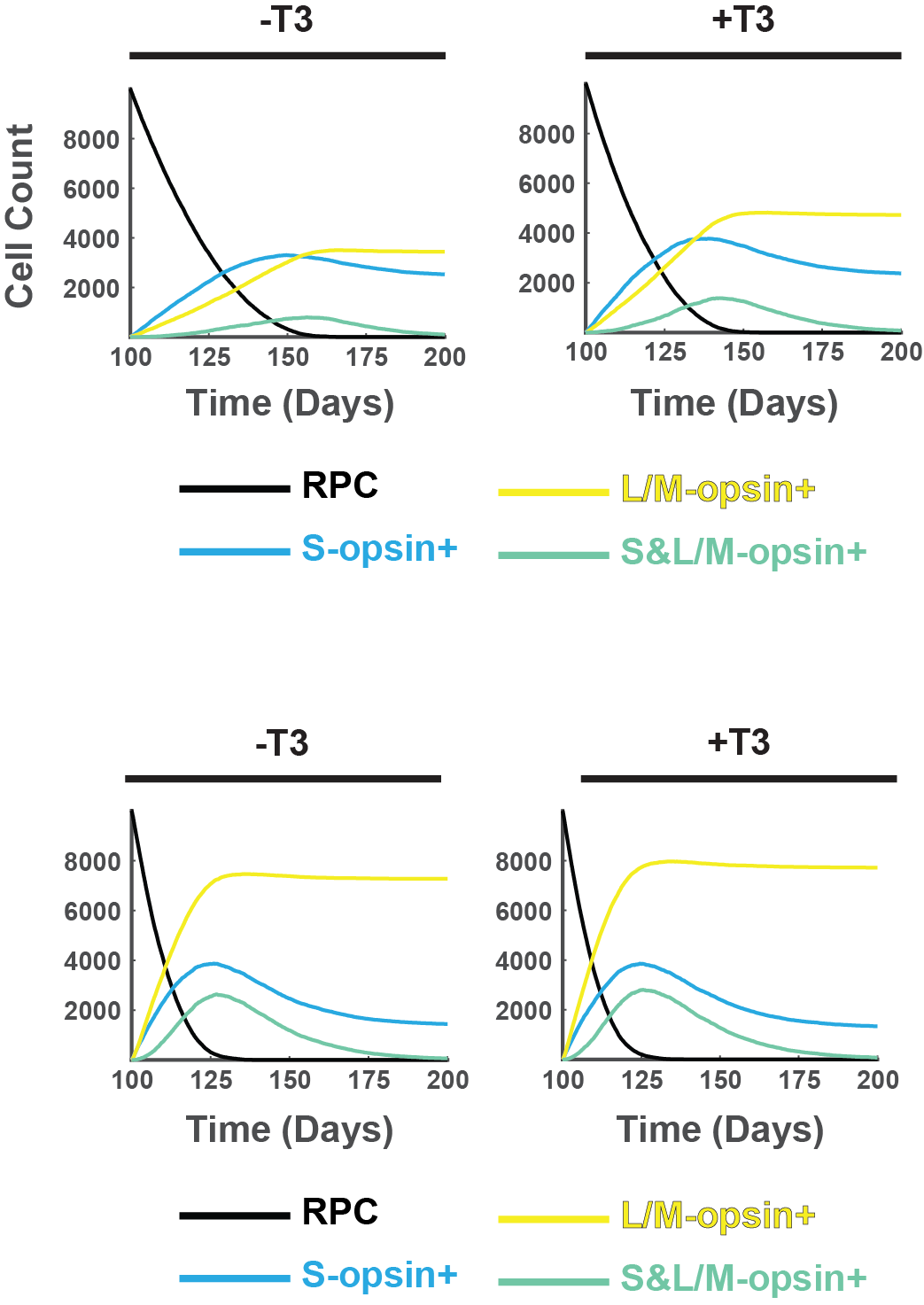


**Figure 2.1**. **Simulated photoreceptor specification dynamics in *wildtype* organoids.** The figure reproduces the wild-type panels from **Fig. 6B** of the main text. The left simulation represents unperturbed *wildtype* development, and the right represents a *wildtype* organoid grown with exogenous T3 added to the culture medium. Black, blue, yellow and green curves depict *P, S, L* and *SL* cell type copy numbers, respectively. Each simulation begins with 10^4^ undifferentiated *P* cells.

The ‘*-*T3’ simulation above (**Fig. 2.1**, left panel) captures the key features of photoreceptor specification dynamics in unperturbed *wildtype* organoids. Specifically, *S* cones appear first, with abundance peaking at approximately day 140. *L/M* cones appear later and ultimately overtake *S* in abundance. The model also predicts the observation of a small number (~15% of the total pool) of cones that co-express *S* and *L/M* opsins. Finally, the model captures the modest acceleration of specification dynamics observed when exogenous T3 is added to culture medium (**Fig. 2.1**, right panel).

The characteristic dynamics of photoreceptor specification in *wildtype* organoids can be understood intuitively in terms of *T-*dependent model rates. *S* cells appear before *L* cells because they are induced by lower concentrations of *T* (i.e. ${kSat}_{S}< {kSat}_{L}$)*. L* cells ultimately ‘catch up’ to *S* due to two factors. First, the *maximal* rate of *L* differentiation at the high *T* concentrations that occur late in development is greater than the corresponding rate for *S* cells (i.e. $\lambda_{L}>\lambda_{S}$). Second, the *SL* co-expressing cells preferentially resolve into mature *L* cells (i.e. $P_{S}=0.1$). This has the net effect of ‘redirecting’ immature *S* cells into *L* fates via the *SL* co-expressing state. This second effect also explains why *S* cell numbers slightly decline after their peak.

Our model also offers a simple explanation for the existence of *SL* co-expressing cells. We posit that immature *S* and *L* cells retain the ability to ‘fire’ a second *T-*dependent developmental program—expression of *L/M* or *S* opsins, respectively— until they mature into terminally differentiated photoreceptors. Co-expressing cells thus arise late in development when *T* concentrations are high enough that the rates of *S* or *L* program initiation compete with the rate of cone maturation. Consistent with this intuition, *SL* cell abundance is elevated by both the addition of exogenous *T3* (**Fig. 2.1**, right panel) and removal of DIO3 (see below).

2.2. *DIO3^∆33^* dynamics


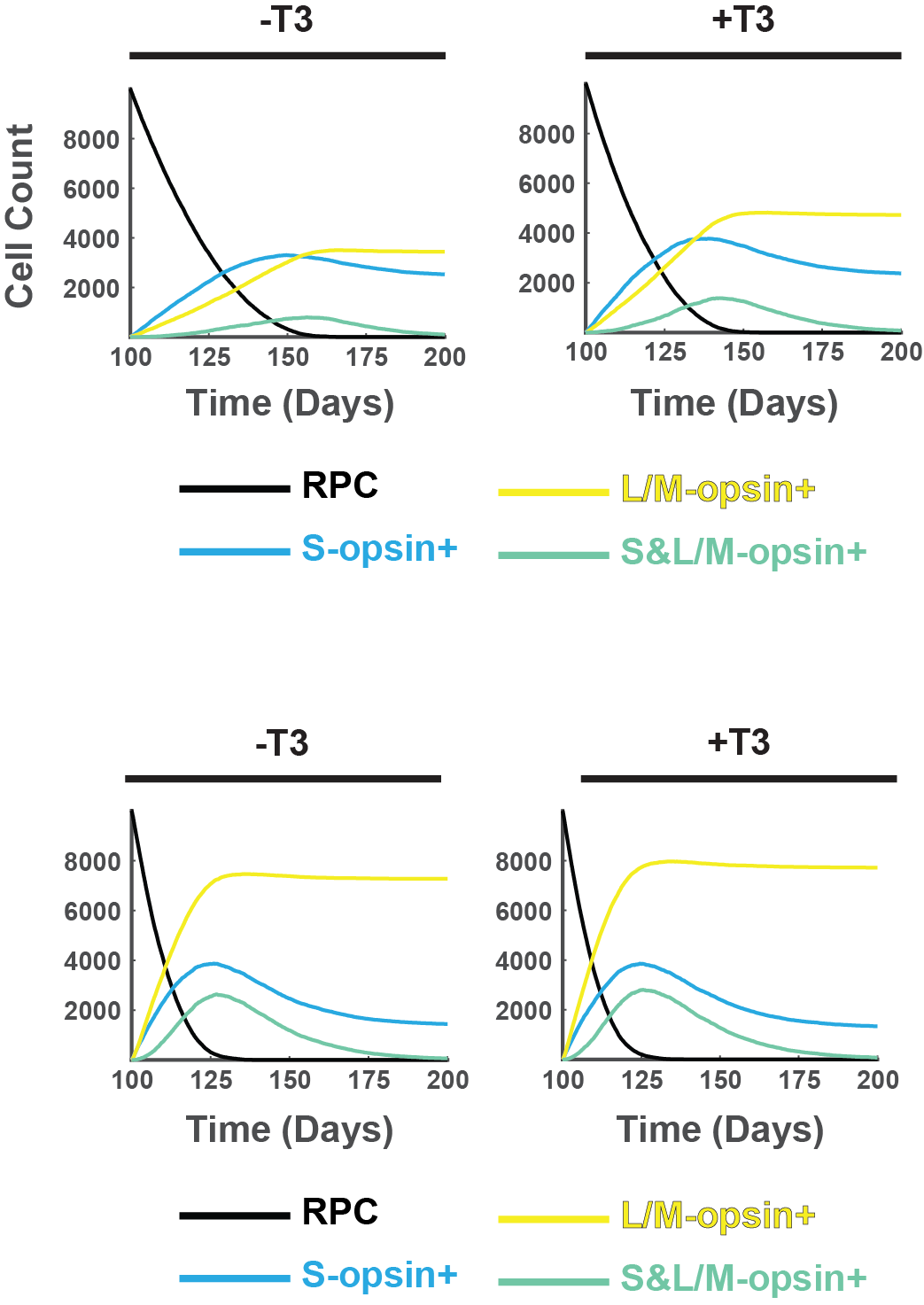


**Figure 2.2**. **Simulated photoreceptor specification dynamics in *DIO3^∆33^* mutant organoids.** The figure reproduces the *DIO3^∆33^* mutant panels from Fig. 6B of the main text. The left simulation represents an unperturbed *DIO3^∆33^* mutant organoid, and the right represents *a DIO3^∆33^* mutant organoid grown with exogenous T3 added to the culture medium. Black, blue, yellow and green curves depict *P, S, L,* and *SL* cell type copy numbers, respectively. Each simulation begins with 10^4^ undifferentiated *P* cells.

Our model reproduces the key features of photoreceptor specification dynamics in both perturbed and unperturbed *DIO3^∆33^* mutant organoids. Specifically, it correctly captures the accelerated timeline of photoreceptor specification, increased proportion of *L* cells, as well as increased proportion of *SL* co-expressing cells. Additionally, it correctly predicts that addition of exogenous T3 has no effect on the dynamics or final composition of the photoreceptor pool.

These features can again be understood in terms of the *T-*dependence of model rate functions. The acceleration of organoid development results from increased *T* abundance; the *DIO3^∆33^* mutant exhibits a 94% reduction in the rate constant of *T* degradation, leading to a ~20-fold increase in *T* concentration relative to *wildtype* organoids. This results in elevated rates of *S* and *L* cell differentiation, as well as a higher abundance of *SL* co-expressing cells. This increase also explains why addition of exogenous *T* does not alter development in mutant organoids. The 20-fold increase in baseline *T* levels pushes the rates of *S* and *L* differentiation into the saturation regime, so that additional *T* has little to no effect on development.

1. **Robustness in Retinal Organoid Development**

The material presented above demonstrates that the simple kinetic principles of our model are sufficient to reproduce the photoreceptor specification dynamics of *wildtype* and *DIO3^∆33^* mutant organoids. In this section, we turn to the question of why T3-mediated feedback control might be useful for development. Below, we develop the hypothesis that this control confers robustness to retinal organoid development.

- 1. Constructing a cell-intrinsic model

For the arguments below, it will be useful to contrast the behavior of our ‘signaling’ model with that of a ‘cell-intrinsic’ model that lacks feedback. The states and permissible transitions of the model are assumed to be identical to those laid out in **Fig. 1.1**. However, the rates with *T-*dependence become *T*-independent constants. We formalize the cell-intrinsic stochastic birth-death process as follows:

1. **Immature *S* Birth**: $\left\{ P,S_{im},L_{im,}L,S,SL,T,A \right\}\underset{\to}{\lambda_{S}P}\left\{ P-1,S_{im}+1,L_{im,}L,S,SL,T,A \right\}$
2. **Immature *L* Birth***:* $\left\{ P,S_{im},L_{im,}L,S,SL,T,A \right\}\underset{\to}{\lambda_{L}P}\left\{ P-1,S_{im},L_{im}+1,L,S,SL,T,A \right\}$
3. ***SL* Birth (from *S_im_*)**: $\left\{ P,S_{im},L_{im,}L,S,SL,T,A \right\}\underset{\to}{\lambda_{L}S_{im}}\left\{ P,S_{im}-1,L_{im,}L,S,SL+1,T,A \right\}$
4. ***SL* Birth (from *L_im_*)**: $\left\{ P,S_{im},L_{im,}L,S,SL,T,A \right\}\underset{\to}{\lambda_{S}L_{im}}\left\{ P,S_{im},L_{im,}-1,L,S,SL+1,T,A \right\}$
5. ***S* maturation**: $\left\{ P,S_{im},L_{im,}L,S,SL,T,A \right\}\underset{\to}{\lambda_{m}S_{im}}\left\{ P,S_{im}-1,L_{im},L,S+1,SL,T,A \right\}$
6. ***L* maturation**: $\left\{ P,S_{im},L_{im,}L,S,SL,T,A \right\}\underset{\to}{\lambda_{m}L_{im}}\left\{ P,S_{im},L_{im}-1,L+1,S,SL,T,A \right\}$
7. ***SL* matures to *S***: $\left\{ P,S_{im},L_{im,}L,S,SL,T,A \right\}\underset{\to}{{P_{S}\lambda}_{m}SL}\left\{ P,S_{im},L_{im},L,S+1,SL-1,T,A \right\}$
8. ***SL* matures to *L***:$\left\{ P,S_{im},L_{im,}L,S,SL,T,A \right\}\underset{\to}{{(1-P_{S})\lambda}_{m}SL}\left\{ P,S_{im},L_{im},L+1,S,SL-1,T,A \right\}$
9. ***A* birth:** $\left\{ P,S_{im},L_{im,}L,S,SL,T,A \right\}\underset{\to}{\lambda_{A}P}\left\{ P-1,S_{im},L_{im},L,S,SL,T,A+1 \right\}$
   1. Comparing robustness of signaling and cell-intrinsic models

In **Fig. 6C** and **D** of the main text, we argue that T3-mediated feedback confers robustness to noise in the initial number of *P* cells in the early organoid. This was shown for a specific set of model parameters in **Fig. 6C**; when the initial number of *P* cells in each replicate simulation is a random variable, the final number of photoreceptor cells is more tightly distributed in the signaling model than in the cell-intrinsic model. In this sense, T3 feedback enables this noise to be ‘filtered out’, leading to a more precise number of final photoreceptors across replicate realizations. Finally, we demonstrate that this property is not a product of the specific parameter sets chosen for **Fig. 6 A-C**, but rather a generic property of the feedback system across many distinct parameter sets (**Fig. 6D**, reproduced below as **Fig. 3.1**). Not only does it appear that the signaling model *can* outperform the cell-intrinsic model, but that the intrinsic model matches the *worst achievable* performance by the models with feedback. In the following sections we develop intuition for this observation.


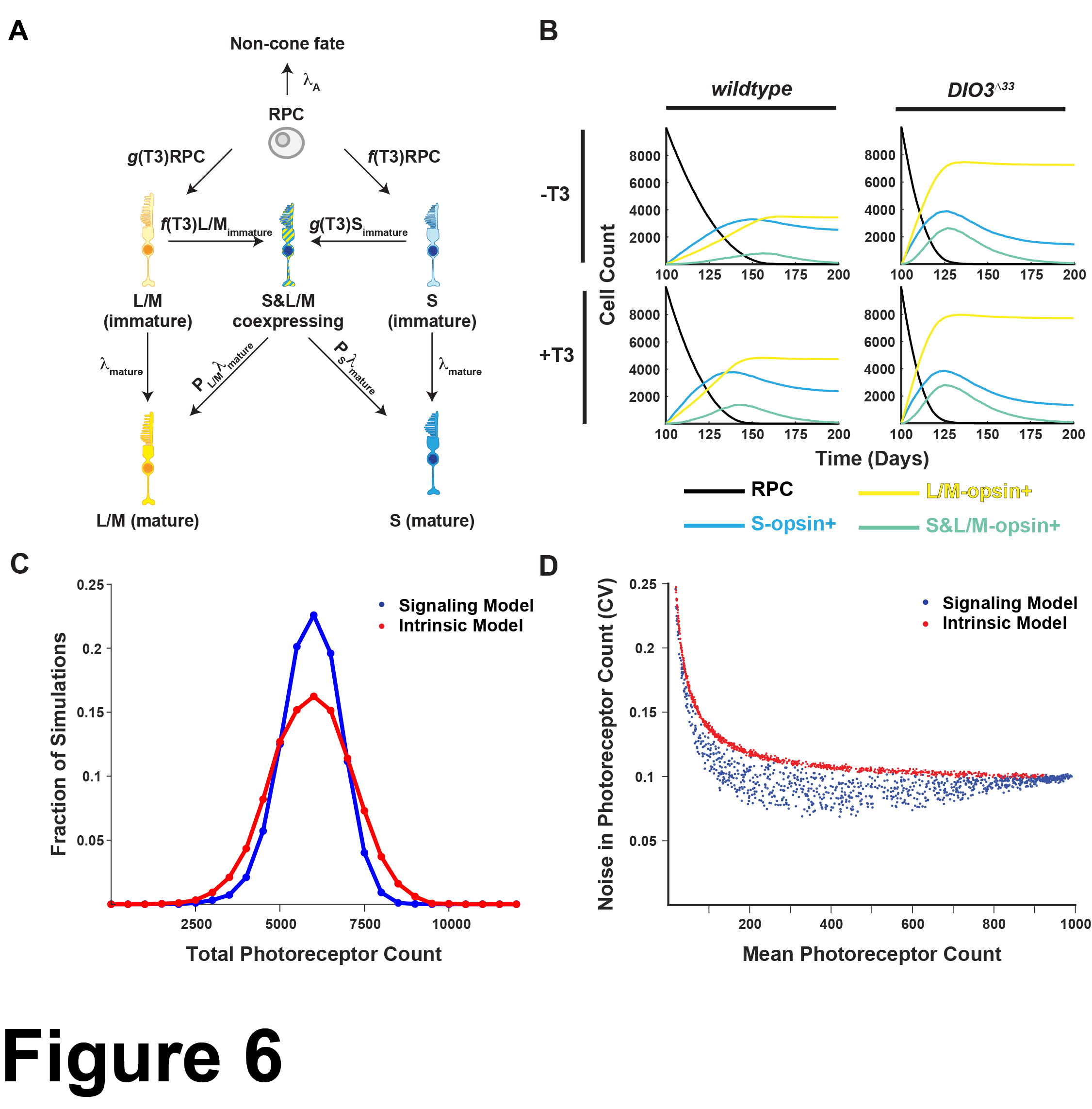


**Figure 3.1**. **Enhanced developmental robustness in signaling models.** Exploration of model parameter space. Simulations (n = 1000) were performed for signaling (blue) and cell-intrinsic (red) models with randomly sampled parameter values. Each dot represents the performance of 50 replicate simulations with a unique parameter set.

- 1. Limiting behaviors of cell-intrinsic and signaling models

The empirical exploration of model capabilities presented in **Fig. 3.1** suggests the existence of two smooth, limiting curves in mean-CV space: a ‘noisy’ upper limit and a ‘precise’ lower limit that intersect at low and high mean photoreceptor counts. As we explore below, identifying the origins of these limits will help to explain how the signaling model filters out noise in initial *P* abundance.

To develop intuition for the upper limit curve shape, we recognize that the intrinsic model— from the point of view of the final photoreceptor count— can be viewed as a simple binomial cut of a randomly-varying initial number of progenitors. That is, each *P* cell present at the beginning of the simulation has only two possible end fates: cone (i.e. *S* or *L* cells) or non-cone (*A* cells, which also encapsulate the possibility of a cell death fate). Since the per-cell rates of each differentiation event constant, the probability that a given *P* cell adopts these fates are $\frac{\lambda_{S}+ \lambda_{L}}{\lambda_{S}+ \lambda_{L}+ \lambda_{A}}$ and $\frac{\lambda_{A}}{\lambda_{S}+ \lambda_{L}+ \lambda_{A}}$ , respectively. Each *P* cell makes an independent decision according to these probabilities, resulting in binomial statistics for a given initial number of progenitors. The amount of noise in photoreceptor abundance will be larger at low mean photoreceptor count— reflecting both the variation in initial *P* abundance and low-number noise due to small photoreceptor differentiation rates— and asymptotically approach the noise in *P* seeding at higher average counts when the randomness of individual fate decisions is negligible. This simplified binomial model is superimposed onto the plot below (**Fig. 3.2**, black curve), and appears to capture the upper noise limit’s shape.

The lower limit represents a diametrically-opposed kinetic regime. In the intrinsic model, the cone and non-cone differentiation rates are all first-order with respect to *P*. The lower limit can be captured, by contrast, with a simplified model in which cones and non-cones differentiate with zero- and first-order kinetics, respectively. In this limiting model, the rate of non-cone differentiation is still P-dependent ($\lambda_{A}P$), while the rates of *S* and *L* differentiation are *P*-independent (i.e. $\lambda_{S}$ and $\lambda_{L}$, respectively). Simulating a simplified model with these zero/first-order assumptions results in a curve that appears to represent a lower bound for the behavior of the signaling model (**Fig. 3.2**, magenta curve).


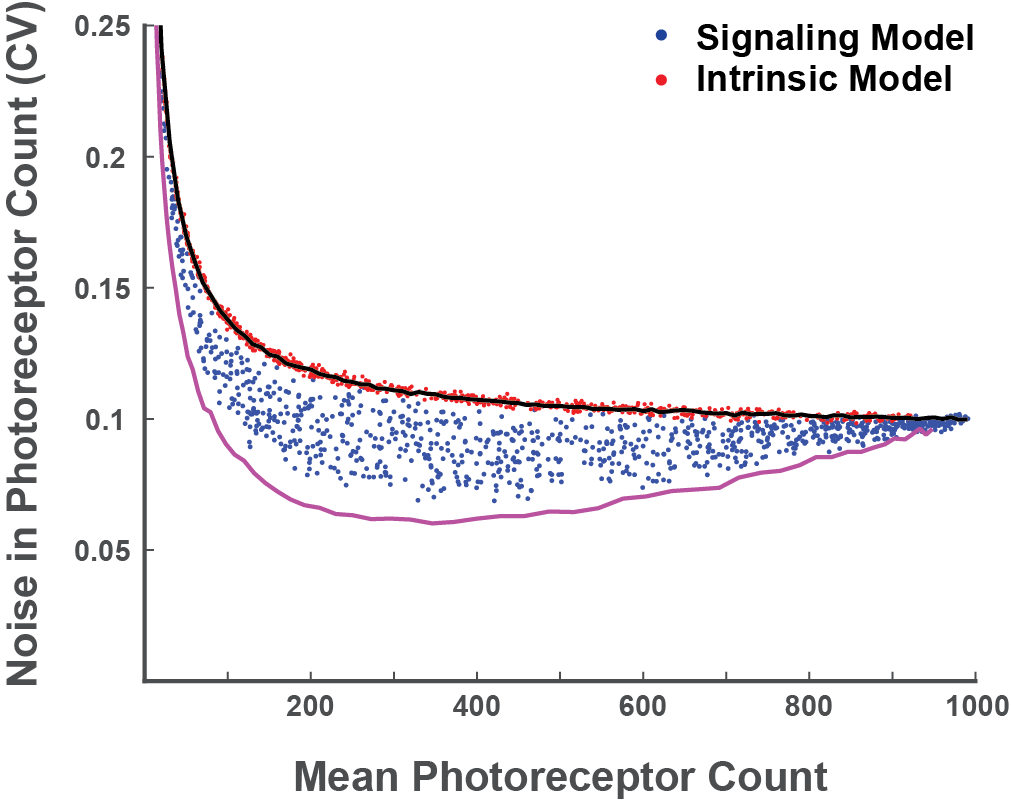


**Figure 3.2**. **Limiting behavior of signaling and cell-intrinsic models.** Blue and red dots depict simulations with randomly-sampled parameter sets as in **Fig. 3.1**. Black curve represents the binomial approximation to the cell intrinsic model. The magenta curve represents the first/zero-order approximation to the signaling model. These approximations appear to capture the limiting behaviors of the cell-intrinsic and signaling models.

- 1. Intuition behind noise filtering in the signaling model

The analysis presented in the previous section demonstrates that the best-performing parameter sets in the signaling model occupy a regime where non-cone and cone differentiation rates are first- and zero-order with respect to *P*. This raises two key questions. First, why does this regime perform well? Second, how can the signaling model achieve zero-order differentiation rates for *L* and *S* cells? We address both questions in order below.

The mechanism of noise suppression in the idealized signaling model can be understood in terms of variation in the time required to consume all *P* cells. In a simple model with only zero-order differentiation of *P* cells, the variation in initial numbers of *P* translates directly into variation in the time it takes to complete the process: twice as many *P* cells take twice as long to consume when the rate of differentiation is constant (i.e. zero-order). By contrast, a model with only first-order differentiation kinetics achieves some buffering. Because the size of the *P* pool declines exponentially in this model, having twice as many initial cells results in only a log(2) increase in the completion time. Importantly, combining both first- and zero-order removal further improves this timing precision over the first-order only model. Indeed, this combination of first- and zero-order removal has been proposed to underlie the tight timing of cell fate commitments in bacterial cells (Lord et al., 2019; Norman et al., 2013).

The precise timing behavior of the signaling model is the key to the noise suppression illustrated in **Fig. 3.1**. The combination of first-order consumption of *P* to make *A* cells and zero-order consumption of *P* to make *S* and *L* cells means that the time required to complete differentiation will be similar even when the initial number of *P* cells widely varies. During this tightly-distributed time, the zero-order kinetics of cone differentiation steadily accumulate new cones in a constant, clock-like manner. These two effects together mean that the final count of cones is tightly controlled: the number of ‘ticks’ achieved by a clock over a narrowly-distributed period of time will be narrowly-distributed.

Importantly, this noise-buffering behavior is not accessible to the cell-intrinsic model because all differentiation events have first-order kinetics. This can be illustrated with a series of simulations in which the number of initial *P* cells is systematically varied. In the cell-intrinsic model, changes in the number of *P* cells translates directly into changes in the final number of photoreceptor cells (i.e. the relationship is linear, **Fig. 3.3**, red dots). By contrast, this relationship is sub-linear for the signaling molecule; an X% change in initial *P* abundance leads to a less-than X% change in final photoreceptor count (**Fig. 3.3**, blue dots). This differential sensitivity to changes in *P* underlies the noise buffering properties that we propose.


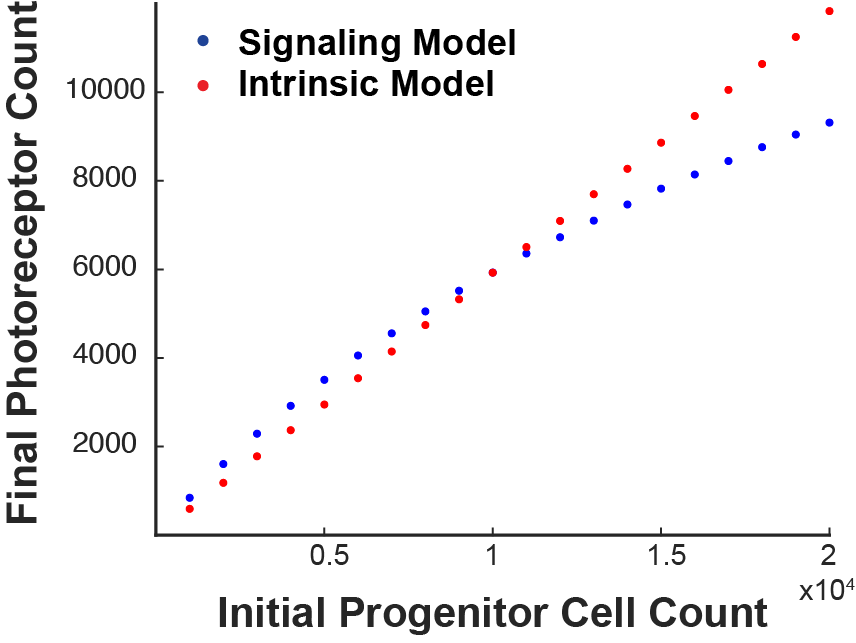


**Figure 3.3**. **Model sensitivity to changes in initial progenitor cell abundance.** This plot compares the mean number of final photoreceptors (*L* and *S* cells) to the initial number of progenitors (*P*) cells for the signaling (blue) and cell-intrinsic (red) models.

The best performing signaling molecule parameter sets mimic a situation in which *L* and *S* differentiation rates are zero-order with respect to *P*. How can this situation arise when the mathematical forms of the corresponding rate functions depend on *P*? The answer lies in the relationship between *P* and *T* abundance. *T* levels increase as development proceeds due to the depletion of *P* cells; only *P* cells degrade *T*, so thyroid hormone levels increase as the degrading cells differentiate. This relationship creates an effective cancellation: the rate of photoreceptor differentiation is simultaneously *increased* by rising *T* levels and *decreased* by falling P levels. Outside of the saturation regime— i.e. where $T< {kSat}_{S}, {kSat}_{L}$— these changes precisely cancel, leaving the rate of photoreceptor differentiation unchanged. As a result, these rates do not change with *P* abundance, creating the (effectively) zero-order relationship required for noise suppression.

- 1. A note on non-cone fates

An interesting feature of the signaling model is that the ‘alternative’ non-cone fate plays an integral role to the noise filtering described above. In order for the signaling domain to effectively reject noise in *P* abundance, there must be a pathway of *P* consumption with a first-order (i.e. *T*-independent) rate constant. This pathway is therefore not a ‘catch all’ for other fates accessible to *P* cells, but rather an important part of T3-regulated photoreceptor development. We also note that the identity of this ‘alternative’ fate does not matter for this role. Indeed, even cell death could serve this purpose. In this case, the noise in initial *P* numbers would be ‘absorbed’ by the dying cell population so that photoreceptor specification can be precise. In this sense, cell death could be regarded not as a wasteful side product of development, but rather as the cost of precision.

**References:**

Kuiper, G.G.J.M., Klootwijk, W., Visser, T.J., 2003. Substitution of Cysteine for Selenocysteine in the Catalytic Center of Type III Iodothyronine Deiodinase Reduces Catalytic Efficiency and Alters Substrate Preference. Endocrinology 144, 2505–2513. https://doi.org/10.1210/en.2003-0084

Lord, N.D., Norman, T.M., Yuan, R., Bakshi, S., Losick, R., Paulsson, J., 2019. Stochastic antagonism between two proteins governs a bacterial cell fate switch. Science 366, 116–120. https://doi.org/10.1126/science.aaw4506

Norman, T.M., Lord, N.D., Paulsson, J., Losick, R., 2013. Memory and modularity in cell-fate decision making. Nature 503, 481–486. https://doi.org/10.1038/nature12804
